# Unraveling topoisomerase IA gate dynamics in presence of PPEF and its preclinical evaluation against multidrug-resistant pathogens

**DOI:** 10.1038/s42003-023-04412-1

**Published:** 2023-02-18

**Authors:** Vikas Maurya, Raja Singh, Reman Kumar Singh, Stuti Pandey, Pooja Yadav, Palak Parashar, Rajni Gaind, Kshatresh Dutta Dubey, G. Naresh Patwari, Vibha Tandon

**Affiliations:** 1 Special Centre for Molecular Medicine, Jawaharlal Nehru University, New Delhi, 110067 India; 2 Department of Chemistry, IIT Bombay, Powai, Mumbai, Maharashtra 400076 India; 3 Vardhaman Medical College Hospital, Safdarjung Hospital, New Delhi, 110029 India; 4 Department of Chemistry, School of Natural Sciences, Shiv Nadar University, Gautam Buddha Nagar, Uttar Pradesh 201314 India

**Keywords:** Target validation, Antimicrobial resistance, Computational models

## Abstract

Type IA topoisomerases maintain DNA topology by cleaving ssDNA and relaxing negative supercoils. The inhibition of its activity in bacteria prevents the relaxation of negative supercoils, which in turn impedes DNA metabolic processes leading to cell death. Using this hypothesis, two bisbenzimidazoles, PPEF and BPVF are synthesized, selectively inhibiting bacterial TopoIA and TopoIII. PPEF stabilizes the topoisomerase and topoisomerase-ssDNA complex, acts as an interfacial inhibitor. PPEF display high efficacy against ~455 multi-drug resistant gram positive and negative bacteria. To understand molecular mechanism of inhibition of TopoIA and PPEF, accelerated MD simulation is carried out, and results suggested that PPEF binds, stabilizes the closed conformation of TopoIA with –6Kcal/mol binding energy and destabilizes the binding of ssDNA. The TopoIA gate dynamics model can be used as a tool to screen TopoIA inhibitors as therapeutic candidates. PPEF and BPVF cause cellular filamentation and DNA fragmentation leading to bacterial cell death. PPEF and BPVF show potent efficacy against systemic and neutropenic mouse models harboring *E. coli*, VRSA, and MRSA infection without cellular toxicity.

## Abstract (web summary)

Two potent inhibitors PPEF and BPVF of bacterial TopoIA enzymes function by impairing DNA binding and thus DNA cleavage and relaxation activities of bacterial topoisomerase I enzymes.

## Introduction

Antibiotic resistance is a global crisis that renders numerous emergent infections and contagions unresponsive to established antibiotic regimes. This prompted the WHO in 2017 to publish a list of antibiotic-resistant “priority pathogens”(critical, high, and medium priority), underscoring nine bacterial species for research and development of new antibiotics^1^. In 2019, the WHO projected that drug-resistant diseases caused about 700,000 deaths per year and could rise to about 10 million deaths by 2050. These antibiotic-resistant pathogens include carbapenem-resistant *Acinetobacter baumannii* and *Enterobacteriaceae*; penicillin- and cephalosporin-resistant extended-spectrum β-lactamase (ESBL) producing *Escherichia coli*; vancomycin-resistant *Enterococcus faecium*; vancomycin- and methicillin-resistant *Staphylococcus aureus*; fluoroquinolone-resistant *Salmonella* and *Shigella* spp.; and penicillin-resistant *Streptococcus* spp. Experimental results indicate that the cyclic antibiotic treatments as culpable for the upsurge of bacterial tolerance and aggravating the evolution of intense antibiotic resistance^2^. Furthermore, most new antibiotics are modified structures of extant antibiotics, which primes bacterial survival following resistance to these structures. Therefore, combating antibiotic resistance requires the development of therapeutic agents with new targets in pathogenic bacteria. A recently published review detailed 19 new pharmacophores as antibiotic candidates, six in phase I, nine in phase II, and four in phase III clinical trials. For instance, ridinilazole, a bisbenzimidazole that inhibits bacterial cell division, is in phase II clinical trial. Another compound, SPR720, an ethyl urea benzimidazole that inhibits GyrB and ParE, is in phase I of clinical trials^3^. These reports support the development of bisbenzimidazoles as bactericidal agents against Gram-positive and Gram-negative bacteria.

Bisbenzimidazoles, 2-(3,4-dimethoxyphenyl)-5-(5-(4 methylpiperazin-1-yl)-1H-benzimidazol-2-yl]-1H-benzimidazole and 2′-(4-ethoxyphenyl)-5-(4-propylpiperazin-1-yl)-1H,1′H-2,5′-bibenzo(d)imidazole (PPEF) were already reported as potent *E. coli* topoisomerase IA (EcTopoIA) inhibitors^4,5^. It was reported that PPEF binds to the acidic triad of the catalytic domain of EcTopoIA in nonlinear reaction kinetics^6^. DNA topoisomerases regulate DNA metabolic processes, including DNA replication, transcription, recombination, and chromosome condensation^7,8^. The ubiquitous distribution of DNA topoisomerase IA (TopoIA) renders it a potential bactericidal target capable of initiating cellular breakdown by either stabilizing or increasing the accumulation of the cleaved DNA complexes^9^. Several compounds like NSC76027^10^, fluoroquinophenoxazine^11^, Imipramine, Norclomipramine^12^, Polyamine 2471-12, Polyamine 2471-24^13^, Gold(III) macrocycle 10, Gold(III) macrocycle 10^14^ Seconeolitsine (SCN), *N*-methyl-seconeolitsine (N-SCN)^15^ were reported as small molecule inhibitors of bacterial TopoIA activity; however, none of them reached the clinical level.

In this study, we investigated the mechanism of two synthetic antibacterial molecules targeting TopoI of *S. aureus* (SaTopoIA)*, A. baumannii* (AbTopoIA), and *E. coli* (EcTopoIA and EcTopoIII), which are vital for DNA replication, thereby preventing the multiplication and dispersion of the bacteria. PPEF and another molecule 5-(4-butylpiperazin-1-yl)-2′-(3,4-dimethoxyphenyl)-1H,1′H-2,5′ bibenzo[d]imidazole (BPVF) are of notable interest due to their ability to inhibit the dual targets, TopoIA and TopoIII in *E. coli*. Herein, we establish a model wherein the gate opening of TopoIA is critical to the topoisomerase activity by analyzing the free energy of (1) TopoIA, (2) ssDNA/dsDNA bound TopoIA, (3) PPEF-bound TopoIA, and (4) ternary complex of TopoIA with ssDNA/dsDNA and PPEF. The free energy calculations suggest that the binding of the PPEF to topoI leads to compaction of the protein resulting in unfavorable DNA binding. PPEF and BPVF selectively inhibit the relaxation activity of SaTopoIA, AbTopoIA, EcTopoIA, and EcTopoIII. The time-dependent killing assay analysis confirmed that PPEF and BPVF have bactericidal activity and inhibit biofilm formation in *S. aureus*. The PPEF and BPVF-treated animals lacked any behavioral peculiarities or toxicity under observation for up to 30 days. We also demonstrated that PPEF and BPVF are highly efficacious against methicillin-resistant *S. aureus* (MRSA) infections in mouse septicemia models, with the added leverage of excellent potency against *E. coli* infection. Pharmacokinetics studies showed that these molecules are quickly absorbed through intravenous (i.v.) routes of administration with well-distributed and absorbed in BALB/c mice. To the best of our knowledge, no other existing antibiotics occupy this clinical niche. We believe that PPEF and BPVF are promising new drug candidates against WHO-priority pathogens. In this work, we showcase a successful development of a dynamic model of TopoIA, which can be used for screening novel TopoIA inhibitors.

## Results

### Effect of PPEF on DNA TopoIA gate open and closed dynamic states

**Fig. 1:**
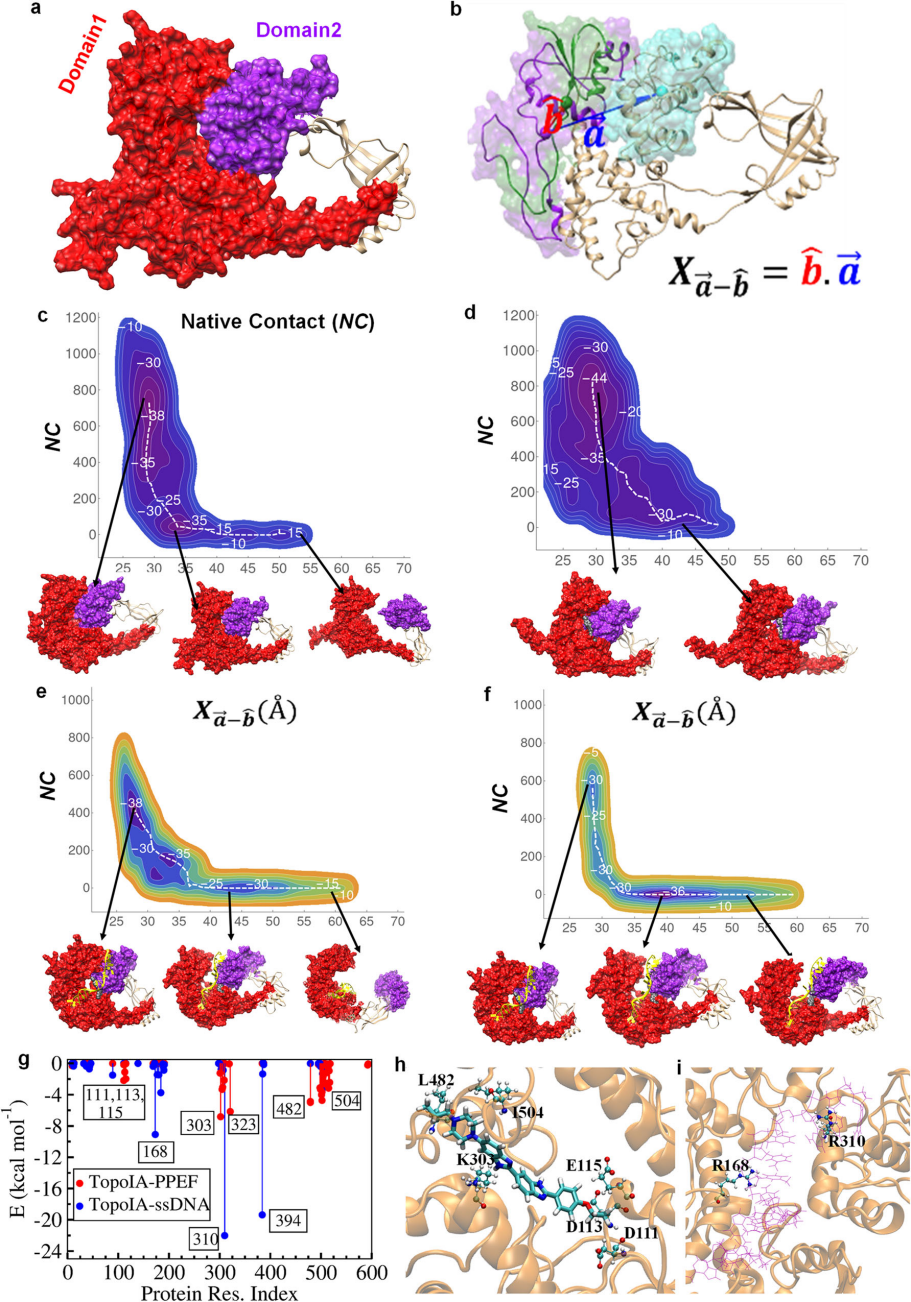
Reaction coordinate description and free energy surfaces of topoisomerase IA protein depicting gate opening and closing dynamics after binding with PPEF, ssDNA calculated by metadynamics. **a** The Native contact (**NC**) is used as a reaction coordinate between the domain1 (red color) and domain2 (purple color). The value native contact defines the total local interaction between domain1 and domain2 within a cut-off of 5 Å. **b** The vectoral distance ***X*** between domain1 and domain2 (see methods section for details). **c**–**f** The free energy surface represented the open-close dynamic for the following systems: **c** topoI; **d** ssDNA-TopoIA binary complex; **e** PPEF-TopoIA binary complex; **f** ssDNA-PPFE-TopoIA ternary complex. In each free energy surface, the X-axis is ***X*** (Å) and Y-axis is **NC**. The structures shown below each free energy surface panel represent a closed state [***X*** < 35 Å; **NC** ≥ 600], partial open [40 Å < ***X*** < 50 Å; **NC** < 100], and completely open [***X*** > 50 Å; **NC** ~0] states. **g** Interaction energies between various protein residues and PPEF, ssDNA, and dsDNA. **h** A pose of the ternary complex of topoIA protein: PPEF:DNA showing an interaction between a few key amino acids (L482, I504, K303, D111, D113, and E115) of protein with PPEF. **i** An orientation of topoisomerase IA: DNA complex showing interactions between R168 and R310 amino acids of protein with ssDNA.

TopoIA is known to exist in the closed (*P*_*c*_) and open (*P*_*o*_) conformations^16^ however, the energetics of these two conformational states are unknown. Additionally, the influence of the binding of various ligands, such as PPEF and dsDNA/ssDNA, on the dynamics of the two states is also unknown. Steered molecular dynamics (MD) simulations is used to indicate that the open state of the protein is characterized by the movement of the domain2 region relative to the rest of the protein^16^ domain1 (Fig. 1a). MD simulations using amber force fields and TIP3P water model (Supplementary Table 1) were carried out using Gromacs 2020^17^. Multiple 100 ns MD simulations of the protein does not sample the open state, which suggests that the barrier for the opening is high and is not accessible within 100 ns simulation time as indicated by the RMSD (Supplementary Fig. 2). Therefore, to probe the dynamics of the opening up of the TopoIA, well-tempered metadynamics simulations were carried using two reaction coordinate descriptors mentioned in the Eqs. 1 and 2, (i) vectoral distance ***X*** (Fig. 1b) and (ii) pairwise contacts (**NC**). The reaction coordinate ***X*** represents the vectoral distance between the center-of-mass of left most segments of domain1 and domain2 (Fig. 1b). The increase in the value of ***X*** relative to the native conformation indicates the (gate) opening up the protein. The reaction coordinate **NC** represents the number of pairwise heavy-atom contacts within a cut-off distance of 5.5 Å between domain1 and domain2. The ***X*** and **NC** values of TopoIA in its native state are about 27 and 700, respectively. The increases in the value of ***X*** and the lowering of the **NC** value suggests the loss of interaction between the two domains and is interpreted as (gate) opening of the protein. In the unrestrained and unbiased MD simulations, the ***X*** and **NC** values of all the systems in consideration do not change appreciably to reflect the (gate) opening dynamics (Supplementary Fig. 3). The free energy surfaces were calculated from the metadynamics simulations along the two reaction coordinate descriptors ***X*** and **NC** (Fig. 1c–f), in which the contours represent the isoenergetic surfaces while the white dashed line indicates the minimum free energy path for the gate opening process. The gate open-close configurational dynamics of the TopoIA shows the presence of three prominent minima along the minimum free energy path, which correspond to (i) closed state [***X*** < 35 Å; **NC** ≥ 600], (ii) partial open state [40 Å < ***X*** < 50 Å; **NC** < 100], and (iii) completely open state [***X*** > 50 Å; **NC** ~ 0]. The absolute free energies of the closed and open states of TopoIA were about –38 and –15 kcal mol^–1^, respectively (Fig. 1c and Supplementary Movie 1), with a free energy difference (ΔG) of about 23 kcal mol^–1^, which suggest that TopoIA exists exclusively in the closed state, which is in agreement with an earlier report^18^. The free energy surface of TopoIA in the presence of ssDNA (Fig. 1d and Supplementary Movie 2) reveals that the value of **NC** is lowered from about 700 to about 400 with marginal changes in the value of ***X***. The lower value of native contacts (***NC***) indicates that the binding of ssDNA modifies the interface between domain1 and domain2, while marginal changes in the value of ***X*** indicates that the variation in the global structure of the protein is insubstantial. These results suggest that the binding of the ssDNA interrupts the local interaction between the two domains without making any large segmental motions of the domains (Fig. 1g, h). The binding of ssDNA to TopoIA is energy neutral, which can be rationalized by the formation of new ssDNA-TopoIA contacts in lieu of contacts between the two domains of TopoIA. The binding of PPEF to TopoIA in the closed state results in a free energy value of –44 kcal mol^–1^, with a net stabilization of about –6 kcal mol^–1^ relative to the free protein, as shown in Fig. 1e (Supplementary Movie 3). The RMSD plots, along with the minimum free energy path (Supplementary Fig. 4), indicate that the opening of TopoIA results in an RMSD of about 20 Å, while binding of PPEF leads to rigidification and consequent RMSD of 8 Å. The free energy surface of ssDNA in the presence of PPEF-bound TopoIA indicates a thermodynamically unstable binding of ssDNA to the protein by at least 8 kcal mol^−1^ (Fig. 1f and Supplementary Movie 4). Similarly, the simulations reveal that the binding of dsDNA to TopoIA destabilizes the closed state in favor of the partially open state by about 5 and 15 kcal mol^–1^, in the absence and presence of PPEF, respectively (Supplementary Fig. 5a, b and Supplementary Movies 5, 6). Furthermore, the unbinding free energy calculations (Supplementary Fig. 6) also suggest that the unbinding of the ssDNA to TopoIA in the presence of PPEF is favorable by 5.7 kcal mol^–1^ than in the absence. These results are in accord with experimental findings that PPEF acts as an inhibitor of TopoIA.

### WHO-priority pathogens resistant to all frontline antibiotics are sensitive to PPEF and BPVF

**Fig. 2:**
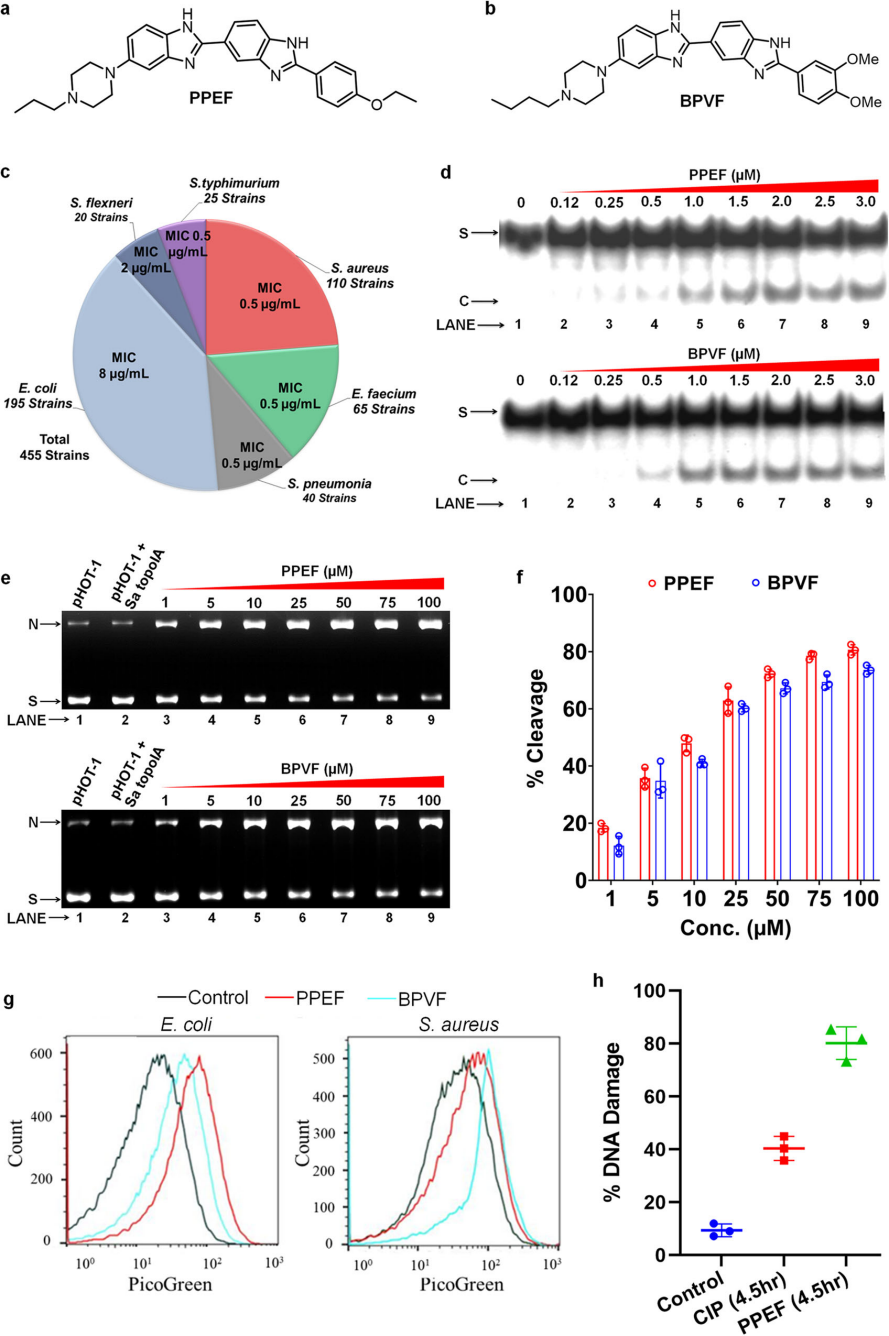
PPEF and BPVF show bactericidal activities against 08 priority pathogens listed by WHO in 2020 as multidrug-resistant bacteria. **a**, **b** The structure of PPEF and BPVF. **c** The MIC_90_ of PPEF is shown in the pie chart against ~455 MDR bacterial pathogens. **d** PPEF and BPVF inhibit religation of5′-^32^P-labeled ssDNA by SaTopoIA. Lane 1: 5′-^32^P-labeled ssDNA; Lane 2:9 5′-^32^P-labeled ssDNA with SaTopoIA in the presence of 0.12, 0.25, 0.5, 1.0, 1.5, 2.0, 2.5, and 3.0 µM compound, respectively. Analysis of products formed from ^32^P-labeled ssDNA. **e** PPEF and BPVF effect on SaTopoIA mediated cleavage of pHOT-1 plasmid DNA and percentage cleavage of plasmid DNA. Lane 1: pHOT-1 plasmid DNA; Lane 2: Cleavage of plasmid DNA by topoI; Lane 3:9 Increase in cleavage of plasmid DNA by TopoIA in the presence of 1, 5, 10, 25, 50, 75, and 100 μM PPEF or BPVF. **f** Representative graph of percentage cleavage inhibition of SaTopoIA against PPEF and BPVF. **g** A comparison of fluorescence intensity of Picogreen between the total number of untreated cells (black) and after treatment with either BPVF (blue) or PPEF (red), respectively. Data were presented as mean ± SD of three independent experiments. **h** %TUNEL positive *E. coli* cells (mean ± SD at 4.5 h post-treatment) upon PPEF and Ciprofloxacin treatment at 4.5 h. Values reflect the mean percentage of a treated population. Data were presented as mean ± SD of three independent experiments.

PPEF and BPVF (Fig. 2a, b) were assessed for their antibacterial activity against pathogens by determining the minimum inhibitory concentration (MIC) and minimum bactericidal concentration (MBC), following the 2020 guidelines of the Clinical and Laboratory Standards Institute (CLSI)^19^. The Epsilometer test (E-test)^20^ was used for determining the MIC of standard antibiotics (Table 1). The potent antimicrobial action of PPEF and BPVF against methicillin-sensitive *S. aureus* (MSSA), MRSA, and vancomycin-resistant *S. aureus* (VRSA) is reflected by MIC and MBC that ranged from 0.25–4 µg/mL (0.4–6.4 µM) and 0.25–16 µg/mL (0.4–25.6 µM), respectively (Table 1). The fluoroquinolone-resistant *S. flexneri* and *S. typhimurium* exhibited high sensitivity to PPEF and BPVF (MIC range: 0.25–4 µg/mL (0.4–6.4 µM); MBC range: 0.25–8 µg/Ml (0.4–12.9 µM) (Table 1). The lower MIC levels of PPEF and BPVF of the above two pathogens can be attributed to 98% similarity in sequence and structure of type IA topoisomerases of *S. flexneri* and *S. typhimurium* with *E. coli* (Supplementary Fig. 7a, b), as all three belong to the family *Enterobacteriaceae*. The PPEF and BPVF were also effective against carbapenem-resistant *Enterobacteriaceae*, ESBL-producing *Enterococcus faecium*, penicillin-non-susceptible *Salmonella* spp. with MIC of 0.25–4 µg/mL (0.4–6.4 µM). PPEF and BPVF showed MIC of 0.25–8 µg/mL (0.4–12.8 µM) against carbapenem-resistant *A. baumannii* strains. Both compounds exhibited potent activity against ESBL-producing *E. coli* (Table 1) water-borne and Urinary tract infection (UTI) causing *E. coli* multidrug-resistant (MDR) strains resistant to β-lactam antibiotics with MIC in the range of 2–4 µg/mL (3.2–6.4 µM) (supplementary table 2). The MBC of maximum bacterial strains was either similar to the MIC or one-fold higher than the MIC. PPEF and BPVF showed MIC in the range of 0.25–8 µg/mL(0.4–12.8 µM) (Supplementary Table 3) against a few mutant and knockout strains of *E.coli*, e.g., *ΔacrA, ΔemrA, ΔtolC, ΔompC, ΔompF, ΔtopA, ΔtopB, ΔyciM, ΔlpxC*, and *ΔlpxD*. The clinical *E. coli* strains (EC118, EC49HSV, EC555, and EC284LF), which are resistant to nalidixic acid, as well as ciprofloxacin (CIP) with mutated gyrase (S83L, D87N) and topoIV (S80I, E84V, S485A, and E460D) were susceptible to PPEF and BPVF (Supplementary Table 4), suggesting that these molecules did not target gyrase^21^. PPEF and BPVF killed non-virulent *M. tuberculosis* (H37Ra) and virulent *M. tuberculosis* (H37Rv) bacteria at 8 and 16 µg/mL (12.8 and 25.6 µM), respectively. The hydrophobic propyl and butyl chains at the piperazine and methoxy and ethoxy groups at the phenyl ring are optimal to show effective pathogen-killing activity. Both compounds engender antibacterial activity levels by successfully breaching the complex cell wall of *M. tuberculosis*. The details of the MIC of PPEF against 455 clinical and laboratory strains are shown in the pie chart (Fig. 2c and Table 1). Taken together, we found that PPEF and BPVF are bactericidal strains that are resistant to the majority of standard antibiotics.

**Table 1:** Broad spectrum activity of PPEF, BPVF, and standard antibiotics against WHO-priority pathogens bacterial strains.

No.	Strains	PPEF (µg/mL) MIC	PPEF (µg/mL) MBC	BPVF (µg/mL) MIC	BPVF (µg/mL) MBC
1.	MSSA (ATCC 25923)	0.5 ± 0.02	0.25	0.5 ± 0.07	0.5
2.	MSSA (O-182)	1.0 ± 0.04	2	4.0 ± 0.03	8
3.	MSSA (O-168)	1.0 ± 0.07	1	4.0 ± 0.02	4
4.	MSSA (O-193)	0.5 ± 0.01	0.5	2.0 ± 0.04	4
5.	MSSA (O-194)	1.0 ± 0.04	1	4.0 ± 0.03	4
6.	MSSA (O-198)	1.0 ± 0.07	1	4.0 ± 0.02	8
7.	MSSA (W-292)	0.5 ± 03	1	4.0 ± 0.04	8
8.	MSSA (W-294)	0.5 ± 0.02	1	4.0 ± 0.02	4
9.	MSSA (W-295)	1.0 ± 05	1	4.0 ± 0.07	8
10.	MSSA (W-311)	1.0 ± 0.07	1	4.0 ± 0.01	4
11.	MRSA (ATCC 43300)	0.5 ± 0.04	0.5	0.5 ± 0.02	1
12.	MRSA (O-49)*	0.5 ± 0.02	1	1.0 ± 0.04	4
13.	MRSA (O-38) *	0.5 ± 0.08	1	1.0 ± 0.03	4
14.	MRSA (O-5)*	0.25 ± 0.02	0.25	0.25 ± 0.04	0.5
15.	MRSA (W-7)*	0.5 ± 0.08	1	2.0 ± 0.08	4
16.	MRSA (O-23)*	0.5 ± 0.04	1	2.0 ± 0.08	4
17.	VRSA (S976)*	0.25 ± 0.08	0.25	0.25 ± 0.04	0.5
18.	VRSA (S1016)*	0.25 ± 0.05	0.25	0.25 ± 0.02	0.5
19.	VRSA (S982)*	0.5 ± 0.02	0.5	1.0 ± 0.03	2
20.	MRSA (W-295)*	0.5 ± 0.03	1	1.0 ± 0.03	4
21.	MRSA (292)*	0.5 ± 0.02	1	2.0 ± 0.02	4
22.	MRSA (O-182)*	0.5 ± 0.05	1	1.0 ± 0.05	4
23.	MRSA (O194)*	0.5 ± 0.04	1	1.0 ± 0.07	4
24.	*Shigella flexneri* (MTCC 1457)	0.5 ± 0.01	0.5	0.25 ± 0.04	1
25.	*Shigella flexneri* (W31 *)	0.25 ± 0.03	0.25	0.25 ± 0.05	0.5
26.	*Shigella flexneri* (W-67*)	0.25 ± 0.02	0.25	0.25 ± 0.02	0.5
27.	*Salmonella Typhi* (MTCC 1254)	0.25 ± 0.04	0.25	0.25 ± 0.07	0.25
28.	*Salmonella Typhi* (St 412*)	0.25 ± 0.05	0.25	0.25 ± 0.04	0.25
29.	*Salmonella Typhi* (W-561*)	1.0 ± 0.02	2	2.0 ± 0.05	8
30.	*Salmonella Typhi* (W-653*)	0.5 ± 0.03	1	1.0 ± 0.03	4
31.	*Enterococcus faecium* (MCC 2105)	0.5 ± 0.01	2	1.0 ± 0.07	8
32.	*Enterococcus faecium* (Ent1121*)	0.25 ± 0.03	0.25	0.25 ± 0.02	0.5
33.	*Enterococcus faecium* (Ent1150*)	0.25 ± 0.02	0.25	0.25 ± 0.09	0.25
34.	*Enterococcus faecium* (Ent1376*)	0.5 ± 0.03	1	1.0 ± 0.07	4
35.	*Enterococcus faecium* (Ent439*)	0.5 ± 0.04	1	1.0 ± 0.06	4
36.	*Enterobacter hormaechei* (MCC 2289)	0.5 ± 0.02	1	2.0 ± 0.04	4
37.	*Enterobacter hormaechei* (E34*)	0.5 ± 0.08	1	1.0 ± 0.04	4
38.	*Enterobacter hormaechei* (E589*)	0.5 ± 0.02	1	2.0 ± 0.05	4
39.	*Klebsiella pneumonia* (MTCC 2272)	0.5 ± 0.04	1	1.0 ± 0.09	4
40.	*Klebsiella pneumonia* (K531*)	0.5 ± 0.06	0.5	1.0 ± 0.06	2
41.	*Klebsiella pneumonia* (K589*)	1.0 ± 0.06	2	1.0 ± 0.02	8
42.	*Acinetobacter baumannii* (W-179*)	2.0 ± 0.05	8	4.0 ± 0.05	16
43.	*Acinetobacter baumannii* (W-704*)	2.0 ± 0.02	4	4.0 ± 0.01	32
44.	*Acinetobacter baumannii* (Ab-159*)	2.0 ± 0.04	8	4.0 ± 0.02	16
45.	*Acinetobacter baumannii* (Ab-158*)	2.0 ± 0.04	8	4.0 ± 0.07	8
46.	ESBL producing *E.coli* (NCTC11954)	20 ± 0.02	4	8.0 ±0.01	16
47.	ESBL producing *E.coli* (NCTC13351)	1.0 ± 0.04	2	4.0 ± 0.05	8
48.	ESBL producing *E.coli* (NCTC13400)	2 ± 0.05	4	8.0 ± 0.01	16
49.	ESBL producing *E.coli* (NCTC13352)	0.5 ± 0.06	1	2.0 ± 0.02	4
50.	ESBL producing *E.coli* (NCTC13451)	0.5 ± 0.07	8	4.0 ± 0.04	32
51.	ESBL producing *E.coli* (NCTC13461)	2.0 ± 0.02	2	8.0 ± 0.06	16
52.	ESBL producing *E.coli* (NCTC13462)	0.5 ± 0.01	8	2.0 ± 0.02	8
53.	ESBL producing *E.coli* (NCTC13476)	2.0 ± 0.01	4	8.0 ± 0.01	16
54.	ESBL producing *E.coli* (NCTC13846)	2.0 ± 0.01	8	8.0 ± 0.02	16
55.	ESBL producing *E.coli* (NCTC13919)	1.0 ± 0.06	2	4.0 ± 0.05	8

MIC of PPEF against ~455 bacterial strains are given in Supplementary Data 2 excel sheet.

Resistance profile of clinical and standard bacterial strains against a panel of antibiotics: MIC of PPEF and BPVF against Methicillin resistance *S. aureus*, [(*ATCC43300*)-AMP, TSH, FOX, OFX, CLI, TET, CIP, CL, GEN, CTK, KAN), (O5-AMP, TSH, FOX, OFX, CLI, TET, CIP, GEN, CTK, KAN),(*O23*-FOX, OFX, Cl, CTK, KAN), (*O49*-TSH, CL, GEN), (O38-AMP, TSH, FOX, OFX, TET, CIP, CL, CHL, GEN, CTK, KAN), (*O182*- CLI, TET, CIP, GEN, CTK, KAN), (*O194*-AMP, TSH, OFX, GEN, CTK, KAN), (W7- TSH, FOX, OFX, CIP, CL, CTK, KAN), (*W292*-AMP, TSH, FOX, OFX, CIP, CL, CTK, KAN), (*W295*-AMP, TSH, OFX, CLI, TET, CIP, CL, GEN, KAN), vancomycin resistance *S. aureus*(VRSA), (ST976-AMP, TSH, FOX, OFX, CLI, TET, CIP, CHL, GEN, CTK, KAN), *ST982*-AMP, TSH, FOX, OFX, CLI, TET, CIP, CHL, GEN, CTK, KAN), (*ST1016*- TSH, FOX, OFX, CIP, CL, CTK, KAN), *Shigella* spp. (*MTCC1457*-OFX, CLI, TET, CIP, CL),(*W66*- CIP, CL, CHL, GEN, CTK, KAN), (*W67*-AMP, TSH, OFX, CLI, TET, CHL, CTK, KAN), *Salmonella* spp. (*MTCC1254*-CLI), (St412-OFX, CLI, CIP), (W561-CLI),(W653-OFX, CLI, CIP), *Enterococcus faecium* (*MCC2105*, *Ent439/1121/1150/1376*), *Enterobacteriaceae* Spp. (*MCC2289*-AMP, FOX, CLI), (*E34*- AMP, TSH, FOX, OFX, CLI, TET, CHL, GEN, CTK), (*E589*-TSH, FOX, OFX, CLI, TET, CIP, CL, CHL, KAN), *Klebsiella* spp. (*MTCC2272*-AMP, TSH, FOX, CL, CHL, GEN, CTK, KAN), (*K531*-AMP, TSH, FOX, OFX, CLI, TET, CIP, CHL, GEN, CTK, KAN), (*K589*-AMP, TSH, FOX, OFX, CLI, TET, CIP, CL, CHL, GEN, CTK, KAN), *A. baumannii* (*W179*-AMP, TSH, FOX, OFX, TET CIP, CHL, GEN, CTK, KAN),(*W704*-AMP, TSH, FOX, OFX, TET, CIP CHL, GEN, CTK, KAN), (*Ab158*-AMP, TSH, FOX, OFX, CLI, TET, CIP, CL, CHL, GEN, CTK, KAN), (*Ab159*-AMP, TSH, FOX, OFX, CLI, TET, CIP, CL, CHL, GEN, CTK, KAN), ESBL producing *E.coli* from NCTC.

Standard antibiotics—Amp: ampicillin, TSH-Co: trimoxazole, FOX: cefoxitin, OFX: ofloxacin, CLI: clindamycin, TET: tetracycline, CIP: ciprofloxacin, CL: colistin, CHL: chloramphenicol, GEN: gentamicin, CTK: cefotaxime, KAN: kanamycin). MIC data were given as mean ± SD for four replicates and are representative of three independent experiments.

*Represents MDR-resistant strains.

### PPEF and BPVF increase ssDNA cleavage by TopoI and TopoIII

PPEF and BPVF showed increased accumulation of cleaved ssDNA, causing the death of bacterial cells (Fig. 2d). The Mg^2+^ is required for DNA religation and completes the catalytic cycle of TopoIA; however, Mg^2+^ is not necessary for DNA cleavage^4^. After initial DNA cleavage by *S. aureus* TopoIA, the addition of Mg^2+^ allows ssDNA–religation to be re-established with a low level of DNA product remnants (Supplementary Fig. 8d). Presently, PPEF and BPVF led to topoI-DNA complexes with 58 bp oligonucleotide DNA substrate resulting in an increase in the accumulation of a cleaved 20 bp product (Fig. 2d) at 0.25 and 0.5 µM respectively using SaTopoIA. Also, PPEF and BPVF showed 50% cleavage of plasmid DNA at 12.51 and 16.86 µM respectively (Fig. 2e, f). These results suggested that PPEF and BPVF reversibly stabilized the topoI-DNA complex by inhibiting the DNA religation process in a dose-dependent manner. PPEF and BPVF bind at the interface of protein and DNA, analogous to clerocidin (CL), which inhibits bacterial DNA gyrase and mammalian DNA topoisomerase II as an interfacial inhibitor^22^. Thus, we could conclude that benzimidazole binds to the DNA:EcTopoIA complex in an irreversible manner and act as a topoisomerase inhibitor. We observed that EcTopoIII showed increased nicked DNA product with increasing concentrations of PPEF and BPVF (Supplementary Fig. 8a, b). EcTopoIII is likely involved in disentangling partially single-stranded intermediates formed during replication, repair, or recombination. PPEF and BPVF inhibit type I topoisomerases, making them more effective against both Gram-positive and Gram-negative bacteria.

### Flow cytometry-based Picogreen assay suggests PPEF and BPVF promote DNA fragmentation and condensation

DNA fragmentation and chromosomal condensation lead to physiological changes that induce cell cycle arrest and delayed repair pathways resulting in bacterial cell death^23^. Flow cytometry-based picogreen assay quantifies the amount of DNA in PPEF and BPVF-treated bacterial cells. The increased peak area and peak shift was observed in PPEF and BPVF-treated *S. aureus* and *E. coli* cells compared to untreated cells indicating increased cell size filamentation (Fig. 2g). To determine whether death-inducing stress by PPEF were capable of promoting DNA fragmentation of the *E. coli* chromosome, we performed terminal deoxynucleotidyl transferase dUTP nick-end labeling (TUNEL) on PPEF-treated cells and observed weak TUNEL staining in cells with no treatment (10%). In contrast, treatment with PPEF and CIP yielded 80% and 40% of TUNEL-positive cells (Fig. 2h). The data indicate that fragmentation of *E. coli* DNA occurs upon treatment with cell death-inducing agents.

### PPEF and BPVF inhibit the relaxation activity of bacterial TopoIA and TopoIII

**Fig. 3:**
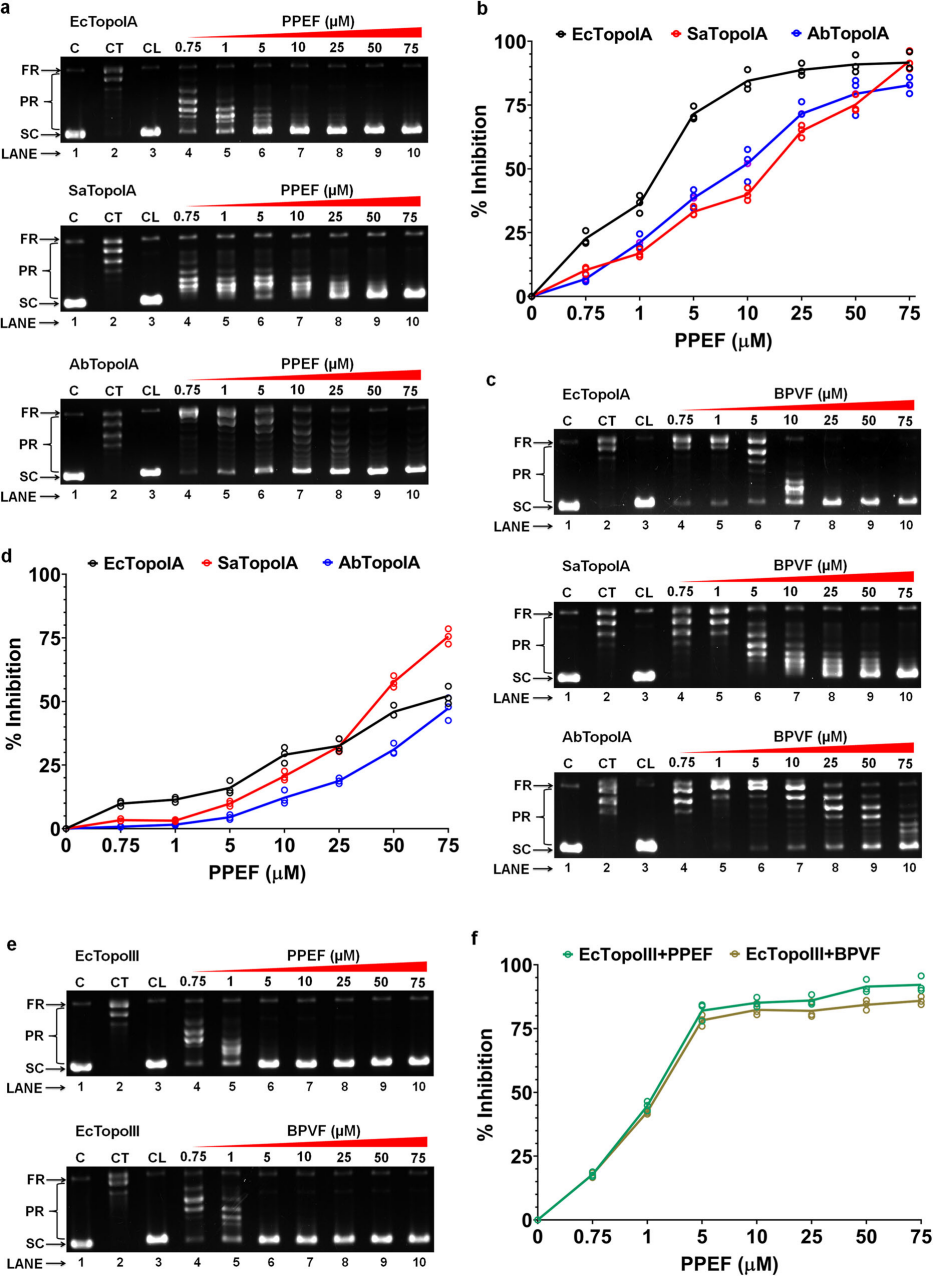
Selective inhibition of type IA topoisomerase by PPEF and BPVF without EtBr. **a**, **b** Inhibition of relaxation activity of EcTopoIA, SaTopoIA, AbTopoIA, and calculation of percentage inhibition in the presence of PPEF. Lane 1: pHOT-1 plasmid DNA; Lane 2: Relaxation of plasmid DNA by TopoIA; Lane 3: pHOT-1 plasmid DNA with 75 μM PPEF; Lanes 4–9: Inhibition of relaxation of plasmid DNA by TopoIA in the presence of 0.75, 1, 5, 10, 25, 50, and 75 μM PPEF. **c**, **d** Inhibition of relaxation activity of EcTopoIA, SaTopoIA, AbTopoIA, and percentage inhibition in the presence of BPVF. Lane 1: pHOT-1 plasmid DNA; Lane 2: Relaxation of plasmid DNA by topoIA; Lane 3: pHOT-1 plasmid DNA with 75 μM BPVF; Lanes 4−9: Inhibition of relaxation of plasmid DNA by topoIA in the presence of 0.75, 1, 5, 10, 25, 50, and 75 μM BPVF. **e**, **f** Inhibition of relaxation activity of EcTopoIII and percentage inhibition in the presence of PPEF and BPVF. Lane 1: pHOT-1 plasmid DNA; Lane 2: Relaxation of plasmid DNA by TopoIII; Lane 3: pHOT-1 plasmid DNA with 75 μM compound; Lanes 4–9: Inhibition of relaxation of plasmid DNA by TopoIII in the presence of 0.75, 1, 5, 10, 25, 50, and 75 μM compound. All panels show representative results for an experiment repeated at least three times. FR fully relaxed DNA, PR partially relaxed DNA, S supercoiled DNA, C control, CT control + topoisomerase enzyme, CL control + compound.

Bisbenzimidazole has an advantage over other prospective antibiotics because it does not act upon gyrase. This increases the possibility of using PPEF and BPVF against gyrase mutant bacterial strains exposed to prolonged fluoroquinolone. We cloned SaTopoIA, AbTopoIA, and EcTopoIII genes (Supplementary Fig. 9a–d) and protein was purified using Ni-NTA column chromatography (Supplementary Fig. 10a–c) for further assays. The IC_50_ values of PPEF to inhibit the DNA relaxation activity of EcTopoIA, SaTopoIA, and AbTopoIA were 2.71, 15.50, and 9.48 µM (Fig. 3a, b), respectively. The IC_50_ values of BPVF to inhibit the DNA relaxation activity of EcTopoIA, SaTopoIA, and AbTopoIA were >50, 41.4, and >50 µM (Fig. 3c, d), respectively. The IC_50_ values of PPEF and BPVF to inhibit the DNA relaxation activity of EcTopoIII were 1.58 and 1.96 µM (Fig. 3e, f), respectively. All the above relaxation assays were performed without EtBr, the EtBr was added later in the gel for visualization. The relaxation assay using EtBr was also performed to rule out an effect of EtBr on the enzymatic activity of TopoIA enzyme in the presence of PPEF using literature method^24^, as we did not observe any relaxation activity in the presence of EtBr with or without PPEF (Supplementary Fig. 9). Further, we have checked the effect of PPEF and BPVF with increasing concentration from 1–100 µM on the gyrase enzyme of *S. aureus* (SaGyrase) (Supplementary Fig. 10d, e) and human topoisomerase I (Supplementary Fig. 10f, g). We did not observe any inhibition on the activity of gyrase and human topoI. PPEF and BPVF kill several MDR strains, which are resistant to fluoroquinolones due to acquired mutations in gyrase. Similarly, as these molecules do not inhibit human topoisomerase I, hence will be safer as compared to other topoisomerase inhibitors.

### PPEF and BPVF cause severe bacterial cell filamentation

**Fig. 4:**
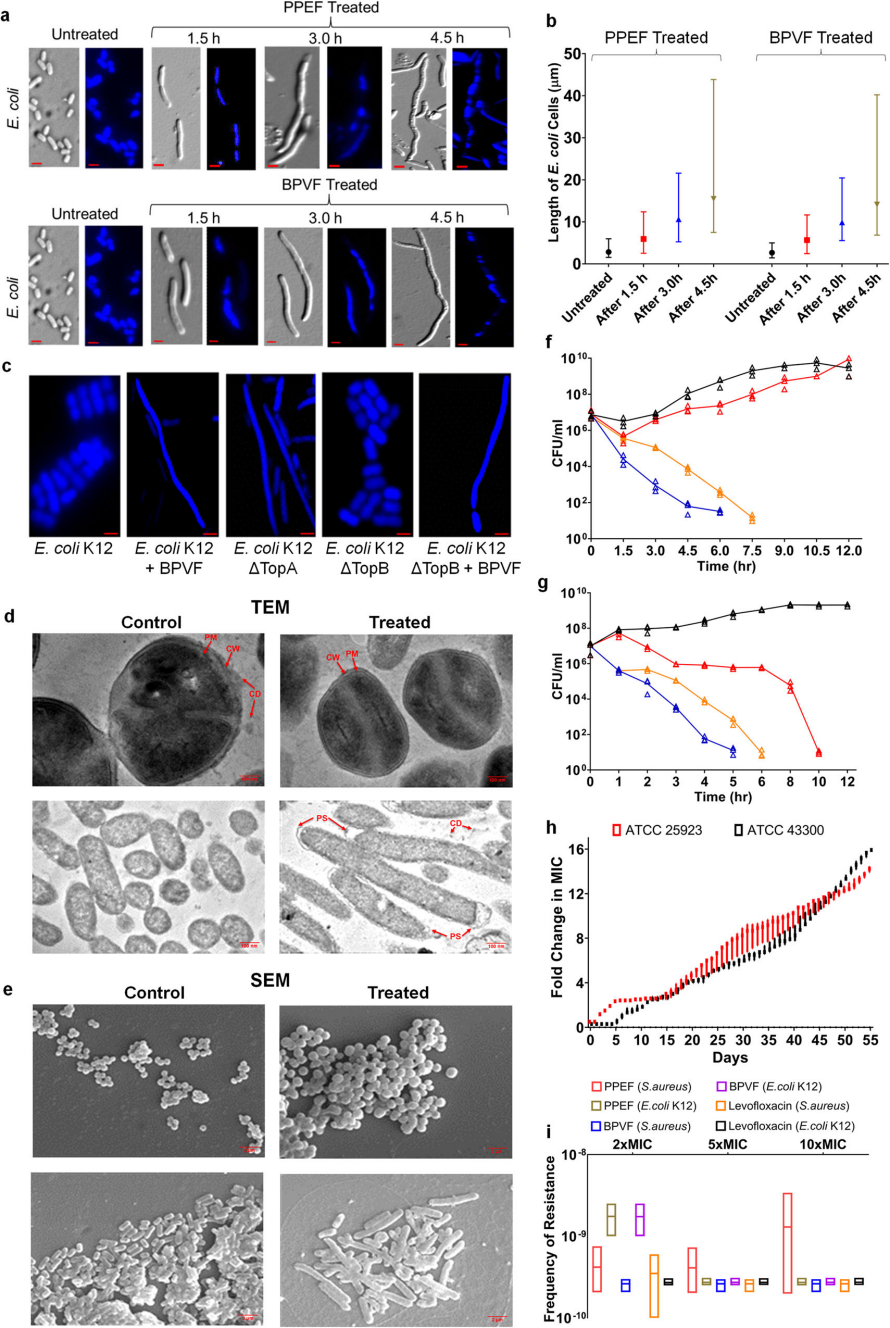
PPEF and BPVF cause bacterial cell filamentation but does not induce any mutation in bacteria, even at 10XMIC. **a** Effect of PPEF and BPVF treatment on the cellular morphology of *E. coli* K12. Panels show representative bright-field, and fluorescent micrographs of *E. coli* K12 cells treated with PPEF and BPVF for 1.5, 3, and 4.5 h, respectively. Scale bars indicate 5 μm. **b** The calculated length of *E. coli* treated cells at 1.5, 3, and 4.5 h. **c** Changes in the cell morphology after BPVF treatment to *E. coli K12*ΔTopoI, ΔTopoIII, and wild-type *E. coli*. Cell morphology changes were recorded after BPVF treatment to *E. coli K12 *ΔTopoI, ΔTopoIII, and wild-type *E. coli*. Scale bars indicate 5 μm. **d**, **e** Cell wall and membrane alterations of *S*. *aureus* (ATCC43300) and *E*. *coli* K12, suggested by typical and representative TEM and SEM micrographs after treatment for 2 h at 37 ˚C with BPVF at their MICs. The respective scale bar for TEM and SEM images is 100 nm and 2 µm. Untreated bacteria cell walls are unaltered, and membranes remain intact. Inset shows damaged cell wall (CW), plasma membrane (PM), increased periplasmic space (PS), and the expulsion of cell debris (CD). **f** Time-killing assay of *S. aureus* without treatment (black), after CIP treatment (red), BPVF treatment (orange), and PPEF treatment (blue). The error bars represent the SEM for three independent experiments. **g** Time-killing assay of *E. coli* K12 without treatment (black), or treatment with either CIP (red), BPVF (orange), or PPEF (blue). The error bars represent SEM for three independent experiments. **h** Resistance acquisition during serial passaging in the presence of sub-MIC levels of antimicrobials. The y-axis is the highest concentration of the cells that grew during passaging. For ATCC 25923, MIC was 28-fold increases and for ATCC 43300 (MRSA), MIC was 32-fold increases from the initial MIC of PPEF. The figure is representative of three independent experiments. **i** Frequency of resistance with and without PPEF, BPVF, or levofloxacin. The error bars represent SEM for three independent experiments.

The chromosomal topology and bacterial cell division experiment through microscopy suggests that PPEF and BPVF treatment leads to membrane deformation and detachment, with internal lateral expansion and stacks in bacterial cells (Fig. 4a, b), as both targets TopoIA in bacteria. Cell filamentation corroborates that both the compounds compromises the cell morphology of bacteria and inhibit topoIA and TopoIII enzyme, evidenced by the bacterial morphology of ΔEcTopoIA (ΔtopA) *E. coli* K12 and ΔEcTopoIII (ΔtopB) strains (Fig. 4c).

Morphological abnormalities induced by BPVF in *E. coli* (MG1655) and *S. aureus* (ATCC 43300) were confirmed by transmission electron microscopy (TEM) and scanning electron microscopy (SEM), respectively (Fig. 4d, e). SEM images showed that the bacterial cell wall and membrane were completely impaired in BPVF-treated *S. aureus and E. coli* cells. In conjunction with partial disruption of the cell wall and cytoplasmic membrane, pore formation resulted in the outflow of cell contents and the complete collapse in certain cells. TEM images showed cell debris (CD) outside the cell surface, increased cell size, increased periplasmic space, cell deformation, as well as cell wall (CW) and cell membrane (CM) disruption in treated *S. aureus*. Interestingly, the cell wall of *S. aureus* was partially damaged without cell membrane disruption.

The time-dependent killing was conducted with 1×MIC PPEF (0.4 and 12.8 µM) and BPVF (1.6 and 25.4 µM) to check the inhibition kinetics of *S. aureus* and *E. coli*. The BPVF and PPEF-treated MRSA (ATCC 43300) strain showed a (3×log_10_)-fold decrease in colony-forming units (CFU) after 4.5 and 3 h, respectively, whereas CIP failed to kill the MRSA strain (Fig. 4f). The *E. coli* cells were decreased by (3 log_10_)-fold CFU after 3 and 4.5 h of treatment with PPEF and BPVF, respectively, whereas (3 log_10_)-fold CFU with the treatment of CIP achieved after 9 h (Fig. 4g).

### Limited resistance of bacteria against PPEF and BPVF

We treated three independent isogenic populations of *S. aureus* ATCC 25923 (wild-type) and ATCC 43300 (MRSA) (2 × 10^6^) with 1 × MIC PPEF (0.5 µM) in broth culture up to ~440 generations (55 days), the MIC of PPEF gradually increased by 28 and 32 folds (Fig. 4h) respectively, suggesting that PPEF induces less resistance in bacterial cells.

A spontaneous mutation is generally induced by antibiotics with a single molecular target. We determined the spontaneous frequency of resistance against PPEF, BPVF, and levofloxacin in *S. aureus* and *E. coli*. We exposed 10^10^ bacterial cells of *E. coli* MG1655 and *S. aureus* ATCC 43300 (MRSA) with 2-, 5-, and 10×MIC of PPEF, BPVF, and levofloxacin, respectively (Fig. 4i). No resistant variants of *S. aureus* were detected at 2-, 5-, and 10×MICs of either PPEF or BPVF. However, in levofloxacin, colonies were observed at 2 × MIC PPEF. Similarly, there were no resistant variants of *E. coli* detected when bacterial cells were exposed to 5- and 10×MIC of PPEF, and 2-, 5-, and 10×MIC of levofloxacin. As PPEF exerts excellent dual-target enzyme inhibition in bacteria, the low frequency of resistance in bacteria is an added advantage. Notably, no PPEF-resistant mutants emerged when 2 × 10^10^ MRSA cells were exposed to 2 × MIC of PPEF, giving the calculated frequency of resistance 2.2 × 10^−10^ and <10^−10^ in *E. coli* and *S. aureus* to PPEF.

### Accumulation and Efflux of PPEF and BPVF in bacterial Cell

**Fig. 5:**
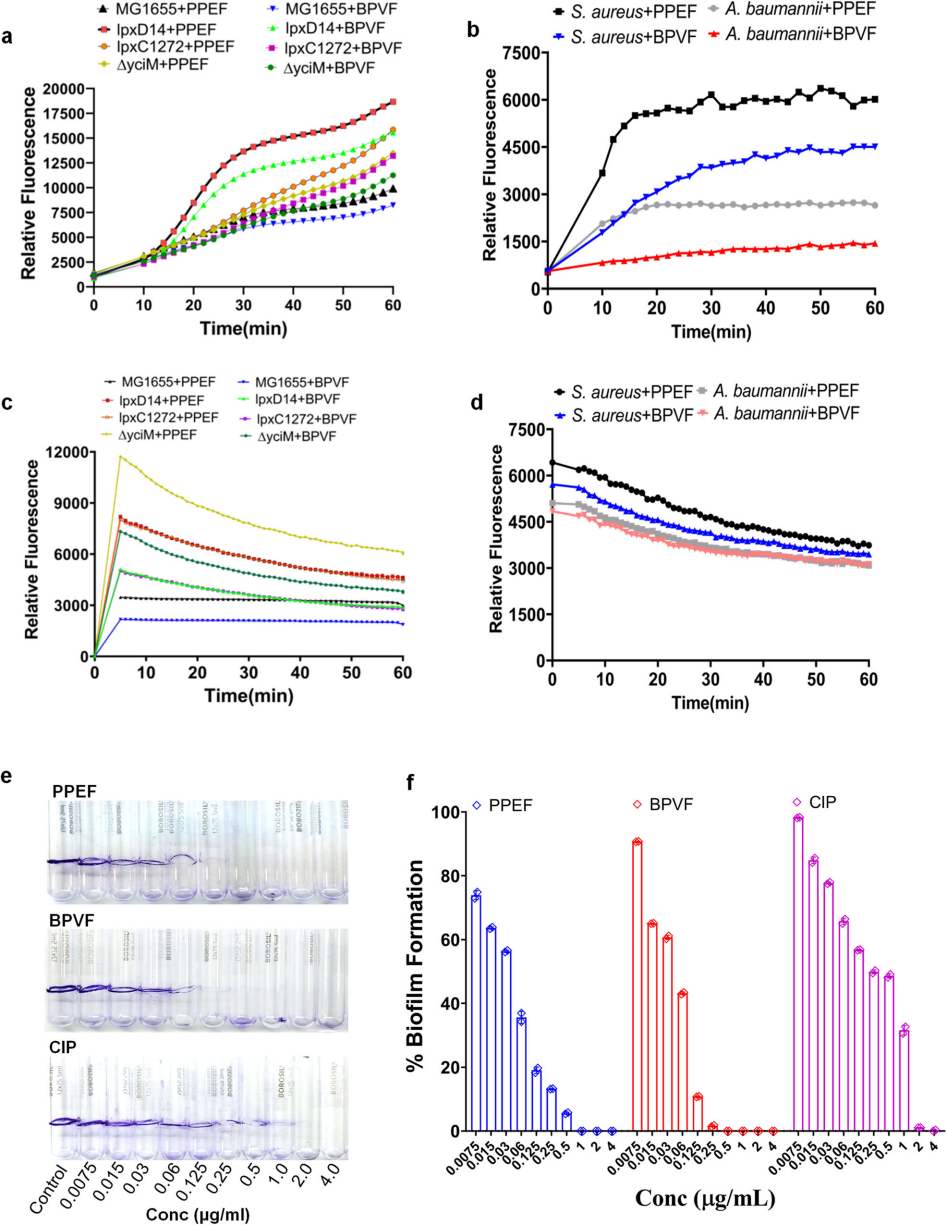
PPEF or BPVF show fast accumulation and inhibition of biofilm formation. **a** The accumulation assay of PPEF or BPVF in *E. coli* K12 (MG1655), *ΔyciM* (MG1655)*, lpxC* (MG1655), and *lpxD* (MG1655) strains. Data were presented as the mean of three independent experiments with four replicates. **b** The accumulation assay graph of PPEF or BPVF treatment in *S. aureus* (ATCC 25923) and *A. baumannii* (W704). Data were presented as the mean of three independent experiments with four replicates. **c** The efflux assay graph of PPEF or BPVF treatment in *E. coli* K12 (MG1655), *ΔyciM, lpxC*, and *lpxD*. Data were presented as the average of three independent experiments with four replicates. **d** The efflux assay graph of PPEF or BPVF treatment in *S. aureus* (ATCC 25923) and *A. baumannii* (W704). Data were presented as the mean of three independent experiments with four replicates. **e**, **f** Biofilm inhibition assay of *S. aureus* (ATCC 43300) in the presence of different concentrations of PPEF, BPVF, and CIP. Data were presented as mean ± SD of two independent experiments with four replicates.

PPEF and BPVF are fluorescent molecules. The accumulation of PPEF was measured by recording fluorescence of PPEF-treated *E. coli* K12 (MG1655), *E. coli* K12 mutants (*ΔyciM, LpxC1272, LpxD14*), *S. aureus* (ATCC 25923), and *A. baumannii* (W704) strain upto 60 min after incubation. Optimal discrimination between the accumulation of the bacterial strains was achieved at 2.5 µM concentration of PPEF and BPVF^25^. The accumulation of both compounds in *E. coli* K12 mutants was remarkably higher, compared with the parental strain (MG1655) (Fig. 5a). The nonfunctional lipid A pathway in the above three strains due to deletions/mutations are not able to synthesize lipid A and result in increased uptake of hydrophobic compounds^26^ like PPEF. Similarly, PPEF and BPVF accumulation were higher in *S. aureus* than in *A. baumannii* (Fig. 5b). The accumulation of PPEF was better than BPVF in all the strains used in this study.

The efflux of PPEF and BPVF increased considerably in the LPS-deleted/mutated *E. coli* strains *ΔyciM, LpxC1272*, and *LpxD14*, and after 15 min treatment, no further significant change in efflux was observed (Fig. 5c), but the PPEF efflux was constant in parent strains. Over-expression of efflux pumps is a common mechanism of MDR *A. baumannii*; however, we found low levels of efflux for PPEF and BPVF in both *A. baumannii* and *S. aureus* (Fig. 5d). Therefore, it can be inferred that both compounds were not rapidly effluxed from the bacterial cells.

### PPEF and BPVF exhibit antibiofilm formation activities

The antibiofilm activity of PPEF, BPVF, and CIP on *S. aureus* (ATCC 43300) was measured by determining the MBIC, defined as the minimum concentration showing a visible lack of color development. We found that when *S. aureus* (ATCC 43300) was supplemented with 0.0075–4.0 µg/mL (0.00625–6.4 µM) of either PPEF or BPVF, a decrease in biofilm formation was observed; however, as their concentration approached 1 × MIC, 100% of biofilm formation was inhibited, while CIP had MBIC values considerably higher than PPEF and BPVF (Fig. 5e, f).

### Pharmacokinetics of PPEF and BPVF

**Fig. 6:**
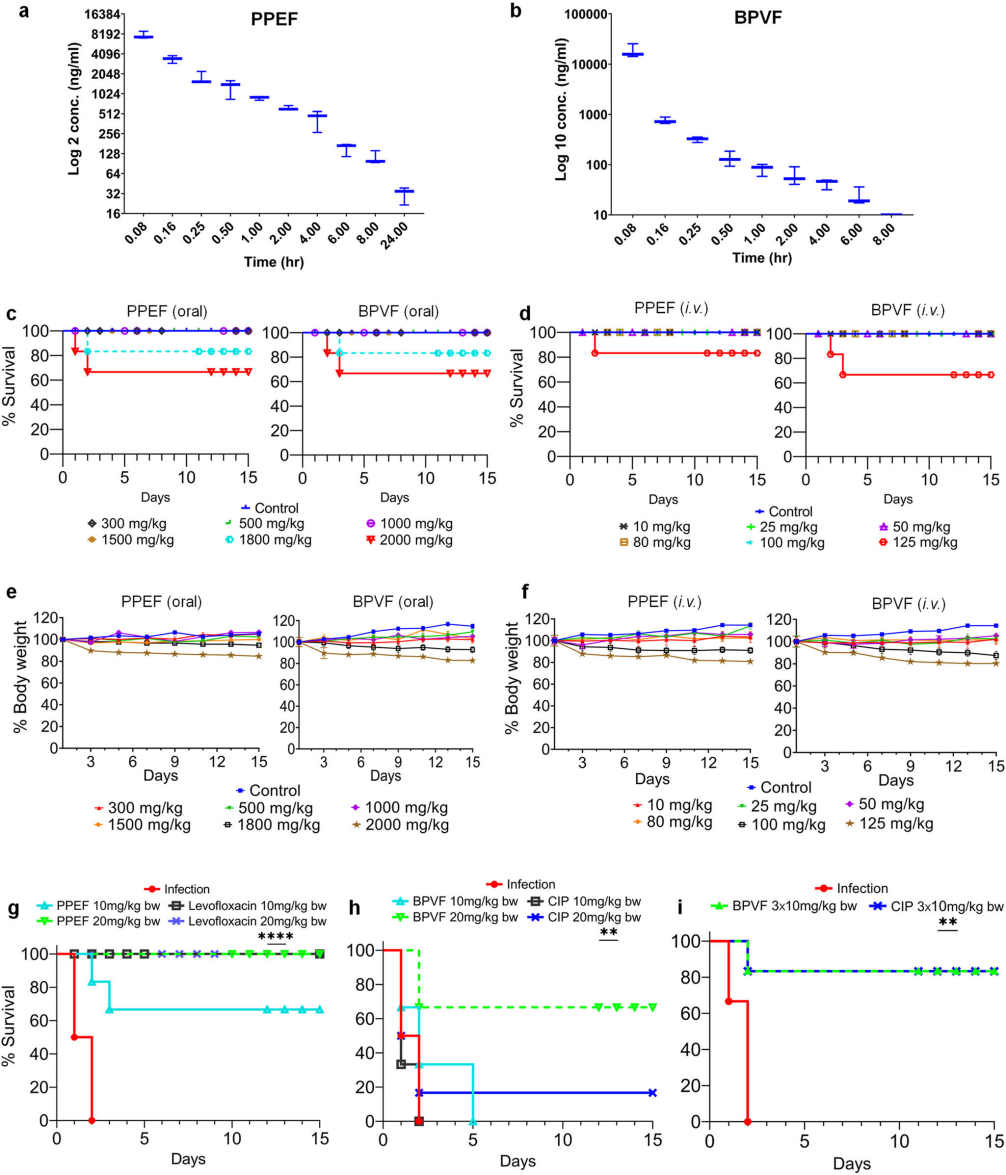

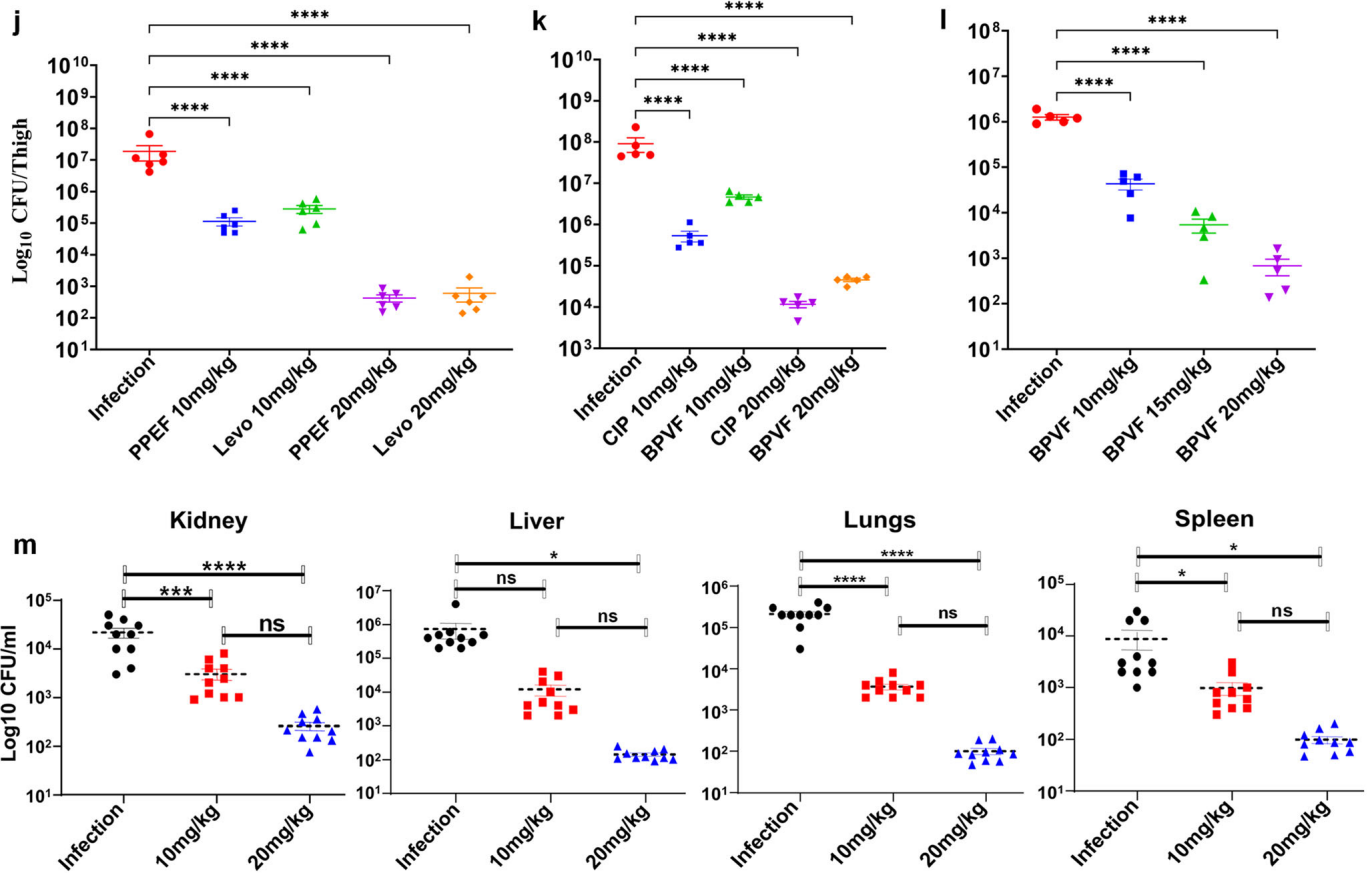
PPEF and BPVF are noncytotoxic and show efficacy against Gram-positive bacteria (MRSA) in murine sepsis and neutropenic thigh infection model. **a**, **b** Pharmacokinetic parameters and mean plasma concentration-time profiles of PPEF and BPVF after intravenous dose in Balb/c mice. Each value represents the average of six mice dosed intravenously (40 mg/kg bw and 10 mg/kg bw). **c**, **d** Percentage survival of Balb/c mice after single oral and intravenous injection of PPEF and BPVF at different doses; 300, 500,1000,1500,1800, 2000 mg/kg bw and 10, 25, 50, 80, 100, 125 mg/kg bw, respectively. **e**, **f** Percentage of body weight loss after bolus oral and intravenous injection of PPEF and BPVF at different doses; 300, 500,1000,1500,1800, 2000 mg/kg bw and 10, 25, 50, 80, 100,125 mg/kg bw, respectively. **g** The Kaplan–Meier survival graph of mice having septicemia. A total of six Swiss albino mice were inoculated by intraperitoneal injection with ~10^9^ MRSA (ATCC 43300) strain cells and then intravenous administration of PPEF and levofloxacin at 10 mg/kg bw and 20 mg/kg bw were given 2 h post-infection. Survival was monitored for 15 days after infection (****p < 0.0001 were calculated using the Log-rank test) and the ED_50_ value was calculated by Probit analysis. **h** The Kaplan–Meier survival probability plots of septicemia mice infected with MRSA (ATCC 43300): Infection (red), BPVF treated (sky blue, green), and ciprofloxacin treated (gray, blue) groups. Six Swiss albino mice were inoculated per group with ~10^9^ MRSA (ATCC 43300) strain cells and treated with BPVF and ciprofloxacin at 10 mg/kg bw and 20 mg/kg bw were given 2 h post-infection. Survival was monitored for 15 days (**p < 0.01 were calculated using the Log-rank test). **i** The Kaplan–Meier survival probability plots of sepsis infection in mice; Six mice were infected per group with ~10^9^ CFUs of MRSA (ATCC 43300): infection (red), three repeated doses (10 mg/kg) of either BPVF (green), or CIP (blue) (**p < 0.01 were calculated using Log-rank test). Data were representative of three independent experiments; *n* = 6 mice per condition. **j** Efficacy of PPEF vs levofloxacin in the neutropenic thigh mice model showing a bacterial load of MRSA (ATCC 43300) in Log CFU/g thigh tissue (*P* values were calculated using Welch two-sample *t*-test, *n* = 6). **k** Efficacy of BPVF vs CIP in the mouse neutropenic thigh model depicting VRSA (S1016) infection in terms of Log CFU/g thigh tissue (*P* values were calculated using Welch two-sample *t*-test, *n* = 5). **l** Efficacy of BPVF in the mouse neutropenic thigh model showing Log CFU/g thigh of MRSA (ATCC 43300) (*P* values were calculated using Welch two-sample *t*-test, *n* = 5). Data were representative of two independent experiments. **m** CFUs of MRSA were reduced by PPEF treatment (10 and 20 mg/kg) in the liver, spleen, lung, and kidney of infected mice. *n* = 10, *P < 0.05, **P < 0.01, and ***P < 0.001 versus control. ns not significant.

The pharmacokinetic studies were conducted to evaluate the profile of PPEF salt in the plasma of male BALB/c mice through intravenous at 40 mg/kg bw dose. The PPEF formulations were prepared in 100% (v/v) sterile water for injection. Plasma concentrations of PPEF were measured, and pharmacokinetic parameters were determined by non-compartmental analysis using Phoenix WinNonlin 6.3 software. After intravenous administration, PPEF was detected in plasma for up to 24 h. The single i.v. dose of PPEF showed plasma maximum concentrations (C_max_) of 7813 ng/mL, plasma exposure (AUC_last_) 6435.28 h*ng/mL, half-life 5.09 h, mean residence time (MRT_last_) 3.47 h and clearance 99.99 mL/min/kg (Fig. 6a and Table 2).

**Table 2:** Single-dose intravenous pharmacokinetics study of PPEF and BPVF HCl salt in male BALB/c mice.

Mean plasma PK parameters		
Parameters	PPEF HCl salt	BPVF HCl salt
Route of administration	Intravenous	Intravenous
Dose (mg/kg b.w.)	40	10
C_0_ (ng/mL)	17812.85	83218.93
C_max_ (ng/mL) at 0.08 h	7813.86	18468.67
T_max_ (h)	0.08	0.08
AUC_last_ (h*ng/mL)	6435.28	6371.49
AUC_inf_ (h*ng/mL)	6667.06	6474.95
AUC_extrap_ (%)	3.48	1.60
T_1/2_ (h)	5.09	2.98
MRT_last_ (h)	3.47	0.29
Cl_obs (mL/min/kg)	99.99	25.74
Vss_obs (L/kg)	26.61	0.74

Subsequently, pharmacokinetic studies were done with BPVF salt in the plasma of male BALB/c mice through i.v. at 10 mg/kg bw dose, preparing the formulations in 100% (v/v) sterile water. The analysis of results was done as mentioned above for PPEF. In a single-dose intravenous pharmacokinetics study of BPVF salt at 10 mg/kg bw, the plasma maximum concentrations (C_max_) 18,468 ng/mL, plasma exposure (AUC_last_) 6371.5 h*ng/mL, half-life 2.97 h, mean residence time (MRT_last_) 0.29 h and clearance 25.74 mL/min/kg were observed (Fig. 6b and Table 2).

### PPEF and BPVF are safe for BALB/c mice

PPEF and BPVF did not cause mortality or toxicity symptoms such as abnormal demeanor and behaviors at oral doses; 300, 500,1000,1500,1800, and 2000 mg/kg bw in Balb/c mice (limit dose according to the OECD test number 423 guidelines). Therefore, the oral LD_50_ for both compounds were >2000 mg/kg bw (Supplementary Table 5 and Fig. 6c). Food and water intake were normal, and the body weights of the animals did not change for up to 15 days (Fig. 6e).

The intravenous toxicity in mice was recorded at six different concentrations of PPEF; 10, 25, 50, 80, 100, 125, and 150 mg/kg BW, and BPVF; 10, 25, 50, 80, 100, 125, and 150 mg/kg bw (Fig. 6d). All mice survived for 15 days without any abnormalities upto 100 mg/kg bw dose (Fig. 6f and Table 3). But 01 mice died out of six animals in the PPEF group but 02 died in the BPVF group at 125 mg/kg bw dose, rest of the mice survived, and none of them showed any abnormalities and were healthy till they were alive. At 150 mg/kg bw 04 animals died in each group. Therefore, the LD_50_ for i.v. injection for both compounds was >125 mg/kg bw. The body weight of PPEF and BPVF-treated mice increased progressively throughout the study. After the initial dosage, the behavioral observation of the test animals showed an elevated respiration rate for the first 15 min in the BPVF-treated group. Neither group displayed drowsiness, sleepiness, itching, or shivering behavior. The therapeutic index (TI) of PPEF and BPVF was observed at 12.5 and 6.25, respectively, for IV injection (Supplementary Table 5). The higher therapeutics index suggests that the margin of safety of the drug is wider and can be also used at repeated dose.

**Table 3:** Behavioral patterns of PPEF and BPVF injected in a group of mice.

Parameters	Observation of vehicle control and PPEF-treated groups					
	PPEF	BPVF	PPEF	BPVF	PPEF	BPVF
	Day 1	Day 1	Day 1	Day 1	Day 1	Day 1
	CG TG	CG TG	CG TG	CG TG	CG TG	CG TG
Fur & skin	N N	N N	N N	N N	N N	N N
Eyes	N N	N N	N N	N N	N N	N N
Salivation	N N	N N	N N	N N	N N	N N
Respiration	N N	N N	N N	N N	N N	N N
Urination (color)	N N	N N	N N	N N	N N	N N
Feces consistency	N N	N N	N N	N N	N N	N N
Sleep	N N	N N	N N	N N	N N	N N
Mucous membrane	N N	N N	N N	N N	N N	N N
Convulsions & tremors	NF NF	NF NF	NF NF	NF NF	NF NF	NF NF
Itching	NF NF	NF NF	NF NF	NF NF	NF NF	NF NF
Coma	NF NF	NF NF	NF NF	NF NF	NF NF	NF NF
Mortality	NF NF	NF NF	NF NF	NF NF	NF NF	NF NF

CG control group, TG treated group, N normal, NF not found.

### PPEF and BPVF exhibit less cytotoxicity and hemolytic activity

Cell toxicity assay in NIH/3T3, HepG2, A549, and HEK293Tcell line, at three different concentrations (25, 50,100 µM) showed decreased cell survival with increased BPVF concentration (Supplementary Fig. 12a). PPEF is less cytotoxic to mammalian cells at a therapeutic dose (Supplementary Fig. 12a).

In vitro hemolysis experiments were performed by treating human erythrocytes with PPEF, BPVF, and ciprofloxacin (Supplementary Fig. 12b). Less than 1% hemolysis of erythrocyte cells was observed in PPEF-and BPVF-treated cells at 128 μg/mL (mean hemolysis: PPEF = 1.18%, BPVF = 1.01%). Hemolysis of erythrocyte cells was more toxic to CIP (mean hemolysis at 128 μg/mL: CIP = 2.44%) than PPEF and BPVF.

### PPEF and BPVF protect against MRSA infection in sepsis mice models

The efficacy of PPEF and BPVF was evaluated in sepsis mouse infection models that mimic systemic and localized bacterial infections (MRSA) in humans. In order to determine the ED_50_ of PPEF and BPVF, septicemic mice were treated at a single dose of 10 and 20 mg/kg bw dose of PPEF, BPVF, Ciprofloxacin, and levofloxacin. 100% of the mice survived at 10 and 20 mg/kg bw dose of Levofloxacin, whereas 66.6% of mice survived at 10 mg/kg dose and 100% of mice survived at 20 mg/kg bw dose of PPEF (Fig. 6g). The in vivo efficacy of PPEF and BPVF on *E. coli* ATCC^27^ reported in Supplementary Fig. 13. BPVF and CIP were not able to protect mice at 10 mg/kg BW dose. But BPVF at a single dose of 20 mg/kg BW protected the 66.6% *S. aureus* (ATCC 43300) infected animals (Fig. 6h). In contrast, CIP at equivalent doses was not effective in saving the animals. Interestingly 80% of the mice survived at the three repeated doses (3 × 10 mg/kg bw dose of BPVF and CIP given at 2, 24, and 48 h post-infection to mice, *n* = 6 animal) (Fig. 6i). This suggests that these molecules can be more efficacious at repeated dose.

### PPEF and BPVF protect against MRSA and *E. coli* infection in neutropenic thigh mice models

In the neutropenic thigh MRSA (ATCC 43300) infection mice model, a 2-log-fold decrease in infection occurred at 10 mg/kg bw and 4 log fold at 20 mg/kg bw dose of PPEF and levofloxacin (Fig. 6j). In the neutropenic thigh VRSA (S1016) infection mice model, ~2 log fold decrease in infection occurred at 10 mg/kg bw and 4 log fold at 20 mg/kg bw dose of BPVF and CIP (Fig. 6k).The 1, 2.2, and 4 Log CFU/g reduction of MRSA (ATCC 43300) infection was observed in the neutropenic thigh model at a dose of 10, 15, and 20 mg/kg bw of BPVF (Fig. 6l). Administration of PPEF at doses of 10 and 20 mg/kg bw could inhibit MRSA and inhibit the spread and growth of MRSA infection in liver, spleen, lung, and kidney (Fig. 6m).

## Discussion

The proliferation of drug-resistant pathogens has created an urgent need for antibiotics targeting novel receptors in bacteria. We have synthesized a series of bisbenzimidazole derivatives in our laboratory with different piperazines at one end and aromatic aldehydes with varying substituents on the phenyl ring at the other. The aldehydes used for the second half carried different lengths of carbon side chains and substituents on the phenyl ring to obtain a molecule with significant antibacterial efficacy given in Supplementary Scheme 1–3. PPEF and BPVF molecules were characterized and given in Supplementary Notes 1, 2 and Fig. 1a–f (^1^H NMR,^13^C NMR, and HRMS). The increased carbon chain length creates more hydrophobic molecules, as exemplified by PPEF and BPVF. Apart from DNA binding, the protonation of piperazine moiety leads to positively charged molecules at physiological pH, thereby increasing the affinity to bind DNA. PPEF and BPVF showed significant antibacterial efficacy from that library against seven WHO-identified priority pathogens. The PPEF and BPVF showed potent activity against methicillin and vancomycin sensitivity and resistance against *S. aureus*. As TopoIA are novel drug targets, inhibition of DNA TopoIA by PPEF and BPVF warrants singular attention. The ability of PPEF and BPVF to specifically inhibit type IA topoisomerase activity has been demonstrated in in vitro analysis. Inhibition of the relaxation activity of topoisomerase can lead to bacterial cell death^28–30^. PPEF and BPVF also constrain EcTopoIII activity, which bolsters cell survival capacity in the presence of defective TopoIA^31,32^.

Our results suggest that PPEF and BPVF inhibit the relaxation activity of type IA topoisomerase by shifting the religation and increasing the accumulation of DNA products. These molecules act through an interfacial inhibitor to facilitate the trapping of the DNA–enzyme complex. Also, PPEF showed better in vitro antibacterial efficacy than BPVF. In the native (closed) structure, the free energy of interaction energy between domain1 and domain2 is about –38 kcal mol^–1^ (Fig. 1c). The binding of PPEF to TopoIA leads to compaction with the increase in the value of **NC** and lowering of the RMSD (from 20 to 8 Å, Supplementary Fig. 4) resulting in a net lowering of the free energy by –6 kcal mol^–1^. On the other hand, the binding of ssDNA (and dsDNA) greatly lowers the interaction between domain1 and domain2, which is the signature of the partial gate opening, which is marginally compensated by the interaction of ssDNA (and dsDNA) with the protein. However, the binding free energy of the ssDNA (and dsDNA) to TopoIA does not favor the formation of a completely open conformation of the protein. Enumerating, the interaction between the protein and PPEF was scored at the protein residues D111, D113, E115, K303, D323, L482, and I504, whereas residues R168, R310, and I394 interact with ssDNA (Fig. 1g, h, i and Supplementary Fig. 11). The position of Mg^2+^, which is about 9 ± 4 Å from the D111 residue of the protein, is not affected by the binding of PPEF and ssDNA, which agrees with the crystal structure data. The disposition of Y319 in the ternary complex with PPEF and ssDNA relative to the DDE motif and 3’-hydroxyl of DNA does not favor the proton transfer required for DNA religation, thereby leading to an increase in damaged DNA. The PPEF in the, TopoIA-dsDNA-PPEF ternary complex binds to the cleft formed by E479, A480, S481, V483, K484, A498, I501, S502, Q505, Y509, R515, and F517 (Supplementary Fig. 14c, d). On the other hand, in the TopoIA-ssDNA-PPEF ternary complex PPEF binds to the cleft formed by V301, K302, M305, M306, Q309, R321, A498, I501, S502, I504, Q505, Y509, R515, and F517 (Supplementary Fig. 14e, f). The hydrophobic interactions of V301, M305, M306, I501, I504, and F517 with PPEF remained stable in the closed and partially open states of TopoIA-PPEF and TopoIA-ssDNA-PPEF.

It has been shown in the literature that compounds that enhance ssDNA cleavage or TopoIA-ssDNA covalent linkages can be effective lead molecules for developing new antibacterial agents. We suggest that these compounds act by altering the perceived topological state of the pHOT-1, making underwound DNA appear to be overwound. So, it is proposed that these compounds be referred to as topological inhibitors of type IA topoisomerase. Fluorescence microscopy and flow cytometry data confirmed that BPVF arrests cell division and DNA fragmentation, where the generation of focal points suggests chromosomal condensation, a distinct marker of its bactericidal effect^33^. Time-dependent killing shows the efficacy of PPEF and BPVF in *S. aureus* and *E. coli*, marked by accelerated uptake and sustained retention in the cell—a possible mechanism for the competent activity of both molecules against MDR strains.

Previous studies demonstrated that in the case of efflux-acquired resistance, compounds that increase outer membrane permeability would efficiently facilitate antibiotic penetration into bacterial cells and increase susceptibility^34–36^. Drug efflux and susceptibility are dependent on porins and disruption of the outer membrane lipopolysaccharide (LPS) barrier^37^. Therefore, the chemical composition of a drug is essential for drug uptake. Cationic antibacterial agents interact with bacterial cell membranes via LPS bridges. This causes perturbation and permeabilization of the outer membrane and increases the uptake of charged antimicrobial agents into the bacterial cell^33^. Bacterial strains such as *E. coli* and *S. typhimurium* cannot synthesize lipid A and is without the inner core of LPS, making them sensitive to many antibiotics^37,38^. Similarly, the *E. coli* mutants (ΔyciM, LpxC1272, and LpxD14) showed a high accumulation of PPEF and low MIC against PPEF and BPVF. It was already reported that porin-deprived strains also had a significantly lower MIC than the isogenic wild-type strain. This shows that bisbenzimidazole does not necessarily permeate through porins. The hydrophobicity and cationic charge on the nitrogen of the piperazine ring of the bisbenzimidazole derivatives interact strongly with LPS and cross the negatively charged bacterial cell membrane in Gram-negative bacteria.

*S. aureus* biofilms are often associated with chronic infections and contaminating implanted medical devices. The presence of biofilms renders the bacteria highly tolerant to antibiotics and can resist phagocytosis. PPEF and BPVF also showed considerable activity against Gram-positive *S. aureus* (MRSA and VRSA) and inhibited biofilm formation. Based on these results, it is reasonable to suggest that PPEF and BPVF might help in controlling biofilms in chronic wounds infected with multiple bacterial species. The simultaneous efficacy and low toxicity of PPEF and BPVF are unique properties. We found that the LD_50_ of both compounds was 100 times higher than that of the MIC and MBC, whereas the TI of PPEF was especially significant. Moreover, both compounds were non-toxic to RBCs and displayed remarkable safety profiles. This suggests that these molecules are suitable for internal applications. Furthermore, no mortality was observed for doses upto 2000 mg/kg BW, indicating that these molecules are safe for humans as per OECD guidelines (#423). In addition, the levofloxacin-treated mice showed 100% overall survival, whereas PPEF-treated mice showed 100% survival. The low toxicity is a unique property that is not displayed by any other clinical antibiotic. The pharmacokinetics data suggest good absorption at intravenous doses with higher plasmatic C_max_ value recorded for PPEF (7813 ng/ml) and BPVF (18468.67) compared to CPT-11 (6571 ng/ml)^39^. The increased value of t_1/2_ for PPEF and BPVF indicates a proportional decrease in clearance, posing its more availability for its effective action and a proportional decrease in the maintenance dose. The above findings support the direction of clinical use of PPEF and BPVF.

## Methods

### Molecular dynamics simulations

The molecular dynamics (MD) simulations were carried out using double-precision Gromacs 2020.2^1^patched with plumed-2.6^17,40^ for the simulations and free energy calculations. The TopoIA (PDB: 3PX7) has some missing residues close to the binding site, which was fixed using the Modeler^41^, which gave the five best structures, among which the lowest energy conformation was considered for further simulations. The DNA sequence(5′ -AATGCGCT ↓ TTGGG-3′) was modeled as a single-strand DNA (ssDNA) and was also modeled as double-strand DNA (dsDNA) by adding the complementary sequence. The bisbenzimidazole ligand (PPEF) was docked to the dsDNA and protein separately using HDDOCK server^42^. Additionally, the dsDNA–protein and ssDNA–protein complexes were also formed using HDDOCK server. For all the simulations reported in this work, the appropriate system was placed in a cubic box, and the length of the box was adjusted such that even in the dissociated state, the system does not interact with its image. The box was solvated with TIP3P water model^43^, and 10 mM of MgCl_2_. The system was energy minimized using the steepest descent method^44^, followed by heating to 300 K for 100 ps in the NVT ensemble using Berendsen thermostat^45^ with 0.6 ps coupling constant. During the heating process, a harmonic restrain of 105 kJ mol^−1^ Å^−2^ was applied to the heavy atoms of all the components present in the system (such as DNA, protein, and ligand). The applied restraint was removed in six steps. Each step follows an equilibration of 200 ps with an energy minimization of 1000 steps. Further, a 10 ns NPT equilibration was carried out using the Nose–Hoover the most at ref. ^46^ and Parrinello–Rahman barostat^47^ with a coupling constant of 0.6 ps. Thereafter, a 100 ns production run was carried out using Nose–Hoover thermostat with a coupling constant of 0.6 ps. In all simulations, the electrostatics were treated by particle mesh Ewald (PME) method^48^ with the long-range cut-off of 10 Å. Whereas a 10 Å cut-off was used to van der Waals interaction. During simulation, all the covalent bonds were constrained using the LINC algorithm^49^. For the present set of simulations, amber99 force field with bsc0^50,51^ correction, amber14sb^52^, and generalized amber force field (gaff)^53^ were used for the DNA, protein, and ligand, respectively. The amber and modified amber force fields and the TIP3P water model are generally used as common force fields in MD simulations of proteins and DNA. Three independent sets of simulations with different initial conditions for carried out for each system, with a total of 18 simulations of 100 ns each. Supplementary Fig. 1 shows the root mean square deviation (RMSD) plots for all the six systems considered in the present work. The RMSD plots over the 100 ns trajectory also indicate that in all the cases, the simulations have converged. In an effort to capture various dynamics for the protein, protein–DNA, protein–ligand, DNA–ligand binary complexes, and protein–DNA–ligand ternary complex, two reaction coordinates were employed, which are illustrated in Fig.1. The reaction coordinate ***X*** for the protein is the projection of the vector $\overrightarrow{a}$ that joins the center of masses of the residues 1–100 (purple color in Fig.1b) to the center of masses of the residues 350–450 (cyan color in Fig. 1b) on to a body-fixed unit vector$\hat{b}$, which joins the center of masses of the residues 1–100 (purple color in Fig. 1b) to the center of masses of the residues 1–50 (the green color region in Fig. 1b). The second reaction coordinate is native contacts (**NC***)*. The **NC** is defined by the spatial proximity of groups of atoms in the native state. The native contact between the heavy atoms of domain1 and the heavy atoms of domain2 is defined by using equations,

1 $${NC}=\mathop{\sum}\limits_{i\in G1}\mathop{\sum}\limits_{j\in G2}{s}_{ij},$$

where, *s*_*ij*_ is given by,

2 $${s}_{ij}=\left\{\begin{array}{cc}1{r}_{ij}\le 0\\ \frac{1-{(\frac{{r}_{ij}}{{r}_{0}})}^{n}}{1-{(\frac{{r}_{ij}}{{r}_{0}})}^{m}}{r}_{ij} \, > \, 0\end{array}\right.$$

and *r*_*ij*_ = |*r*_*i*_ − *r*_*j*_| − *d*_0_. In the current definition, we have chosen to be *n* = 6, *m* = 12, *r*_0_ = 0.5 Å, and *d*_0_ = 5 Å. Above equation ensures the variation of *s*_*ij*_ is continuous and differentiable. To calculate **NC**, used a distance cutoff of 5.5 Å between the heavy atoms of the residues 1–224 and 463–597 (the red color region in Fig. 1a and from now onward defined as domain1) to the residues 284–405 (the purple color region in Fig. 1a and from now onward defined as domain2) was used. Since the native contacts are a unique feature of only the native state, the correct number of **NC** ensures that the system is in the native crystal geometry. To begin with, multiple unrestrained MD simulations were carried out on all the systems for 100 ns and the supplementary Fig. 2 shows that both X and NC do not show any changes, which indicates the protein does undergo any significant change in the presence of dsDNA/ssDNA/PPEF. Therefore, free energy calculations were carried out along the desirable reaction coordinates ***X***, with biasing Gaussian potential of width 2 Å and 50 for the reaction coordinate *NC*. The height of the Gaussian potential was varied from 1.2 to 0.0 kJ mol^−1^ to sample the configuration space around the reaction coordinate, which severs a convergence criterion. Finally, the sum hills module of Plumed was used to construct the free energy surface, and the minimum free energy path was constructed using an algorithm given by ref. ^54^ Additionally, the interaction energy between the different residues of TopoIA and its complexes with dsDNA, ssDNA, and ligand was evaluated using the sum of pairwise electrostatic and van der Waals energy.

Various molecular level interactions in the ternary complexes can be enumerated as The PPEF in the TopoIA-dsDNA-PPEF ternary complex binds to the cleft formed by E479, A480, S481, V483, K484, A498, I501, S502, Q505, Y509, R515, and F517 (supplementary fig. 14c–d). On the other hand, in the TopoIA-ssDNA-PPEF ternary complex PPEF binds to the cleft formed by V301, K302, M305, M306, Q309, R321, A498, I501,S502, I504, Q505, Y509, R515, and F517 (supplementary fig. 14e–f). The hydrophobic interactions of V301, M305, M306, I501, I504, and F517 with PPEF remained stable in the closed and partially open states of TopoIA-PPEF and TopoIA-ssDNA-PPEF.

### Bacterial strains

*E. coli* (ATCC 25922), *S. aureus* ATCC 25923 and ATCC 43300 (MRSA), *S. typhimurium* (MTCC 1251), *S. flexneri* (MTCC 1457), and Enterococcus (MCC 2105) were procured from the bacterial repository. The MDR strains of *Entetococcus* sp, *Staphylococcus* sp., *E. coli*, *S. typhimurium*, *S. flexneri*, *Acinetobacter baumannii, Klebsiella spp*., were obtained from the Hospital. *E. coli* ESBL-producing bacteria NCTC11954/ATCC 35218 (Augmentin β-lactamase positive Ticarcillin, β-lactamase positive), NCTC13351 (TEM-3, ESBL), NCTC13400 (CTX-M-15, blaCTX-M-15, blaOXA-1, catB4, tet(A), blaTEM-1, aac6′-Ib-cr, mph(A), and integron-borne dfrA7, aadA5 and sulI genes), NCTC13352 (TEM-10 ESBL), NCTC13451(blaCTX-M-15, blaTEM-1, blaOXA-1, catB4, tet(A), aac6′-Ib-cr, mph(A), and integron-borne dfrA7, aadA5 and sulI genes), NCTC13461 (CTX-M), NCTC13462 (CTX-M), NCTC13476 (MP-type), NCTC13846 (MCR-1 positive), NCTC13919 (blaGES-5, non-metallo-β-lactamase) are purchased from National Collection of Type Cultures, public health England. Porin deleted strains *ΔompC*(CGSC 9781, ΔompC768::kan), *ΔompF* (CGSC 8925, ΔompF746::kan), efflux gene deleted strains, *ΔemrA* (CGSC 10098, ΔemrA766::kan), *ΔacrA* (CGSC 11843, ΔacrA748::kan), ΔtolC (CGSC 11430, ΔacrA748::kan), delete the promoter and the N-terminal part of TopI, *ΔtopA*(CGSC 8229,topA75 zci-2234::cat), topoIII deleted strain, *ΔtopB* (CGSC 9474, ΔtopB761::kan), wild-type *E. coli* K12 (CGSC 5073) were obtained from *E. coli* Genetic Stock Center, Yale University, USA. *M. tuberculosis* H37Ra and H37Rv were a kind gift. The *yciM* was deleted to generate MG1655 *ΔyciM* (BW25113ΔyciM::Kan) strain, *yciM* is a gene encoding a tetratricopeptide repeat protein which modulates LPS levels by negatively regulating the biosynthesis of lipid A, MG1655 LpxC1272(JW1272*yciM* + KanStrpB83::Tn10) strain was developed by mutating at *lpxC*-I186N (Isoleucine to Asparagine) in a gene of UDP-3-O-acyl-*N*-acetylglucosamine deacetylase (LpxC) catalyzes the lipid A biosynthesis and similarly, MG1655LpxD14 (skp::Tn10dTet) strain was developed by an insertion after codon 79 in a gene of UDP-3-O-(3-hydroxymyristoyl)glucosamine *N*-acetyltransferase (LpxD) catalyzes lipid A biosynthesis by converting of UDP-3-O-(3-hydroxymyristoyl)glucosamine to UDP-2,3-bis(3-hydroxymyristoyl) glucosamine. These strains were a kind gift. pHOT-1 plasmid DNA was purchased from TopoGen Inc., Port Orange, FL, USA. E. coli DNA gyrase and its relaxed substrate (DNA) were purchased from New England Biolabs, Germany. The sources of different chemicals, reagents, cells, plasmids, software, antibiotics, etc., are given table (Materials Details). All animal experiments were approved by the Animal ethical committee using ethical guidelines. Animals were maintained under controlled conditions with free access to food and water.

### Culture conditions

The goal of the study was to develop an antibacterial agent against WHO-priority pathogens. We prepared the clones of *S. aureus* topoisomerase IA (SaTopoIA), *A. baumannii* topoisomerase IA (AbTopoIA), *E. coli* topoisomerase IA (EcTopoIA), and *E. coli* topoisomerase III (EcTopoIII). pET28a(+) vector was used for cloning and confirmed the clones by Sanger’s sequencing. Clones were expressed in the BL21 strain and purified by Ni-NTA agarose beads. For all experiments, cultures were grown in either LB broth or MH broth overnight at 37 °C with shaking at 200 rpm, then subcultured and adjusted for further investigations at the required OD_600_. 1×MIC_90_ for PPEF against *S. aureus* was 0.5 µg/mL (0.8 µM), and for *E. coli*8µg/mL (12.9 µM), similarly, 1×MIC_90_ for BPVF against *S. aureus* was 1 µg/mL (1.6 µM) and for *E. coli* 16 µg/mL (25.8 µM).

### Cell lines and culture conditions

The metastatic A549 human epithelial lung carcinoma cell line was obtained from ATCC, USA. Whereas NIH3T3 mouse fibroblast, HEK293 human epithelial kidney, and HEPG2 human epithelial hepatocellular carcinoma cell lines were purchased from NCCS Pune, India. No Mycoplasma contamination was detected using the Look Out Mycoplasma PCR detection kit (Sigma, Catalog Number MP0035) every 6 months. All cell lines used for experiments were not cultured for more than 30 passages. Cells were maintained in DMEM supplemented with 10% FBS and 1X antibiotic, antimycotic solution at 37 °C in a humidified atmosphere containing 5% CO_2._

### Antibacterial susceptibility test

Minimal inhibitory concentration (MIC) was determined by broth microdilution method according to CLSI methods^19^ and through E-test^20^. The bacterial suspensions of 1.0 × 10^6^ CFU per/well were seeded in 96-well plates (Corning® 96-well Clear Polystyrene Microplates) at an increasing concentration range of 0.25–128 µg/mL (0.4–206.4 μM) of PPEF and BPVF, incubated at 37 °C for 24 h. MIC values were scored as the minimal concentration at which no visible growth of bacterium was observed and detected by Tecan Microplate Reader at 600 nm. The absolute MIC value was scored as the minimal concentration that inhibited growth to 90% at 24 h. The E-test was used for the detection of MIC of the standard drug^20^.

### Determination of minimal bactericidal concentrations (MBC)

MBC was calculated using the literature method. Reference MBC for each strain was determined by pelleting the culture of MIC_50_ value and three values beyond MIC_50_ values. The pellet was resuspended in PPEF and BPVF-free MH broth and washed thrice to remove PPEF and BPVF. Then the PPEF and BPVF-free pellets were again resuspended in fresh 100 μL of MH broth and plated on MH agar plates without PPEF and BPVF. MBC endpoints were read as the lowest dilution of the compound with no growth (>99.9% killing) after overnight incubation at 37 °C using culture without PPEF and BPVF as control^26^.

### Gel-based assay of oligonucleotide DNA product accumulation in the presence of inhibitors

The effect of PPEF and BPVF on the DNA-religation equilibrium of SaTopoIA was assayed using a 5-^32^P-labeled 58 bp single-stranded DNA substrate. The labelled DNA (50 ng) was incubated with 100 ng of SaTopoIA in 5 µL of 10 mM Tris, pH 8.0, at 37 °C for 30 min to allow the formation of the DNA product. PPEF and BPVF were added to each reaction and incubated for 2 min, followed by the addition of 2 mM MgCl_2_. After further incubation at 37 °C for 5 min, the reaction was terminated by the addition of 6.5 µL of sequencing gel loading buffer. The level of DNA products was analyzed by electrophoresis in a 15% sequencing gel followed by Phosphor-Imager analysis of the dried gel^55^.

### Cleavage assay of pHOT-1 plasmid by SaTopoIA and EcTopoIII

The 350 ng of supercoiled pHOT-1 plasmid was added to 800 ng of SaTopoIAand1000 ng of EcTopoIII in an assay buffer (10 mM Tris-HCl, pH 7.9, 5% glycerol, 150 mM NaCl, 1 mM EDTA, 0.1% BSA, 0.1 mM spermidine) respectively. The PPEF and BPVF were added in increasing concentrations (1, 5, 10, 25, 50, 75, and 100 μM) to the above reaction mixture, and the reaction volume was adjusted to 20 ul for 30 min at 37 °C. The reactions were terminated by adding 50 μg/mL proteinase K and 0.5% SDS and incubated for 1 h at 30 °C. Gel loading dye was added to each reaction mix and further analysed using 1% agarose gel containing 0.5 μg/mL of EtBr and electrophoresed at 1.5 V/cm for 15 h. The gel was photographed over UV light using Alpha Imager (Cell Biosciences) for analysis^56^.

### DNA quantification using flow cytometry

The cultures (*E. coli* (MG1655) and *S. aureus* (ATCC43300} were grown by the aforementioned procedure, and subsequently, the OD_600_ was adjusted to 0.1 OD_600._ BPVF and PPEF (1×MIC) were added to 1 mL suspension of bacterial cells at 37 °C. Treated cells were collected at 3 h; cultures were centrifuged for 10 min at 4 °C, 2500 rpm, and the pellets were resuspended in 1.0 mL of PBS. Then 100 μL of the cell suspension was fixed with 70% (v/v) ethanol. The fixed cells were centrifuged at 6000 rpm for 10 min at 25 °C, ethanol was decanted, and residual ethanol was evaporated. The cell pellet was resuspended in 100 μL PBS and was stained with PicoGreen, a fluorochrome that selectively binds dsDNA, made the volume up to 1 mL using PBS; the samples were analysed using a Becton Dickinson LSR Fortessa flow cytometer^32^. The 20,000 events were collected. Data were analysed using FCS Express 7 (De Novo Software, USA), and each sample was plotted as a histogram of the number of cells against the green fluorescence intensity.

### Analysis of DNA fragmentation

Cells were grown by the aforementioned procedure^22^, and subsequently, the OD_600_ was adjusted to 0.1. PPEF (1×MIC) was added to 1 mL suspension of *E. coli* (MG1655) and incubated at 37 °C. Treated cells were collected at 4.5 h; cultures were centrifuged for 10 min at 4 °C, 2500 rpm, and the pellets were resuspended in 1.0 mL of PBS. Suspend the cells in 1% (w/v) paraformaldehyde in PBS (pH 7.4) at a concentration of 1–2 × 10^6^ cells/ml. Place the cell suspension on ice for 30–60 min. Centrifuge cells for 5 min at 300×*g* and discard the supernatant. Wash the cells in 5 ml of PBS, then pellet the cells by centrifugation. Repeat the wash and centrifugation. Resuspend the cell pellet in the residual PBS in the tube by gently vertexing the tube. Adjust the cell concentration to 1–2 × 10^6^ cells/ml in 70% (v/v) ice-cold ethanol. Let cells stand for a minimum of 30 min on ice or in the freezer. Then the cell suspension was fixed with 70% (v/v) ethanol used for terminal deoxynucleotide transferase dUTP nick-end labeling (TUNEL). Labeling of DNA fragments was performed using the APO-BRDUKit, which employs FITC-conjugated deoxyuridine triphosphate (FITC-dUTP). (Becton Dickinson). Propidium iodide (PI; total cellular DNA) and FITC (apoptotic cells) are the two dyes being used. PI fluoresces at about 623 nm and FITC at 520 nm when excited at 488 nm. We collect 10,000 events. The percentage of DNA-damaged cells was plotted using GraphPad 9 (San Diego, CA, USA).

### Cloning of SaTopoIA, AbTopoIA, and EcTopoIII

In the present study, we have cloned, expressed, and purified SaTopoIA (79,071 kDa), AbTopoIA (97,835 kDa), and EcTopoIII (73,217 kDa) genes from their respective genomic DNA. We have cloned all topoisomerase genes in the pET28a(+) vector having N-terminal His-tag. SaTopoIA was amplified using the SaTopoIA gene with forward primer (FPSaTopoIA: 5′-TGCATCATATGTTGGCAGATAATTTAGTCATTG-3′) and reverse primer (RPSaTopoIA: 5′-CGGGGTACCTTATTTCTGCGCTGCCTCTTTATC-3′) covering of 2070 bp (Restriction sites CATATG- NdeI, GGTACC-BamHI) (Fig. S9A). AbTopoIA was amplified by using the AbTopoIA gene with forward primer (FPAbTopoIA: 5′-CACAGAGGATCCATGGCGAATACCTG-3′) and reverse primer (RPAbTopoIA: 5′-CACAGACTCGAGTTAGCCTTCAATCCATTTACCC-3′) covering 2637 bp (Restriction sites CTCGAG- XhoI, GGTACC-BamHI) (Fig. S9B). EcTopoIII gene was amplified by using forward primer (FPEcTopoIII: 5′-GAATCATATGCGGTTGTTT-3′) and reverse primer (RPEcTopoIII: 5′-AATTCTCGAGCGCTATCC-3′) covering 1962 bp (Restriction sites CTCGAG- XhoI, GGTACC-BamHI) (Fig. S9C). The clones were confirmed by Sanger sequencing (Fig. S9D). All the above clones were transformed in *E. coli* BL21 strain in LB containing 50 μg/mL kanamycin at 37 °C until OD_600_ reached a level of 0.6–0.8. Isopropyl β-d-1-thiogalactopyranoside (IPTG) was then added to the final concentration of 1 mM in culture for an additional 3 h at 37 °C in a shaker incubator. The cells were lysed in lysis buffer containing 1 mM DTT, 2 mM benzamidine, 2 mM PMSF, and a protease inhibitor cocktail. The 1 mg/mL lysozyme was added for the lysis of cells and kept on ice for 30 min. Dipped in liquid N_2_ for 10 min and thawed under flowing warm water followed by sonication. The cells were sonicated on ice, the sonicated lysate was centrifuged at 12,000 rpm for 30 min and the supernatant was checked for expression of proteins. The supernatant was incubated for 3 h with pre-equilibrated Ni-NTA in lysis buffer with constant rotatory motion at 4 °C. After incubation, resin bound to protein was washed with 50 mL wash buffer (50 mM Tris-Cl pH 8.0, 150 mM NaCl, 1 mM DTT, 10% glycerol, 1 mM PMSF). Proteins were eluted with elution buffer (50 mM Tris-Cl pH 8.0, 150 mM NaCl, 1 mM DTT, 20% glycerol, 1 mM PMSF, protease inhibitor cocktail, and imidazole). Fractions were analysed by 10% SDS-PAGE. The protein samples were further digested with thrombin, then concentrated and desalted with Amicon (Millipore) concentrator 50 kDa for further experiments.

### SaTopoIA, AbTopoIA, EcTopoIA, and EcTopoIII relaxation assay in the presence of PPEF and BPVF

SaTopoIA, AbTopoIA, EcTopoIA, and EcTopoIII proteins were cloned, expressed in pET28a(+), purified, and diluted in 10 mM Tris-HCl pH 8.0, 50 mM NaCl, 0.1 mg/mL gelatin, 0.5 mM MgCl_2_ buffer. PPEF and BPVF were added in increasing concentrations (0.75, 1, 5, 10, 25, 50, 75 µM) to topoisomerase enzyme (SaTopoIA-150 ng, AbTopoIA-150 ng, EcTopoIA-150 ng, and EcTopoIII-250 ng) and 350 ng of supercoiled pHOT-1 plasmid DNA, and reaction volume was adjusted to 20 µl. The mixture was incubated at 37 °C for 30 min, terminated by adding 10 mM EDTA, 0.5% SDS, 0.25 μg/mL bromophenol blue, and analysed by agarose gel electrophoresis. The ethidium bromide-stained gel was photographed over a UV transilluminator^57,58^. The relaxation assay was done without EtBr and shown in Fig. 3a–f. A similar experiment was done in with EtBr and shown in Supplementary Fig. 11.

### Fluorescence microscopy

Analysis of PPEF and BPVF-treated bacterial cell (MG1655) morphology. The cultures were grown by the aforementioned procedure, and subsequently, the OD_600_ was adjusted to 0.2. The cultures were treated with 1 × MIC of PPEF and BPVF, and samples were collected at 1.5, 3.0, and 4.5 h post-treatment. PPEF and BPVF are fluorescent; it requires no further staining for visibility. After staining, the cells were analysed with a Zeiss AxioImagerZI microscope under 100 x magnifications^22,59^.

### Scanning electron microscopy and transmission electron microscopy of PPEF and BPVF-treated bacterial cells

The bacterial cells were cultured in Luria-Bertani (LB) broths at 37 °C to an OD_600_ of 0.4. To observe the effects of treated cells, the compounds were incubated at 160 rpm for 4.5 h at 37 °C. Bacterial cells were cultured to an OD_600_ of 0.4, followed by treatment with BPVF for 4.5 h to their respective MIC concentration (1 µg/mL for *S. aureus* and 8 µg/mL for *E. coli*). Untreated controls were prepared under identical conditions. After centrifugation at 7000 rpm for 5 min, cell pellets obtained were rinsed thrice with PBS and then fixed with 2.5% glutaraldehyde in 1XPBS buffer (pH 7.2) for 2 h. After washing with PBS, cells were dehydrated in a graded series of chilled acetone in increasing concentrations (50, 70, 90, 95, and 100% v/v) at every 2 h interval. However, after treatment with 70% acetone, samples were left in it overnight at 4 °C, with further dehydration continued the following day. After the final dehydration step with 100% acetone, specimens were mounted on slides, dried under a vacuum, and SEM was performed with a Zeiss EVO40 microscope.

The sample preparation for TEM is similar to SEM except before starting the dehydration of samples, *E. coli* (MG1655) and *S. aureus* (ATCC43300) cells were again fixed with 1% Osmium tetraoxide (OsO4) for 2 h in the same buffer. After the final dehydration step with 100% acetone, cells were treated with toluene (Merck Millipore) for 2 h and then with epoxy resin. Three changes of pure epoxy resin were given at every 2 h interval. The pelleted cells embedded completely in epoxy resin were polymerized for 2–3 days at 60 °C until they solidified completely and were ready for fine sectioning. Ultrathin sectioning of pellets was done by Ultramicrotome, Leica EM UC 6, and Nikon Trinocular Microscope with a digital camera (Model E200). Ultrathin sections were placed on copper grids, stained for 5 min in aqueous uranyl acetate, and air-dried before imaging. Microscopic imaging was performed with a JEOL-2100F (JEOL Ltd., Tokyo, Japan) operated under a 200 kV acceleration voltage^59^.

### Time-kill assay

To evaluate the effect of PPEF and BPVF on bacterial growth, a time-response growth curve was prepared based on the NCCLS standards^60^. The 1 mL bacterial suspensions at a cell density of 10^7^ CFU mL^−1^ were exposed to BPVF. In the control tube, an equal volume of sterile Mili-Q water was added. These cultures were incubated at 37 °C with constant stirring at 200 rpm. Broth aliquots were collected at different time points, serially diluted in saline solution, plated on Müller-Hinton agar media, and grown for 24 h at 37 °C to determine the total CFUs in each culture at respective time points. Bactericidal activity is defined as a greater than 3 log10-fold decrease in colony-forming units (surviving bacteria), which is equivalent to 99.9% killing of the inoculum.

### Resistance studies

Bacterial cells {*E. coli* (MG1655) and *S. aureus* (ATCC43300)} cells were grown overnight, then diluted to fresh media and grown till OD 0.6, then, washed once with physiological saline, and resuspended in saline to a final concentration of 10^10^ to 10^11^ CFU/ml. An aliquot (100 µl) of bacterial suspension was spread onto plates of cation-adjusted Mueller-Hinton agar containing the PPEF, and levofloxacin at two, five, and ten times the agar dilution MIC. The plates were incubated aerobically at 37 °C for 48 h. Resistance studies were defined as a MIC ≥4 times higher than that of the parent. Resistance frequencies for each MIC for each strain/antibiotic pair were calculated as the proportion of resistant colonies per inoculum^61^.

Resistance development done by sequential passaging *S. aureus* ATCC 25923 and ATCC4330 cells at exponential phase, was diluted to an A_600nm_ (OD_600_) of 0.01 in 1 ml of MHB with PPEF. Cells were incubated at 37 ˚C with agitation and passaged at 24 h intervals in the presence of PPEF at increasing concentration. This population was maintained through the transfer of 1% population into fresh media and compound every 24 h for 55 transfers resulting in ~8 generations per day total of 440 generations^62^. The MIC was determined by broth microdilution. Experiments were performed with biological replicates.

### Accumulation and efflux kinetics of PPEF and BPVF

The strains, MG1655 is *E. coli* (wild-type), and mutations in *lpxC* (lpxC1272) and lpxD (lpxD14) lowered the lipopolysaccharide (LPS) levels in the wild-type, whereas inactivation of *yciM* leads to high LPS levels in the wild-type were used in this study. The OD_600_ of all bacterial cultures (*E. coli* MG1655, *ΔyciM, LpxC1272, LpxD14*, *S. aureus* ATCC 25923, and *A. baumannii* W407) suspensions were adjusted to 0.1 and aliquot 180 μL were transferred to wells (Corning® 96 Well Black with Clear Flat Bottom). The 2.5 μM of BPVF and PPEF were added to each well. Fluorescence was read from the wells’ top using excitation and emission filters of 345 and 475 nm, respectively, with five flashes/well; readings were taken for 120 cycles with a 60 s delay between cycles using Tecan Infinite Pro 200 Reader^24^. Each experiment was repeated thrice.

The cultures were treated with PPEF and BPVF (1 × MIC) for 1 h, and the bacterial suspension was centrifuged at 4000×*g* for 3 min. The pellet was resuspended in PBS, adjusting the OD_600_ to 0.1, and aliquots of 180 μL were transferred to wells (Corning® 96 Well Black with Clear Flat Bottom). The efflux pattern of molecules was observed by fluorescence spectrometry, as mentioned above. Each experiment was repeated thrice^24^.

### Antibiofilm assay

The biofilm assay was conducted in a 96-well polystyrene flat-bottomed microplate. Firstly, the standardized *S. aureus* (ATCC 43300) suspension at 10^6^ cfu/mL was prepared and dispensed into each well in the 96-well plate. For the dose-dependent antibiofilm assay, PPEF, BPVF, and CIP were tested across of series of concentrations, ranging from 0.025 to 1 µg/mL. Uninoculated MH broth was also included as the blank control. The plate was incubated at 37 °C for 24 h. After incubation, the wells were washed three times with 1× phosphate-buffered saline (PBS) to eliminate non-adherent bacteria and fixed with 99% (v/v) methanol for 15 min. The wells were allowed to dry in a laminar flow. The attached biofilm cells were stained using filtered 0.5% crystal violet for 15 min at room temperature. The excess stain was removed by rinsing with water, and crystal violet-bound cells were solubilized with 70% ethanol. The released stain was measured at 570 nm using a microplate reader (Tecan)^18^. Analysis was performed on three independent occasions and three technical replicates for each.

### Pharmacokinetic study of PPEF in BALB/c mice

Blood sampling and plasma sample extraction, intravenous pharmacokinetics in non-fasted state of animals was examined at a dose of 40 and 10 mg/kg bw, PPEF, and BPVF salt dissolved in sterile water, respectively. BALB/c male mice (5 groups, 3 animals/group, males) aged 8–9 weeks were used. Animals were distributed such that the body weight variation of animals selected for the study does not exceed ±20% of the mean body weight. Blood samples were collected at 0.0, 0.08, 0.25, 0.5, 1, 2, 4, 6, 8, and 24 h after intravenous administration of the drug. Blood specimens were collected into pre-labeled tubes containing an anticoagulant (K_2_EDTA-2 mg/mL blood) during the next 24 h of post-dose. Collected blood specimens were centrifuged at 4000 rpm for 10 min at 4 °C and plasma was separated and stored at −80 °C until analysis was determined using the LC-MS system. Calibration curve standards were prepared in a range between 2.349, 2.936, 5.873, 11.746, 23.492, 46.984, 93.967, 187.934, 375.868, 751.736, 1503.472, 3006.945, 6013.890, 12027.780, and 15034.724 ng/mL in plasma by spiking blank plasma with aqueous analyte standards. Exactly 10 μL of Haloperidol as an internal standard drug working solution (5 μg/mL) were added into pre-labeled 1.5 mL tubes. To that same tube, 50 μL of the study sample (plasma) was added. Exactly 0.15 mL of Methanol was added to all the tubes and vortexed for 30 s. Samples were centrifuged for 10 min at 4000 rpm at a set temperature of 4 °C. About 0.125 mL of supernatant was transferred into pre-labeled HPLC vials and placed into the autosampler at 15 °C. The 10 μL of the sample was injected into the LC-MS/MS instrument for analysis^63^.

Chromatographic conditions, API 3200 Q Trap system, and Zorbax SB C18 (4.6 mm × 50 mm, 3.5 μm) were used to quantify the blood concentration of PPEF and BPVF. The mobile phase was composed of Methanol: 0.1% Formic acid (90:10 v/v). The flow rate was 0.900 mL/min. The column temperature was set to 15 °C. The data of plasma concentrations at respective time points for the test item PPEF and BPVF were used for the pharmacokinetic analysis. Pharmacokinetic analysis was performed using the non-compartmental analysis (NCA) module of Phoenix WinNonlin 6.3 software to determine the pharmacokinetic parameters.

### Acute toxicity study in mice

The acute toxicity study was done as per the OECD Test No. 423 guidelines^64^ for testing chemicals. Briefly, female mice (nulliparous and nonpregnant; 23–25 g bw) were divided into six groups containing six animals each. Control group 1 received saline at a dose volume of less than 10 ml/kg bw; the test groups 2, 3, 4, and 5 received a single bolus dose of 500, 1000, 1500, and 2000 mg/kg bw of PPEF and BPVF by oral gavage. Mice weighing 25 g received a PPEF and BPVF concentration of 12.5, 25, 37.5, and 50 mg dissolved in 0.2 ml of sterile water for 500, 1000, 1500, and 2000 mg/kg bw doses. The animals were observed for 15 to 30 min after dosing for any physical symptoms and then checked every 2 to 3 h for any physical changes in the skin, fur, eyes, and tail and were also observed for their behavioral changes. The intravenous dose of 50, 100, and 125 mg/kg bw was given, and the animals were observed for mortality for 15 days to learn the lethal dose of PPEF and BPVF.

### In vitro cytotoxicity assay

A cytotoxicity evaluation of PPEF and BPVF was performed using a 3-(4,5-Dimethylthiazol-2-yl)-2,5 diphenyltetrazolium bromide (MTT) assay^65^. Briefly, A549, HEK, HEPG2, and NIH3T3 cells were harvested, counted, and seeded at 3 × 10^3^ cells per well in 96-well plates for 24 h. The following day, the cells were treated with various concentrations of PPEF and BPVF. The cells were incubated for 24, 48, and 72 h at 37 °C with 5% CO_2_. After that, 0.5 mg/mL of MTT was added to each well and incubated for 4 h. Next, the solution was removed and 150 µl of dimethyl sulfoxide (DMSO) was added to the wells. Then, the plate was read at 575 nm by an infinite M200 pro. The results were analyzed as the percentage of viable cells to the concentration of PPEF and BPVF.

### Hemolysis assay

The human erythrocyte toxicity assays were performed as described earlier^32^. Human blood was collected from the healthy volunteer. Red blood cells (RBCs) were centrifuged at room temperature to 1400 rpm for 10 min. The supernatant was discarded, and RBCs were recovered into a 1×PBS buffer. RBCs were centrifuged again, and the pellets were resuspended in 1× PBS. RBCs were washed until the supernatant was clear of that suspension, and 75 μl was mixed with 75 μl of twofold serial dilutions of BPVF, PPEF, and ciprofloxacin (CIP) in a microtiter plate, from 0.25 to 128 mg/L in 1× PBS. The plates were incubated for 2 h at 37 °C. After plate centrifugation (15 min, 1400 rpm), the absorbance of the supernatant was measured at 414 nm. A solution of water with 1% Triton X-100 was used as a positive control with all RBCs lysed, and the hemoglobin release was set at 100%. RBC in PBS was used as a negative control without hemolysis.

### Animal efficacy studies

All experiments were performed according to the CPCSEA guidelines in Central Laboratory Animal Resources (CLAR), followed by the approval of the institutional animal ethics committee (IAEC). The animals were housed randomly in non-ventilated cages, containing the paddy husk as bedding. Six to 8 weeks old male and female mice were maintained in a controlled 12 h day and night cycle with regulated temperature (23 ± 2 °C) and humidity (50 ± 5%). We used 6 to 8 weeks old male and female mice, ~25 ± 5 g, for all the experiments. The ARRIVE guidelines were followed thoroughly.

### In vivo antisepsis activity

Female Balb/c mice *n* = 6 per group, weighing 20–25 g of 6–8 weeks old, were infected with *E. coli* ATCC 25922 (0.5 × 10^8^ CFU in 0.1 mL saline) intraperitoneally which was observed to be 90 to 100% lethal. At 2.0 h post-infection, PPEF at different doses (3, 5, and 7 mg/kg BW) and BPVF (4, 8, and 12 mg/kg BW) dissolved in sterile water as vehicle at a volume of 0.1 mL given a single intravenous injection in the tail vein, whereas control animal received vehicle by the same mode of administration. Mice were observed for infection-lead mortality for 15 days post-infection. The effective dose 50 (ED_50_) was determined as the dose that lead to the survival of 50% of mice in the population. Mice survival was plotted by Kaplan–Meier plot and log-rank test (Mantel–Cox test) was performed to calculate significance <0.05 was considered significant.

Similarly, mice were injected intraperitoneally (i.p.) with the cell suspension of MRSA (ATCC 43300) containing ~2 × 10^9^ CFUs in 0.1 mL saline(100× LD_50_ value of bacteria). In order to determine the ED_50_ of PPEF and BPVF, the septicemia mice group were injected with two different doses of 10 and 20 mg/kg BW 2h post-infection and two groups of mice were given two standard antibiotics, ciprofloxacin and levofloxacin for comparison. The survival of all four groups was monitored for 15 days. In a different follow-up study, in order to see the effect of accumulated dose, three doses (3 × 10 mg/kg BW) of BPVF and ciprofloxacin were given via i.v. mode after 2, 24, and 48 h, post infections to the septicemic mice (*n* = 6 in each group). Mice survival was monitored for 15 days and the ED_50_ value was calculated by Probit analysis^66^.

### Efficacy of PPEF and BPVF in neutropenic thigh model

The neutropenic thigh infection model, fully described by ref. ^63^, was used in this study. Male and female Swiss Albino mice (five mice per dosing group) weighing 20–25 g were rendered neutropenic with two intraperitoneal injections of cyclophosphamide (150 mg/kg of bw 4 days before bacterial inoculation and 100 mg/kg 1 day before inoculation), 2 h before bacterial inoculation^67^. This regimen reliably resulted in transient neutropenia in mice that lasted for at least 3 days after the last dose of cyclophosphamide was given. Bacteria (ATCC 43300 and S1016) were injected into the right thigh of each mouse at time zero. Inocula were selected based on pilot studies with vehicle-treated animals that determined the maximum number of CFU that could be inoculated without substantial mortality. Neutropenia was defined as an absolute neutrophil count of 500 polymorphonuclear leukocytes/cm^3^ of blood. The bacterial suspension was diluted to a concentration of 10^9^ CFU/mL with normal saline. Then, 0.1 mL of the bacterial suspension was injected into each posterior thigh muscle 2 h after the second dose of cyclophosphamide was administered. The bacteria-infected mice were given PPEF, BPVF, levofloxacin, and ciprofloxacin at different doses (10 and 20 mg/kg bw) by dissolving in sterile water as a vehicle at a volume of 0.1 mL using a single bolus intravenous injection in the tail vein 2 h after bacterial inoculation. The mice were humanely sacrificed after 24 h of drug treatment. Right thigh muscles from each mouse were aseptically collected, homogenized, and serially diluted 1:10 in phosphate buffer saline, and processed for quantitative cultures.

### Organ-specific clearance of infection in PPEF-treated mice

Mice were infected by intraperitoneal administration of 10^9^ CFU MRSA (ATCC 43300) in 0.1 mL. After bacterial challenge for 2 h, mice were randomized to receive an intraperitoneal injection of saline as a control, PPEF (10 and 20 mg/kg), dosages. To assess bacterial clearance, ten mice (male and female) in each group were euthanized, and bacterial counts were determined in the liver, lung, kidney, and spleen of each animal after the bacterial challenge for 24 h.

### Statistics and reproducibility

All the experimental data were expressed as mean ± SD for at least three independent experiments (*n* = 3) unless otherwise stated. The Kaplan–Meier estimator was used to generate survival curves and to estimate the median survival in mice models. Statistical significance was estimated using GraphPad Prism 8. The results were assayed through either one-way or two-way ANOVA followed by Tukey’s multiple comparison post hoc analysis. The experiment with two parameters was compared using a *t*-test. Data were analyzed using either one-way or two-way ANOVA followed by Tukey’s multiple comparison post hoc analysis. The *p* value * < 0.05, ** < 0.005, *** < 0.0005, and **** < 0.00005 were considered as significant.

### Reporting summary

Further information on research design is available in the Nature Portfolio Reporting Summary linked to this article.

## Supplementary information

Supplementary Information-New

Description of additional supplementary files

Supplementary Data 1

Supplementary Data 2

Supplementary Data 3

Supplementary Data 4

Supplementary Data 5

Supplementary Data 6

Supplementary Data 7

Supplementary Movie 1

Supplementary Movie 2

Supplementary Movie 3

Supplementary Movie 4

Supplementary Movie 5

Supplementary Movie 6

Reporting Summary

## Data Availability

All data that generated or support the findings of this study are included in this article and its supplementary information. Full-length uncropped original gel images for cleavage and relaxation used in the manuscript are shown in Supplementary Fig. 15. The source data that make up the all graphs in the paper are shown in Supplementary Data 1–7. Any further data of this manuscript will be available from the corresponding author upon reasonable request.
