# Supplementary material for: Unraveling topoisomerase IA gate dynamics in presence of PPEF and its preclinical evaluation against multidrug-resistant pathogens
Source: Commun Biol. 2023 Feb 18;6:195. doi: 10.1038/s42003-023-04412-1 (PMC9938908; doi:10.1038/s42003-023-04412-1)
Supplement: Supplementary file 1 — Supplementary Information-New [file 42003_2023_4412_MOESM1_ESM.pdf]

Supplementary information

Unraveling topoisomerase IA gate dynamics in presence of PPEF and its preclinical evaluation against multidrug-resistant pathogens

Vikas Maurya[1,#], Raja Singh[1,#], Reman Kumar Singh[2,#], Stuti Pandey[3], Pooja Yadav[1], Palak Parashar[1], Rajni Gaind[4], Kshatresh Dutta Dubey[5], G. Naresh Patwari[6], Vibha Tandon[1,*]

[1]Special Centre for Molecular Medicine, Jawaharlal Nehru University, New Delhi-110067, India.

[2]St. Xavier's College, Ranchi, Jharkhand-843001, India.

[3]Chemical Biology laboratory, Department of Chemistry, University of Delhi, Delhi-110007, India.

[4]Vardhaman Medical College Hospital, Safdarjung Hospital, New Delhi-110029, India.

[5]Department of Chemistry, School of Natural Sciences, Shiv Nadar University, Gautam Buddha Nagar Uttar Pradesh-201314, India.

[6]Department of Chemistry, IIT Bombay, Powai, Mumbai, Maharashtra-400076, India.

[#] These authors contributed equally.

*Corresponding author

Tel: 91-11-26742181

Email: vtandon@mail.jnu.ac.in

Supplementary Figures

The synthesis of PPEF was published earlier by our laboratory. The characterization of PPEF new batch is given below.

Supplementary Note 1. Characterization of PPEF

2'-(4-ethoxyphenyl)-5-(4-propylpiperazin-1-yl)-1H,1'H-2,5'-bibenzo[d]imidazole (PPEF)

Yellow solid; (48% yield, 0.49g); mp 205.2−206.2ºC. 1H NMR (400 MHz, DMSO−d6) 0.81 (t, *J* = 7.2 Hz, 3H), 1.29 (t, *J* = 6.8 Hz, 3H), 1.45 (dd, *J* = 14.8, 7.3 Hz, 2H), 2.40 – 2.32 (m, 2H), 2.63 (s, 4H),, 3.10 (s, 4H), 4.05 (dd, *J* = 13.8, 6.9 Hz, 2H), 6.86 (d, *J* = 8.8 Hz, 1H), 6.96 (s, 1H), 7.04 (d, *J* = 8.5 Hz, 2H), 7.38 (d, *J* = 8.7 Hz, 1H), 7.61 (d, *J* = 8.4 Hz, 1H), 7.96 (d, *J* = 8.4 Hz, 1H), 8.10 (d, *J* = 8.6 Hz, 2H), 8.15 (s, 1H), δ 8.25 (s, 1H),). 13C NMR (100 MHz, DMSO−d6) _ ppm 11.49, 14.9, 17.0, 46.3, 50.8, 57.2, 64.4, 99.0, 113.9, 115.17, 115.9,120.37, 126.34, 131.28, 133.4, 147.2,149.2, 151.6,163.3 FTIR (KBr, cm–1): 3151.43, 2960.43, 2928.41,2821.53, 2362.41, 1610.81, 1445.77, 1252.41, 1178.95, 1041.69, 963.13, 810.19,631.80.HRMS (ESI): m/z calcd for C29H32N6O [M + H]+481.2716obsd 481.2707.

Supplementary Note 2. Chemical Synthesis of BPVF

1. Synthesis of 2-(3,4-dimethoxyphenyl)-1H-benzo[d]imidazole-6-carbonitrile

Synthesis of 2-(3,4-dimethoxyphenyl)-1H-benzo[d]imidazole-6-carbonitrile was done using the reported procedure [22]To freshly prepared ethanolic solution of 4−cyano−1,2−phenylenediamine 6(7.51mmol, 1.0 equiv), react with the mixture of 3,4-dimethoxybenzaldehyde (1.5 equiv.) and solution of $Na_2S_2O_5$ (0.5 equiv) in water (1ml/100mg) were added. The resulting solution was stirred at reflux for 4−6h, then cooled to room temperature and filtered through a celite bed. The solvents were evaporated under the reduced pressure. The crude residue was purified by chromatography on silica gel (60−120 mesh size) in Ethyl acetate/Pet ether as solid in 70−80% yield.

2. 2-(3,4-dimethoxyphenyl)-1H-benzo[d]imidazole-6-carbaldehyde

Ni-Al alloy was added to a solution of in 75% aqueous formic acid. The reaction mixture was heated at 95ºC for 30min under an inert atmosphere. The hot mixture was filtered through celite bed followed by rinsing the reaction flask and the celite bed with water. The aqueous solution was concentrated to dryness. After addition of water to this residue, a precipitate was formed. The pH of this suspension was adjusted to 9.0 by the drop wise addition of 2N NaOH and the product was then extracted into ethylacetate. The organic layers were dried over anhydrous Na_2SO_4 and filtered. The solvents were evaporated under reduced pressure and further purified by chromatography on silica gel (60−120 mesh size) in MeOH/EtOAc as solid in 65−74% yield.

5-(4-butylpiperazin-1-yl)-2'-(4-ethoxyphenyl)-1*H*,1'*H*-2,5'-bibenzo[*d*] imidazole (BPVF)

5-(4-butylpiperazin-1-yl)-2'-(4-ethoxyphenyl)-1*H*,1'*H*-2,5'-bibenzo[*d*]imidazole synthesized was byused freshly prepared ethanolic solution of 4-(4-butylpiperazinyl)-1phenylene diamine (0.5g, 1.0 equiv) a mixture of 2-(3,4-dimethoxyphenyl)-1H-benzo[d]imidazole-6-carbaldehyde (1.5 equiv) and solution of $Na_2S_2O_5$ (0.5 equiv) in water (1mL/100mg) were added. The resulting solution was stirred at reflux for 24h, then cooled to room temperature and filtered through a bed of celite. The solvents were evaporated under reduced pressure. The crude residue was purified by chromatography on silica gel (100−200 mesh size) in MeOH/DCM as solid title compounds in 40−60% yield.

5-(4-butylpiperazin-1-yl)-2'-(4-ethoxyphenyl)-1*H*,1'*H*-2,5'-bibenzo[*d*] imidazole (BPVF)

Brown coloured crystalline solid, (82.6%% yield, 0.850g); mp 248−250.8 °C.^{1}H NMR (400 MHz, DMSO−d_6): δ ppm 0.89 (t, *J* = 7.3Hz, 3H),1.26-1.34 (m, 2H), 1.43-1.5 (m, 2H) 2.40 (t, *J* = 6.7Hz, 2H), 2.56-2.63 (m, 4H), 3.12-3.19 (m, 4H), 3.83 (s, 3H), 3.89 (s, 3H), 6.92 (dd, *J* = 8.56 Hz, 1.84 Hz, 1H), 7.01 (s, 1H), 7.14 (d, *J* = 8.5 ,1H), 7.43 (d, *J* = 8.5 Hz, 1H), 7.66 (d, *J* = 8.5 Hz, 1H), 7.70 (m, 1H) ,7.80 (s, 1H), 7.99 (d, *J* = 8.56 Hz, 1H), 8.16 (s, 2H), 8.27 (s, 1H); ^{13}C NMR (100 MHz, DMSO−$d_{6)}$:13.8, 20.0, 27.6, 49.4, 52.4, 57.2, 63.2, 113.2, 114.3, 120.3, 122.3, 124.3, 128.3, 147.2, 152.1, 153.0, 159.3, 164.0, 172.4; FTIR (KBr, cm^{-1}):

3390, 2959, 2835, 2361, 1607, 1498, 1437, 1264, 1227, 1177, 1137, 1097, 1021, 963, 831,871, 765, 720; HRMS (ESI): m/z calcd for $C_{30}H_{34}N_6O_2$ $[M + H]^+$511.2818, obsd 511.281.

Synthesis of 4-(4-butylpiperazinyl)-1phenylene diamine

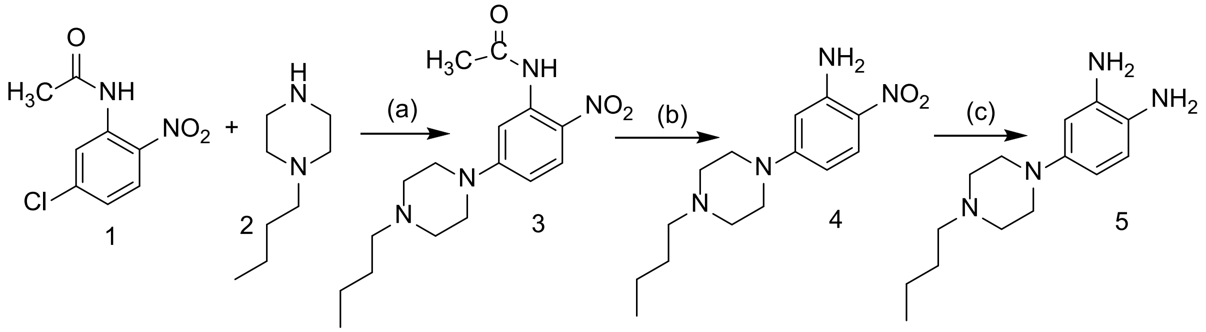

Supplementary Scheme 1: Reaction Condition: Reagents and conditions of 4-(4-butylpiperazinyl)-1phenylene diamine: (a) (Et)3N, DMSO, 120 °C; (b) 10% H2SO4, 80 °C; (c) 10% Pd/C,H2, EtOAc:MeOH (4:1)

Synthesis of 2-(3,4-dimethoxyphenyl)-1H-benzo[d]imidazole-6-carbaldehyde

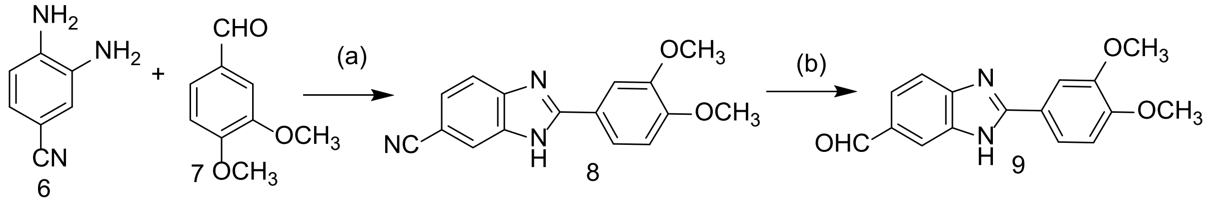

Supplementary Scheme 2: Reaction Condition: Reagents and conditions of 2-(3,4-dimethoxyphenyl) -1H-benzo[d]imidazole-6-carbaldehyde:(a) Na2S2O5 in water, ethanol, reflux; (b) Ni-Al alloy, 75% HCOOH, 95 °C

Synthesis of 5-(5-(4-butylpiperazin-1-yl)-1H-benzo[d]imidazol-2-yl)-2-(3,4-dimethoxyphenyl)-1H-benzo[d]imidazole

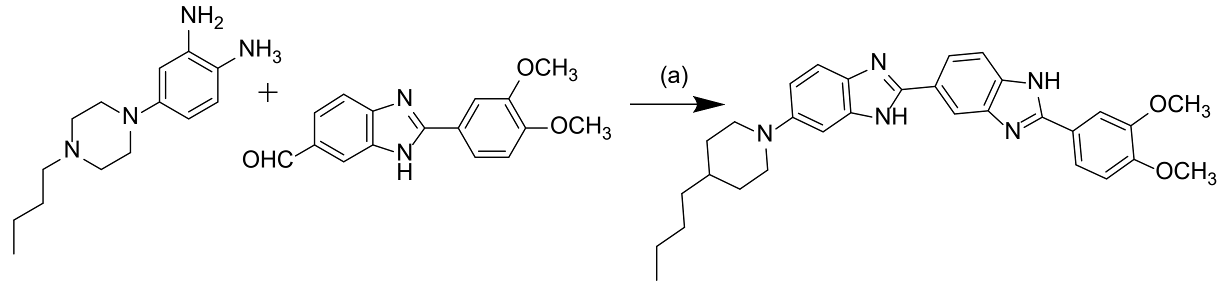

Supplementary Scheme 3: Reaction Condition: Reagents and conditions of 5-(5-(4-butylpiperazin-1-yl)-

1H-benzo[d]imidazol-2-yl)-2-(3,4-dimethoxyphenyl)-1H-benzo[d]imidazole (BPVF): (a) Na2S2O5 in water, ethanol, reflux.

a

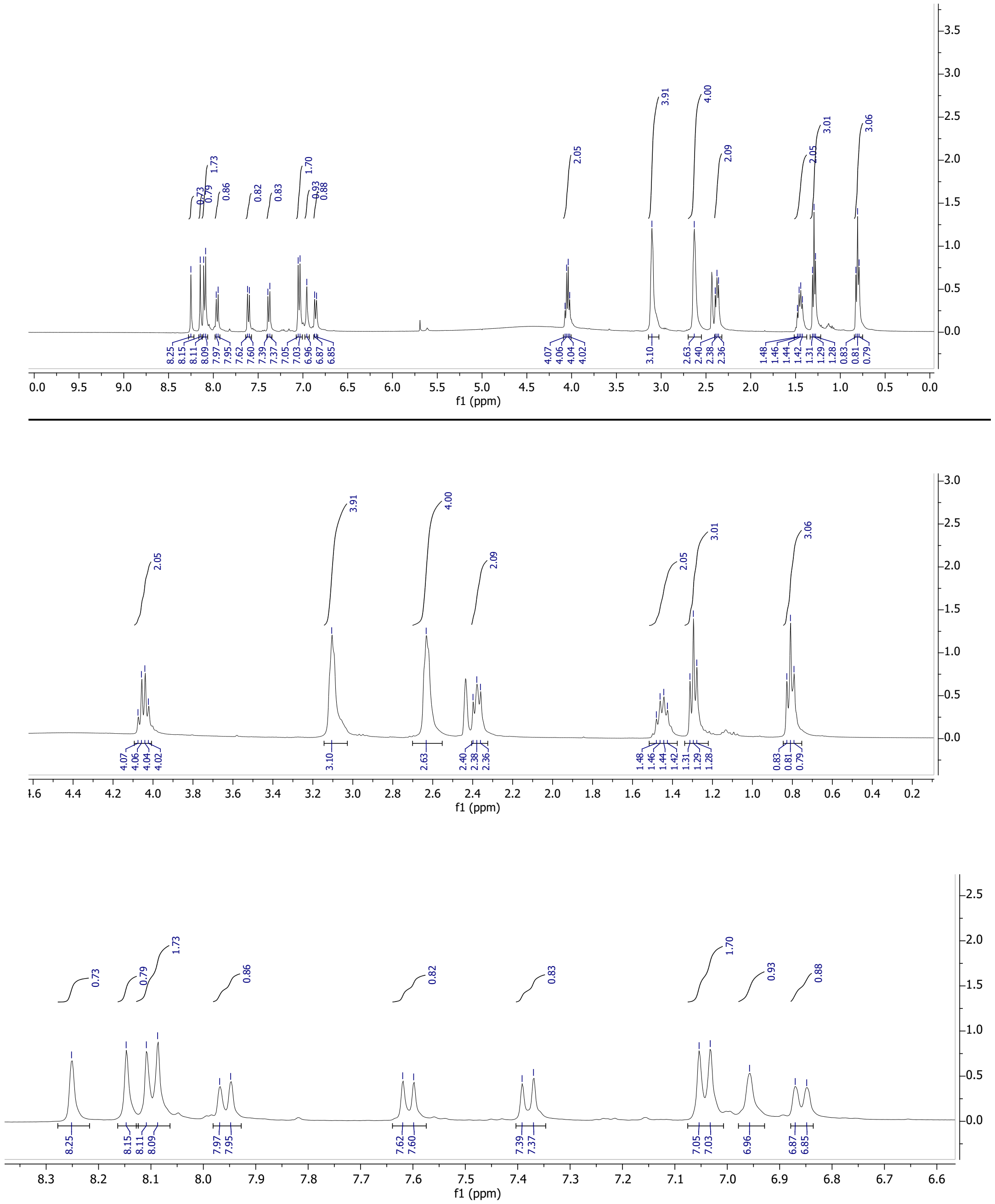

b

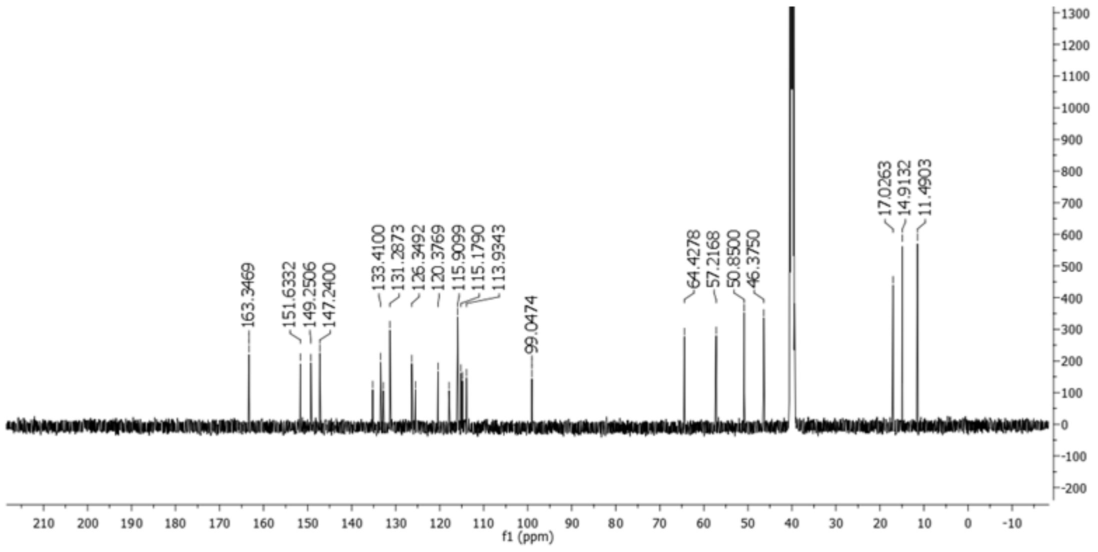
163.3469
151.6332
149.2506
147.2400
133.4100
131.2873
126.3492
120.3769
115.9099
115.1790
113.9343
99.0474
64.4278
57.2168
50.8500
46.3750
17.0263
14.9132
11.4903
f1 (ppm)

c

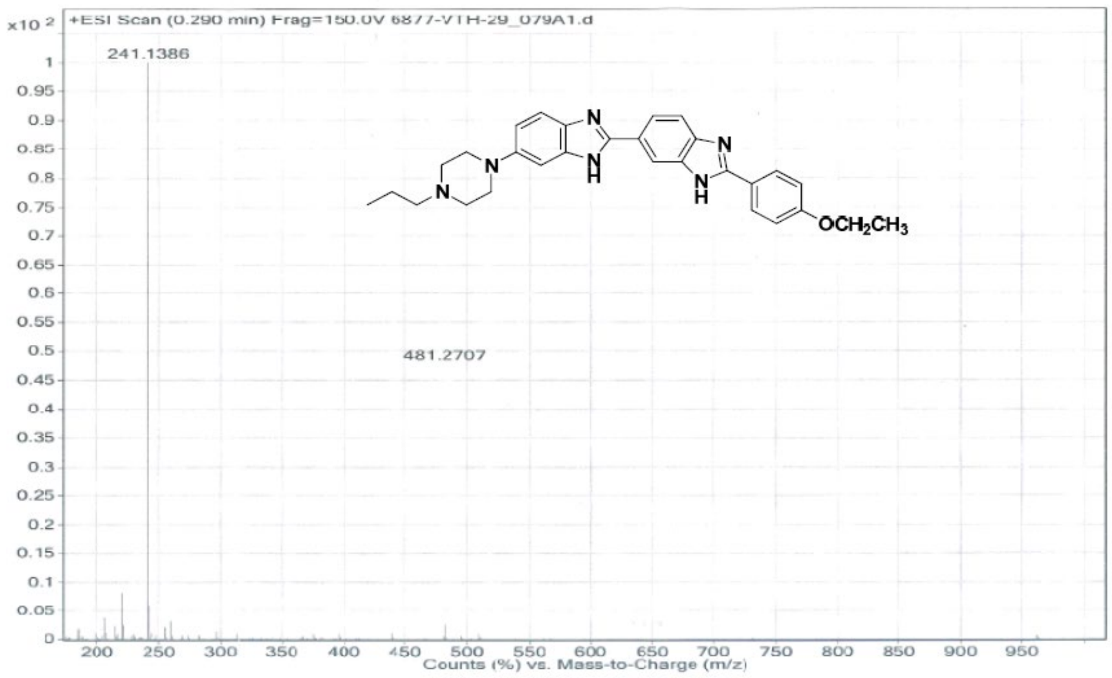
+ESI Scan (0.290 min) Frag=150.0V 6877-VTH-29_079A1.d
x10 2
241.1386
481.2707
OCH2CH3
Counts (%) vs. Mass-to-Charge (m/z)

d

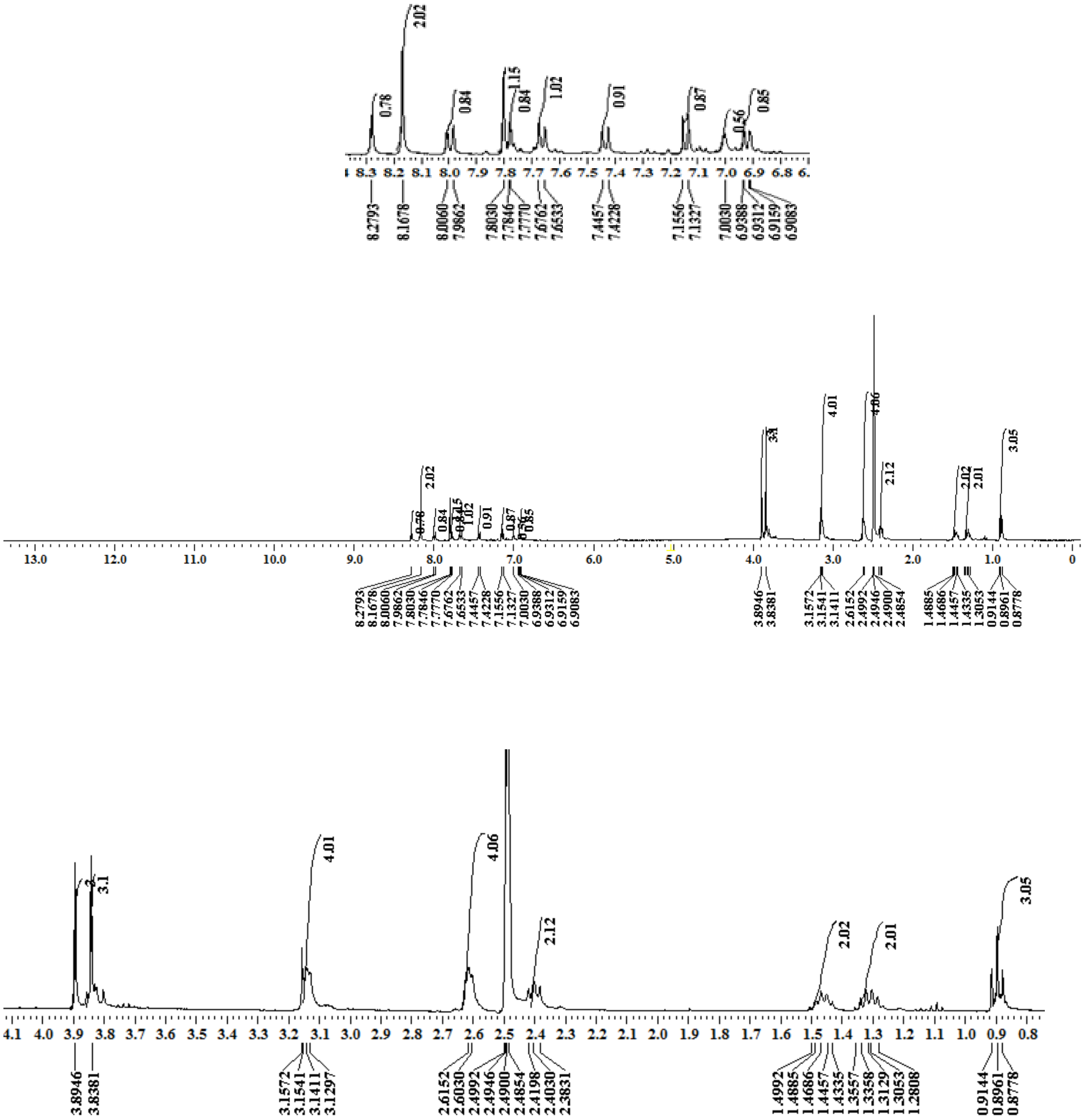
2.02
0.78
0.84
1.15
0.84
1.02
0.91
0.87
0.56
0.85
8.2793
8.1678
8.0060
7.9862
7.8030
7.7846
7.7770
7.6762
7.6533
7.4457
7.4228
7.1556
7.1327
7.0030
6.9388
6.9312
6.9159
6.9083
3.1
4.01
4.06
2.12
2.02
2.01
3.05
3.8946
3.8381
3.1572
3.1541
3.1411
3.1297
2.6152
2.6030
2.4992
2.4946
2.4900
2.4854
2.4198
2.4030
2.3831
1.4992
1.4885
1.4686
1.4457
1.4335
1.3557
1.3358
1.3129
1.3053
1.2808
0.9144
0.8961
0.8778

e

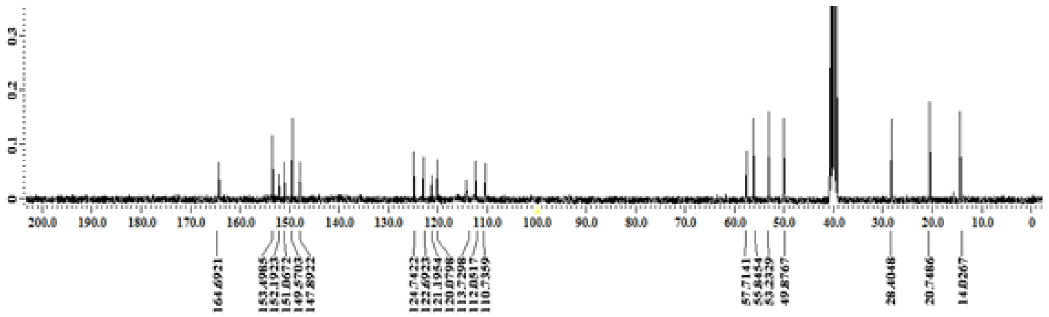
164.6921
153.4985
152.1923
151.0672
149.5703
147.8922
124.7422
122.6923
121.1954
120.0798
113.7298
112.0517
110.7359
57.7141
55.8454
53.2329
49.8767
28.4048
20.7486
14.0267

f

Compound Table

Compound Label	RT	Mass	Formula	MFG Formula	MFG Diff (ppm)	DB Formula
Cpd 10: C30 H34 N6 O2	0.193	510.2736	C30 H34 N6 O2	C30 H34 N6 O2	1.35	C30 H34 N6 O2

Compound Label	m/z	RT	Algorithm	Mass
Cpd 10: C30 H34 N6 O2	511.281	0.193	Find by Molecular Feature	510.2736

MFE MS Spectrum

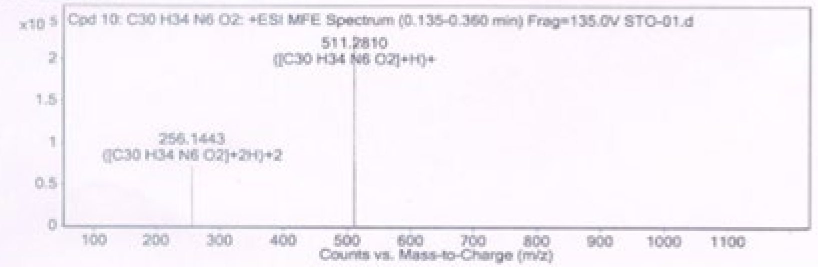

Supplementary Figure 1. NMR and HRMS identification of PPEF and BPVF. (a) ^{1}H NMR, (b) ^{13}C NMR, (c). HRMS of 2'-(4-ethoxyphenyl)-5-(4- propylpiperazin-1-yl)-1H,1'H-2,5'-bibenzo [d]imidazole (PPEF). (d) ^{1}H NMR, (e) ^{13}C NMR, (f) HRMS of 5-(4-butylpiperazin-1-yl)-2'-(3, 4-dimethoxyphenyl)-1*H*,1'*H* 2,5'bibenzo[*d*]imidazole (BPVF).

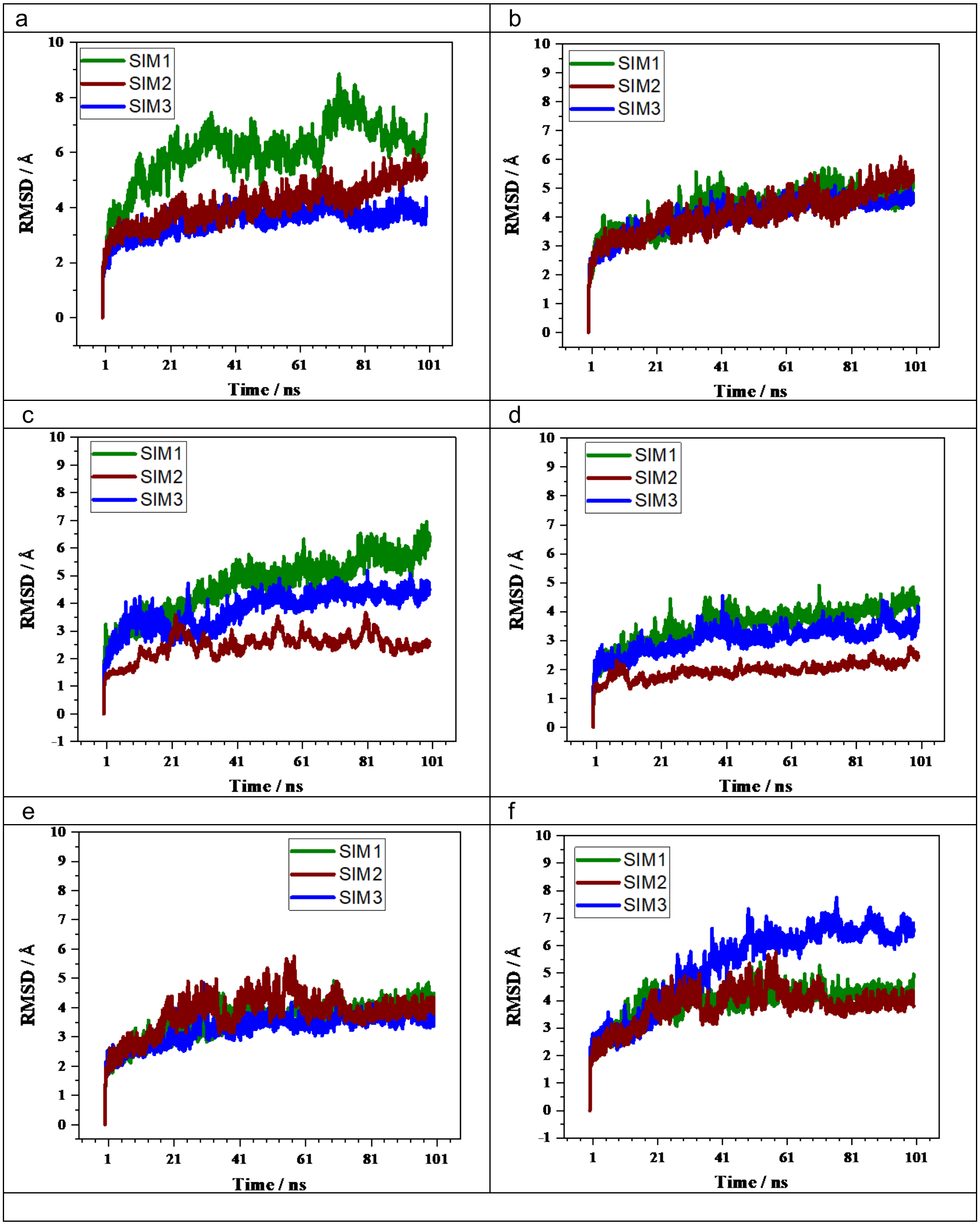

Supplementary Figure 2. RMSD of protein over the simulation time for all three independent simulation trajectory. (a) TopoIA (b) TopoIA-PPEF binary complex (c) TopoIA-dsDNA binary complex (d) TopoIA-ssDNA binary complex (e) TopoIA-dsDNA-PPEF ternary complex and (f) TopoIA-ssDNA-PPEF ternary complex.

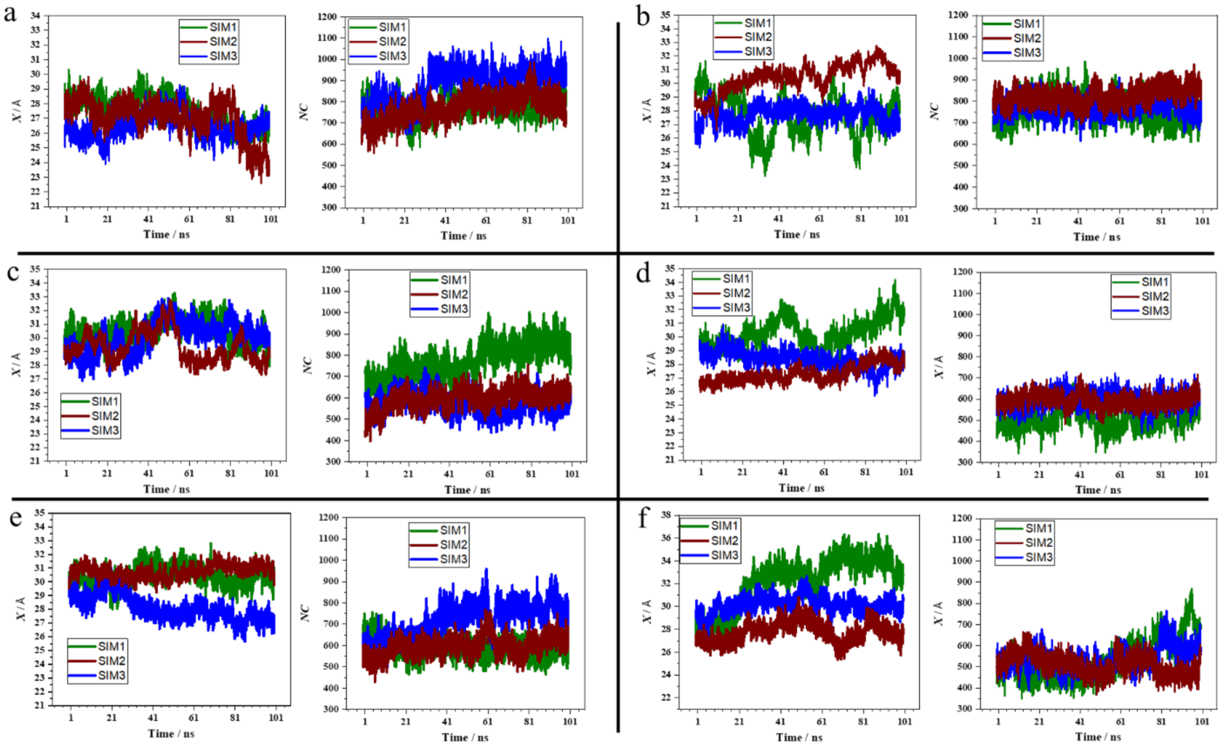

Supplementary Figure 3. Variation of *X* (Left panel) and *NC* (Right panel) along the molecular dynamic simulation for all three independent trajectory (named as SIM1, SIM2 and SIM3). (a) TopoIA (b) TopoIA and PPEF binary complex (c) TopoIA and dsDNA binary complex. (d) TopoIA and ssDNA binary complex. (e) TopoIA, dsDNA and PPEF ternary complex and (f) TopoIA, ssDNA and PPEF ternary complex.

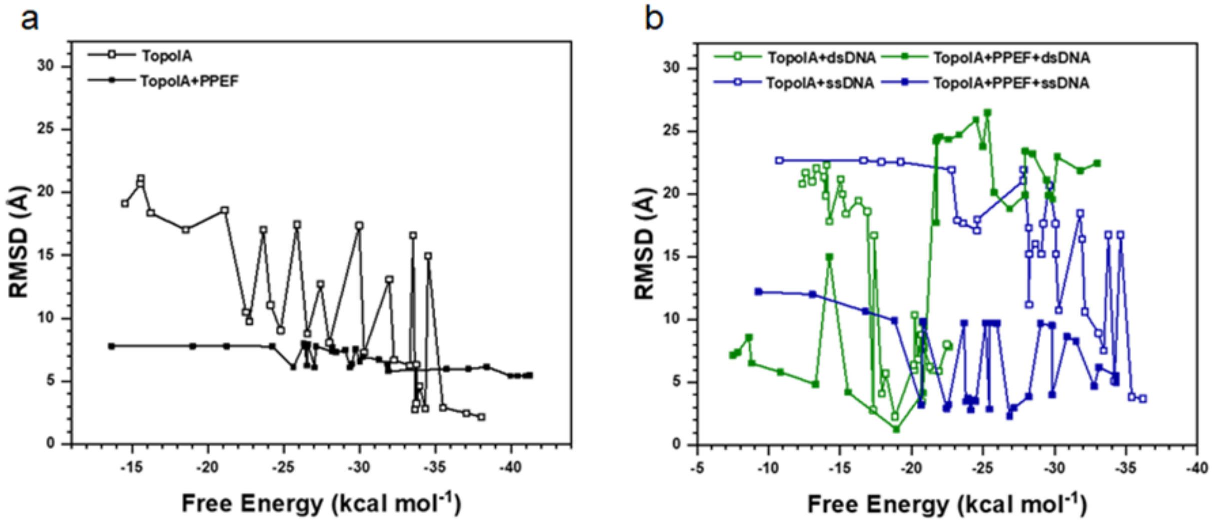

Supplementary Figure 4. Plots of RMSD against free energy for (a) topo IA (open squares) and PPEF bound topo IA (filled squares) and (Bb topo IA in the presence of dsDNA (green open squares), ssDNA

(blue open squares), and PPEF bound topo IA in the presence of dsDNA (green filled squares), ssDNA (blue filled squares) (filled squares). The RMSD is relative to the crystal structure of topoI.

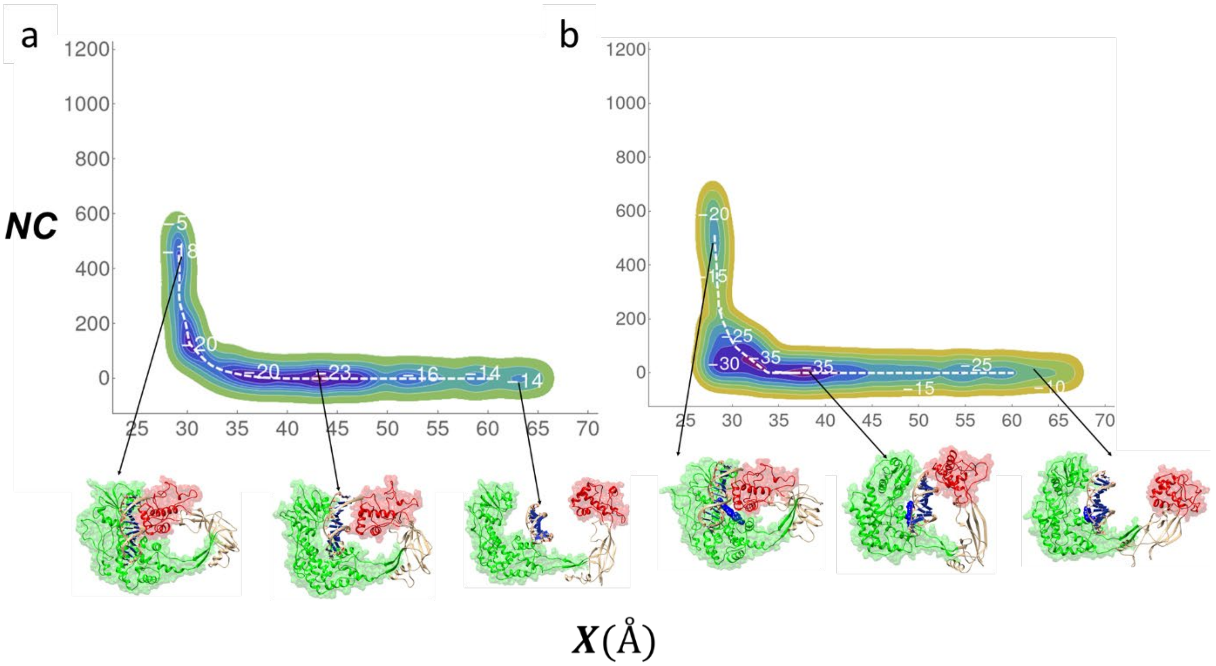

Supplementary Figure 5. Free energy surface of TopoIA close-open dynamics in presence of (a) dsDNA and (b) PPEF and dsDNA. The structure below each free energy surface represents the three states (close, partially open, and open) of TopoIA conformation.

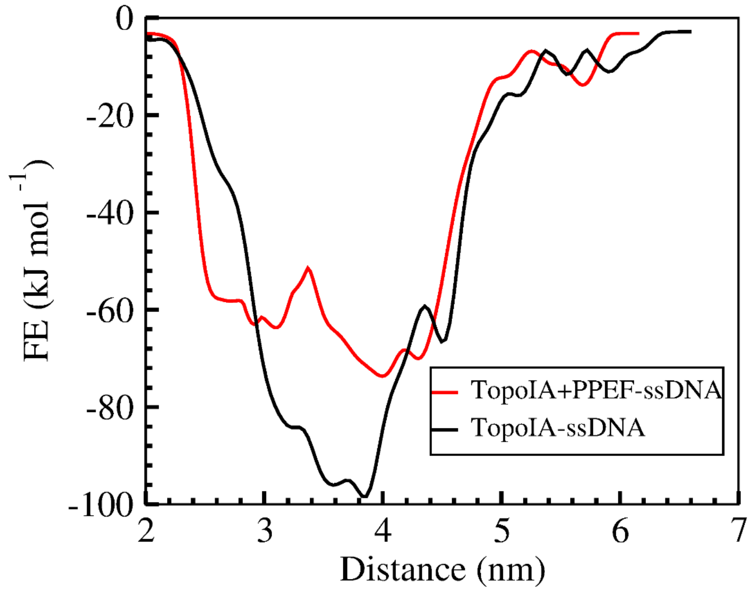

Supplementary Figure 6. Unbinding free energy of ssDNA from the TopoIA (black color) and TopoIA+PPEF complex (red color). The X-axis represent the distance between the centre of mass of TopoIA to the center of mass of ssDNA.

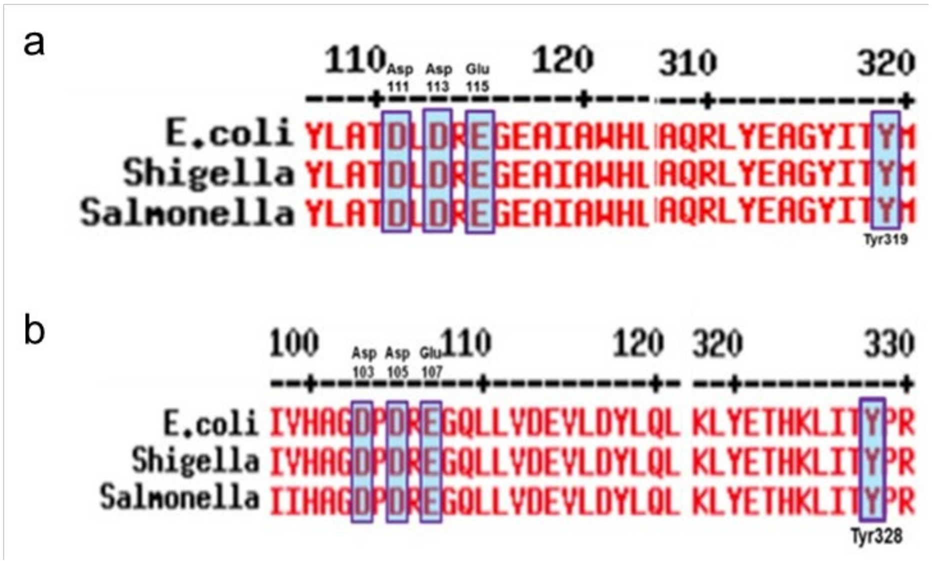

Supplementary Figure 7. Topoisomerase protein (active site) sequence alignment of *E. coli, S. flexneri,* and *Salmonella spp*. (a) Shows the active site similarity of three highly conserved acidic residues, EcTopoI (Asp111, Asp113, and Glu115), and the catalytic tyrosine of TopoI (Y319). (b) Showing the active site similarity of three highly conserved acidic residues EcTopoIII (Asp103, Asp105, and Glu107) and the catalytic tyrosine of TopoIII (Y328).

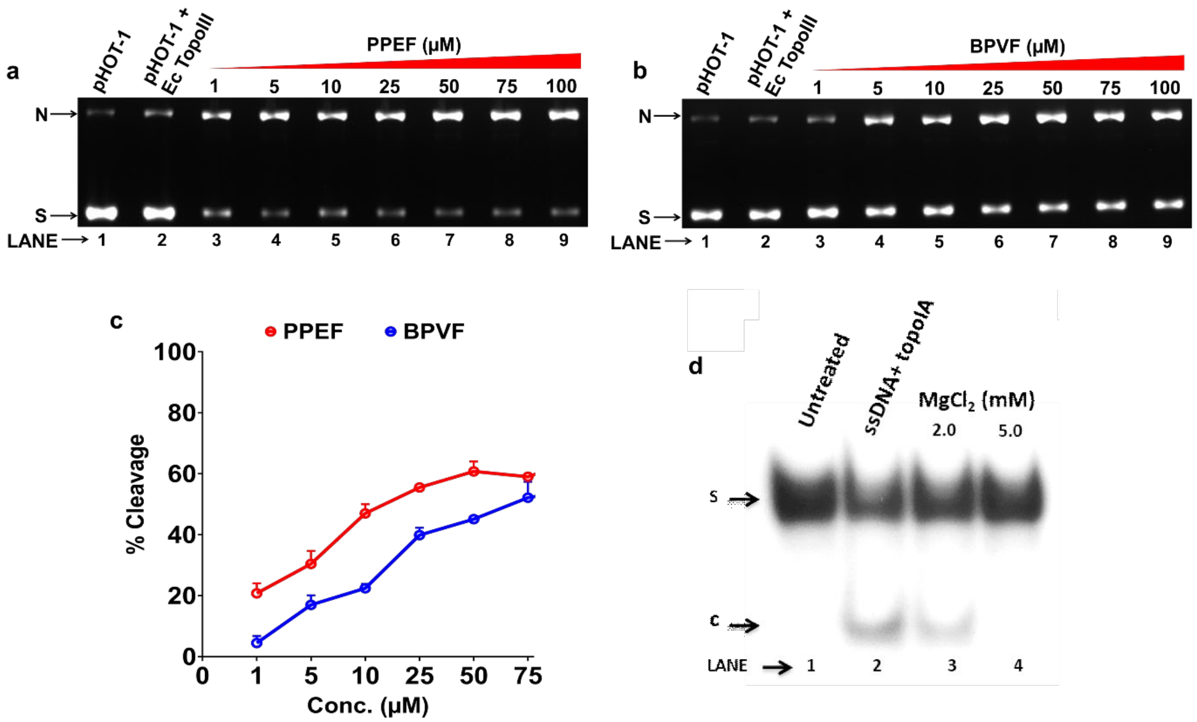

Supplementary Figure 8. EcTopoIII cleavage assay shows that cleavage is induced by PPEF and BPVF. (a) Lane 1- C: Control (350ng, pHOT1 plasmid DNA), Lane 2 Enzyme control (350ng, pHOT1 plasmid DNA + *E.coli* TopoIII), Lanes 3-9, Increasing concentration of PPEF (1, 5, 10, 15, 20, 25, 50, 75 µM). (b) Lane 1- C: Control (350ng, pHOT1 plasmid DNA), Lane 2 Enzyme control (350ng, pHOT1 plasmid DNA + *E.coli* TopoIII), Lanes 3-9, Increasing concentration of BPVF (1, 5, 10, 15, 20, 25, 50, 75 µM). (c) Percentage increase in the cleavage of plasmid DNA as an increase in the concentration of PPEF and BPVF respectively. (d) DNA cleavage by SaTopoIA, the addition of Mg2+ allows DNA religation to be re-established.

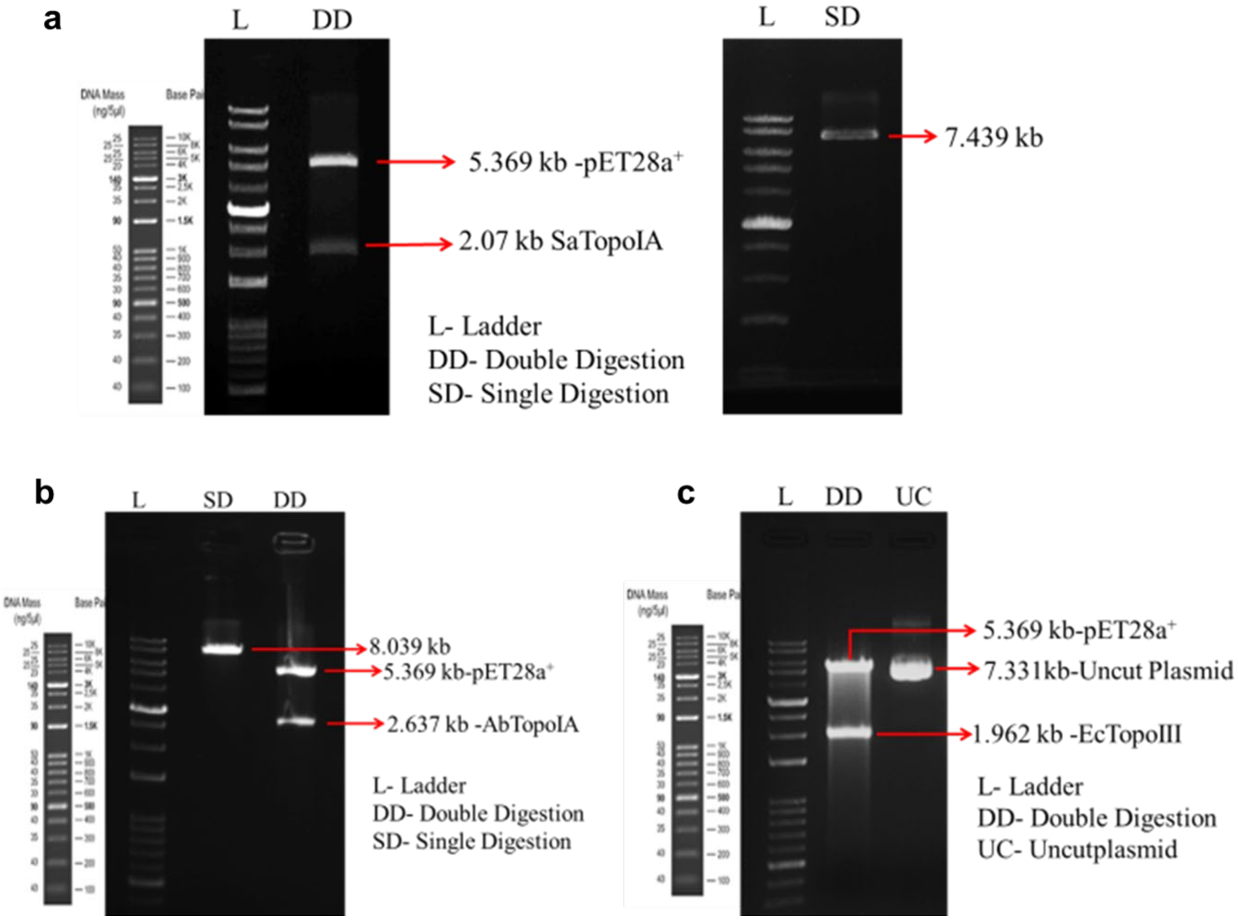

d

SaTopoIA Sequence Alignment

```
Query  83    TTACGAACCAAAATATATAACAATACGCGGAAAAGGTCCTGTTGTaaaagaattgaaaaa  142
             ||||||||||||||||||||||||||||||||||||||||||||| ||||||||||||||
Sbjct  147   TTACGAACCAAAATATATAACAATACGCGGAAAAGGTCCTGTTGTTAAAGAATTGAAAAA  206
Query  143   acatgcaaaaaaagcgaaaaaCGTCTTTCTCGCAAGTGACCCCGACCGTGAAGGTGAAGC  202
             ||||||||||||||||||||||||||||||||||||||||||||||||||||||||||||
Sbjct  207   ACATGCAAAAAAAGCGAAAAACGTCTTTCTCGCAAGTGACCCCGACCGTGAAGGTGAAGC  266
Query  203   AATTGCTTGGCATTTATCAAAAATTTTAGAGCTTGAAGATTCTAAAGAAAATCGCGTTGT  262
             ||||||||||||||||||||||||||||||||||||||||||||||||||||||||||||
Sbjct  267   AATTGCTTGGCATTTATCAAAAATTTTAGAGCTTGAAGATTCTAAAGAAAATCGCGTTGT  326
Query  263   TTTCAACGAAATAACTAAAGACGCTGTTAAAGAAAGTTTTAAAAATCCTAGAGAAATTGA  322
             ||||||||||||||||||||||||||||||||||||||||||||||||||||||||||||
Sbjct  327   TTTCAACGAAATAACTAAAGACGCTGTTAAAGAAAGTTTTAAAAATCCTAGAGAAATTGA  386
Query  323   AATGAACTTAGTCGATGCACAACAAGCGCGTCGAATATTAGATAGATTAGTTGGCTATAA  382
             ||||||||||||||||||||||||||||||||||||||||||||||||||||||||||||
Sbjct  387   AATGAACTTAGTCGATGCACAACAAGCGCGTCGAATATTAGATAGATTAGTTGGCTATAA  446
Query  383   CATCTCGCCAGTTCTATGGaaaaaagtaaaaaaaGGGTTGTCAGCGGGTCGAGTTCAATC  442
             ||||||||||||||||||||||||||||||||||||||||||||||||||||||||||||
Sbjct  447   CATCTCGCCAGTTCTATGGAAAAAAGTAAAAAAAGGGTTGTCAGCGGGTCGAGTTCAATC  506
Query  443   TGTTGCACTTCGTTTAGTCATTGACCGTGAAAATGAAATACGAAACTTTAAACCAGAAGA  502
             ||||||||||||||||||||||||||||||||||||||||||||||||||||||||||||
Sbjct  507   TGTTGCACTTCGTTTAGTCATTGACCGTGAAAATGAAATACGAAACTTTAAACCAGAAGA  566
Query  503   ATATTGGACTATTGAAGGAGAATTTAGATACaaaaaatcaaaatttcaatgctaaatttc  562
             ||||||||||||||||||||||||||||||||||||||||||| |||||||||||| |||
Sbjct  567   ATATTGGACTATTGAAGGAGAATTTAGATACAAAAAATCAAAA-TTCAATGCTAAA-TTC  624
Query  563   cttcattataaaaataaaccttttaaattaaaaacgaaaaaaGATGTTGAGAACATTACA  622
             ||||||||||||||||||||||||||||||||||||||||||||||||||||| ||||||
Sbjct  625   CTTCATTATAAAAATAAACCTTTTAAATTAAAAACGAAAAAAGATGTTGAGAAAATTACA  684
Query  623   GCTGCATTAGATGGAGATCCATTCAAAATTACAAACTTGACTaaaaaag-aaaaaCGCGT  681
             ||||||||||||||||||| |||| ||||||||||| |||||||||||| ||||||||||
Sbjct  685   GCTGCATTAGATGGAGATCAATTCGAAATTACAAACGTGACTAAAAAAGAAAAAACGCGT  744
```

SaTopoIA Sequence Alignment-1

```
Query  82    AATAGACTTAACTATCGCTTTTGTATTACGACAATCCGGGAAGTTTGAGCAAGCCATAAA  141
             ||||||||||||||||||||||||||||||||||||||||||||||||||||||||||||
Sbjct  1839  AATAGACTTAACTATCGCTTTTGTATTACGACAATCCGGGAAGTTTGAGCAAGCCATAAA  1780
Query  142   CTTACCATAGCGCCCCATTTTTATAACCATAGGAGAACCACAAACTTCACAGTCTTCACC  201
             ||||||||||||||||||||||||||||||||||||||||||||||||||||||||||||
Sbjct  1779  CTTACCATAGCGCCCCATTTTTATAACCATAGGAGAACCACAAACTTCACAGTCTTCACC  1720
Query  202   GGCTGGCTCATCTTTGATTTCAATCTTTTCCATCTCTTCTTCAGCACGTTCAACATCTTG  261
             ||||||||||||||||||||||||||||||||||||||||||||||||||||||||||||
Sbjct  1719  GGCTGGCTCATCTTTGATTTCAATCTTTTCCATCTCTTCTTCAGCACGTTCAACATCTTG  1660
Query  262   TTTAAAGCTACTAAAGAAACCGTCGATTACTTTTCTCCATGTAATGTCGCCTTCTGCAAT  321
             ||||||||||||||||||||||||||||||||||||||||||||||||||||||||||||
Sbjct  1659  TTTAAAGCTACTAAAGAAACCGTCGATTACTTTTCTCCATGTAATGTCGCCTTCTGCAAT  1600
Query  322   CTTATCAAGTAACGTTTCCATATTCACTGTGAATTCCACATCAATAATCTCTGGGAAGTA  381
             ||||||||||||||||||||||||||||||||||||||||||||||||||||||||||||
Sbjct  1599  CTTATCAAGTAACGTTTCCATATTCACTGTGAATTCCACATCAATAATCTCTGGGAAGTA  1540
Query  382   TTCTTTCACTTGTTCATGAACTATTTCTCCCAACTCAGTAGGAACAAAACGCTTACTTTC  441
             ||||||||||||||||||||||||||||||||||||||||||||||||||||||||||||
Sbjct  1539  TTCTTTCACTTGTTCATGAACTATTTCTCCCAACTCAGTAGGAACAAAACGCTTACTTTC  1480
Query  442   TAATTTGACATAGTTACGCTTTTGAATCGTATCTTTTGTCGGGGCATAAGTTGATGGTCG  501
             |||||||||||||||||||||||||||||||||| |||||||||||||||||||||||||
Sbjct  1479  TAATTTGACATAGTTACGCTTTTGAATCGTATCTATTGTCGGGGCATAAGTTGATGGTCG  1420
Query  502   CCCAATTTTTCAATTCTTCTAGTGTTTTTACTAAATCTCGCCTCAGTATATCTTTGGAGG  561
             ||||||||| |||||||||||||||||||||||| ||||||||||||||||||| |||||
Sbjct  1419  CCCAATTTT-CAATTCTTCTAGTGTTTTTACTAA-TCTCGCCTCAGTATATCTT-GGAGG  1363
```

AbTopoIA Sequence Alignment-1

```
Query  70       GTCGCCTGCCAAAGCGAAAACCATCAACAAATATTTAGGTTCGCAGTACATTGTTAAGTC  129
                ||||||||||||||||||||||||||||||||||||||||||||||||||||||||||||
Sbjct  3467866  GTCGCCTGCCAAAGCGAAAACCATCAACAAATATTTAGGTTCGCAGTACATTGTTAAGTC  3467807

Query  130      TTCTGTAGGTCACGTACGTGACTTGCCAACAGGTGGCAGTAAAGCAACAGAGAAAAAGCC  189
                ||||||||||||||||||||||||||||||||||||||||||||||||||||||||||||
Sbjct  3467806  TTCTGTAGGTCACGTACGTGACTTGCCAACAGGTGGCAGTAAAGCAACAGAGAAAAAGCC  3467747

Query  190      GGCTGCCCGGACAAAACTCACTGAGGCTGAAAAAGAACAAAAAGCGAATCAGGCTTTAAT  249
                ||||||||||||||||||||||||||||||||||||||||||||||||||||||||||||
Sbjct  3467746  GGCTGCCCGGACAAAACTCACTGAGGCTGAAAAAGAACAAAAAGCGAATCAGGCTTTAAT  3467687

Query  250      CAATCGTATGGGCGTCGATCCGGAACATGGATGGCAAGCGCATTACGAAGTTCTGCCTGG  309
                ||||||||||||||||||||||||||||||||||||||||||||||||||||||||||||
Sbjct  3467686  CAATCGTATGGGCGTCGATCCGGAACATGGATGGCAAGCGCATTACGAAGTTCTGCCTGG  3467627

Query  310      CAAAGAAAATGTTGTTGCTGAACTCAAGAAACTTGCTAAAGATGCAGATGCAATCTATCT  369
                ||||||||||||||||||||||||||||||||||||||||||||||||||||||||||||
Sbjct  3467626  CAAAGAAAATGTTGTTGCTGAACTCAAGAAACTTGCTAAAGATGCAGATGCAATCTATCT  3467567

Query  370      CGCAACGGACTTGGATAGAGAAGGGGAAGCAATCGCTTGGCACTTACGCGAAGTGATTGG  429
                ||||||||||||||||||||||||||||||||||||||||||||||||||||||||||||
Sbjct  3467566  CGCAACGGACTTGGATAGAGAAGGGGAAGCAATCGCTTGGCACTTACGCGAAGTGATTGG  3467507

Query  430      TGGTGACGATAGCCGTTATCATCGTGTGGTATTTAACGAAATTACTAAAAATGCCATTCA  489
                ||||||||||||||||||||||||||||||||||||||||||||||||||||||||||||
Sbjct  3467506  TGGTGACGATAGCCGTTATCATCGTGTGGTATTTAACGAAATTACTAAAAATGCCATTCA  3467447

Query  490      AGAAGCATTTAAACAGCCAACACGTCTCGACTTAAACCGTGTTAATGCACAACAAGCACG  549
                ||||||||||||||||||||||||||||||||||||||||||||||||||||||||||||
Sbjct  3467446  AGAAGCATTTAAACAGCCAACACGTCTCGACTTAAACCGTGTTAATGCACAACAAGCACG  3467387

Query  550      TCGTTTCTTGGACCGTGTAGTAGGCTTCATGGTTTCGCCATTATTATGGGGAAAAGATTG  609
                |||||||||||||||||||||||||||||||||||||||||||||||||| |||||||||
Sbjct  3467386  TCGTTTCTTGGACCGTGTAGTAGGCTTCATGGTTTCGCCATTATTATGGG-AAAAGATTG  3467328

Query  610      CCCGTGGTTTATCGGCAGGTCGTGTACAGTCTGTAGCGGTAAAGCTTGTTGTTTGAACGT  669
                ||||||||||||||||||||||||||||||||||||||||||||||||||||| ||||||
Sbjct  3467327  CCCGTGGTTTATCGGCAGGTCGTGTACAGTCTGTAGCGGTAAAGCTTGTTGTT-GAACGT  3467269

Query  670      GAACGTGAAATTCGTGCTTTTATTCCAGAAGAATATTGGCTAGTCTTTGCACACACTAAA  729
                |||||||||||||||||||||||||||||||||||||||| |||||||||| ||||||||
Sbjct  3467268  GAACGTGAAATTCGTGCTTTTATTCCAGAAGAATATTGGCAAGTCTTTGCAGACACTAAA  3467209

Query  730      GCTaaaaaaaaTTGACTTTCGTCTTTGAGGCTGTTAAACAGGCCGGGTAAA-CGTTTAAA  788
                ||||||||| || ||| |||||| ||||||||||||||||||| ||||||| || |||||
Sbjct  3467208  GCTAAAAAAGAT-GACATTCGTC-TTGAGGCTGTTAAACAGGC-GGGTAAAACGCTTAAA  3467152

Query  789      TTaaaaaaaTCAAGCTGAAACCGATCCCCCTGTTTAAATGTTATTAAA-GGCGCTTAAAT  847
                || ||||||| |||||||||| ||| ||| |||| | |||| |||||| |||||| || |
Sbjct  3467151  TT-AAAAAATAAAGCTGAAACAGATGCCC-TGTT-AGATGT-ATTAAAAGGCGCTGAA-T  3467097
```

AbTopoIA Sequence Alignment-2

```
Query  15       AGGCTCCTTTTTGGGTTTGTTTACCTTCTGGTGTTTCAGAACCCACATATTGTGATTGCT  74
                |||||||  ||| ||||||||||||||||||||||||||||||||||||||||||||| |
Sbjct  3465347  AGGCTCC-ATTT-GGTTTGTTTACCTTCTGGTGTTTCAGAACCCACATATTGTGATTGGT  3465404
Query  75       TCTTACGACTAAACTTCACAATGGTTGGGTTACCTTCAGGGTCTACATCTGGAGCTTGTA  134
                ||||||||||||||||||||||||||||||||||||||||||||||||||||||||||||
Sbjct  3465405  TCTTACGACTAAACTTCACAATGGTTGGGTTACCTTCAGGGTCTACATCTGGAGCTTGTA  3465464
Query  135      AAATAAACTGGTATTTTGGATCAAGTTGATCAGCTACGCTACGAAGCTCAGCAACTTTTG  194
                ||||||||||||||||||||||||||||||||||||||||||||||||||||||||||||
Sbjct  3465465  AAATAAACTGGTATTTTGGATCAAGTTGATCAGCTACGCTACGAAGCTCAGCAACTTTTG  3465524
Query  195      GTGCACGTGTTTCACGAATTTTCGGGAACTTACTTGCAGCCAAGAACAGACCAGCCGCGC  254
                ||||||||||||||||||||||||||||||||||||||||||||||||||||||||||||
Sbjct  3465525  GTGCACGTGTTTCACGAATTTTCGGGAACTTACTTGCAGCCAAGAACAGACCAGCCGCGC  3465584
Query  255      CATCACGTAACACAAAATAATCATCATGCTTGGTTGAACGCAAGTGTTCCATCTTGATTG  314
                ||||||||||||||||||||||||||||||||||||||||||||||||||||||||||||
Sbjct  3465585  CATCACGTAACACAAAATAATCATCATGCTTGGTTGAACGCAAGTGTTCCATCTTGATTG  3465644
Query  315      GTTCTACACGTGGTGGCGCAGGTTGACCATTTTTCAAGACTTTACGAGTATTGTCACAGC  374
                ||||||||||||||||||||||||||||||||||||||||||||||||||||||||||||
Sbjct  3465645  GTTCTACACGTGGTGGCGCAGGTTGACCATTTTTCAAGACTTTACGAGTATTGTCACAGC  3465704
Query  375      TTGTACAAGCGAAATATGGGCCGAAGCGACCAATCTTAAGCTGCATTTCACCATCACATT  434
                |||||||||||||||||||||||||||||||| |||||||||||||||||||||||||||
Sbjct  3465705  TTGTACAAGCGAAATATGGGCCGAAGCGACCAGTCTTAAGCTGCATTTCACCATCACATT  3465764
Query  435      TATCACATGGAATGGTTGGGCCATCATAACCTTTGATCTTGAACTCGCCTTCTTCAAGCT  494
                ||||||||||||||||||||||||||||||||||||||||||||||||||||||||||||
Sbjct  3465765  TATCACATGGAATGGTTGGGCCATCATAACCTTTGATCTTGAACTCGCCTTCTTCAAGCT  3465824
Query  495      CATAGCCATCGCAATCCGGGTTGTTACCACAAACATGTAACTTACGGCCACCATCAATGA  554
                ||||||||||||||||||||||||||||||||||||||||||||||||||||||||||||
Sbjct  3465825  CATAGCCATCGCAATCCGGGTTGTTACCACAAACATGTAACTTACGGCCACCATCAATGA  3465884
Query  555      CGTAGCTGTCCATTGCTGTACCACATTTCGGACAACGATGTTTTGACATCAAGTCTGCTG  614
                ||||||||||||||||||||||||||||||||||||||||||||||||||||||||||||
Sbjct  3465885  CGTAGCTGTCCATTGCTGTACCACATTTCGGACAACGATGTTTTGACATCAAGTCTGCTG  3465944
Query  615      TTTCAGCACTATCATCATCAGATAACGCTGCTAGAGACTCAACTGGCGTTAAGTTCAAGG  674
                ||||||||||||||||||||||||||||||||||||||||||||||||||||||||||||
Sbjct  3465945  TTTCAGCACTATCATCATCAGATAACGCTGCTAGAGACTCAACTGGCGTTAAGTTCAAGG  3466004
Query  675      TCCCTTTACAGCGTTCCTTAGGCGGCAAGTTATAGCCAGAACACCCTAAGAATACCCCTG  734
                ||||||||||||||||||||||||||||||||||||||||||||||||||||||| |||
Sbjct  3466005  TCCCTTTACAGCGTTCCTTAGGCGGCAAGTTATAGCCAGAACACCCTAAGAATACGCCT-  3466063
Query  735      GTAGTGCCCGTACGAAATCTGCAATTGGACGTGAACATTCAGGGCAATGTACTGCTGGTA  794
                |||||||||||||||| ||||| |||||||||||||||||||||||||||||||||||||
Sbjct  3466064  GTAGTGCCCGTACGAA-TCTGC-ATTGGACGTGAACATTCAGGGCAATGTACTGCTGGTA  3466121
Query  795      CTTCAACTGGCTGGTTACGACGCATTCCCTGCTCACCTTGAGCATGGGTCAAACGCTTTT  854
                ||||||||||||||||||||||||||||||||||||||||||||| ||||||||||||||
Sbjct  3466122  CTTCAACTGGCTGGTTACGACGCATTCCCTGCTCACCTTGAGCATTGGTCAAACGCTTTT  3466181
Query  855      AAAAACCACCATAGAATGTGGTCTAGCAATTCCCTCCAATTACGCCTCACCTGGTTGCAA  914
                 |||| ||||||||||||| ||||||||||||| |||| |||||| ||||||| ||||||
Sbjct  3466182  TAAAATCACCATAGAATGT-GTCTAGCAATTCCTTCCAGTTACGC-TCACCTG-TTGCAA  3466238
Query  915      CTTTTATCCGGAGCTGAGACTTTCAAGGGTCTGGCTGTAAATGGCATAGTCCATCAGGGT  974
                |||| ||| | ||||||  ||| ||||| |||| |||||||| ||||||| ||||||| |
Sbjct  3466239  CTTT-ATC-G-AGCTGAC-CTT-CAAGG-TCTG-CTGTAAAT-GCATAGTTCATCAGG-T  3466289
Query  975      TCATTAAAACTTTTCATCAGAGAGACGATCCCGGCTTACGATTCTCCCCCCATTTTC  1031
                | ||||||||||| ||||| |||  |||||| |  ||||||| ||| ||| ||||||
Sbjct  3466290  T-ATTAAAACTTT-CATCA-AGA--CGATCC-G--TTACGAT-CTCACCC-ATTTTC  3466336
```

EcTopoIII Sequence Alignment-3

```
Query  37   CGCGCCATTGCTGATGTCCTGCCCAAACCGCACCGGAAAGGCGATGGCTTTATCGAGTGC  96
Sbjct  2    CGCGCCATTGCTGATGTCCTGCCCAAACCGCACCGTAAAGGCGATGGCTTTATCGAGTGC  61
Query  97   GGTAATGGTCAGGTGGTGACCTGGTGTATCGGTCACCTGCTTGAGCAGGCGCAGCCAGAC  156
Sbjct  62   GGTAATGGTCAGGTGGTGACCTGGTGTATCGGTCACCTGCTTGAGCAGGCGCAGCCAGAC  121
Query  157  GCCTACGACAGCCGCTATGCGCGCTGGAATCTTGCGGATTTGCCGATTGTCCCGGAAAAG  216
Sbjct  122  GCCTACGACAGCCGCTATGCGCGCTGGAATCTTGCGGATTTGCCGATTGTCCCGGAAAAG  181
Query  217  TGGCAATTACAGCCCCGACCCTCCGTGACCAAACAACTTAACGTCATCAAACGGTTCCTG  276
Sbjct  182  TGGCAATTACAGCCCCGACCCTCCGTGACCAAACAACTTAACGTCATCAAACGCTTCCTG  241
Query  277  CATGAAGCCAGCGAAATCGTTCACGCCGGGGACCCGGATCGTGAAGGGCAATTGCTGGTG  336
Sbjct  242  CATGAAGCCAGCGAAATCGTTCACGCCGGGGACCCGGATCGTGAAGGGCAATTGCTGGTG  301
Query  337  GATGAAGTGCTGGACTATCTGCAACTGGCACCGGAAAAGCGCCAGCAGGTACAGCGTTGC  396
Sbjct  302  GATGAAGTGCTGGACTATCTGCAACTGGCACCGGAAAAGCGCCAGCAGGTGCAGCGTTGC  361
Query  397  TTGATAAACGACCTGAACCCGCAGGCGGTTGAGCGGGCGATCGACCGTCTTCGTTCCAAC  456
Sbjct  362  TTGATAAACGACCTGAACCCGCAGGCGGTTGAGCGGGCGATCGACCGTCTTCGTTCCAAC  421
Query  457  AGTGAGTTTGTACCGCTGTGCGTTTCTGCGCTGGCGCGAGCGCGTGCCGACTGGCTGTAC  516
Sbjct  422  AGTGAGTTTGTGCCATTGTGCGTTTCTGCGCTGGCGCGAGCGCGTGCCGACTGGCTGTAC  481
Query  517  GGCATCAATATGACCCGTGCGTATACCATTCTCGGTCGCAATGCCGGTTATCAGGGCGTA  576
Sbjct  482  GGCATCAATATGACCCGTGCGTATACCATTCTCGGTCGCAATGCCGGTTATCAGGGCGTA  541
Query  577  CTTTCCGTGGGACGCGTGCAGACGCCCGTGCTTGGGCTGGTGGTGCGCCGCGATGAAGAG  636
Sbjct  542  CTTTCCGTGGGACGCGTGCAGACACCCGTGCTCGGGCTGGTGGTGTGCCGCGATGAAGAG  601
Query  637  ATTGAAAACTTCGTGGCGAAAGACTTCTTTGAAGTCAAAGCACATATCGTGACACCTGCC  696
Sbjct  602  ATTGAAAACTTCGTGGCGAAAGACTTCTTTGAAGTCAAAGCGCATATCGTGACACCTGCC  661
Query  697  GATGAGCGGTTTACCGCTATCTGGCAACCGAGCGAAGCGTGTGAACCGTACCAGGATGAA  756
Sbjct  662  GATGAGCGGTTTACCGCTATCTGGCAACCGAGCGAAGCGTGTGAACCGTACCAGGATGAA  721
Query  757  GAAGGGCGCTTGTTACATCGTCCACTGGCGGAGCATGTGGTTAACCGCATTAGTGGTCAA  816
Sbjct  722  GAAGGGCGCTTGTTACATCGTCCACTGGCGGAGCATGTGGTTAACCGCATTAGTGGTCAA  781
Query  817  CCGGCTATTGTCACCAGCTATAACGATAAACGGGAATCAGAATCCGCGCCGCTGCCTTTT  876
Sbjct  782  CCGGCTATTGTCACCAGCTATAACGATAAACGGGAATCAGAATCCGCGCCGCTGCCGTTT  841
Query  877  TCGCTTTCAGCGTTGCAGATTGAAGCGGCAAAACGTTTTGGTCTGAGTGCGCAGAACGTG  936
Sbjct  842  TCGCTTTCGGCGTTGCAGATTGAAGCGGC-AAACGCTTTGGTCTGAGCGCGCAG-ACGTG  899
Query  937  CTTGATATCTGCCAGAAACTGTACGAAACGCACAAGCTAATCACTTATC  985
Sbjct  900  CTTGATATCTGCCAGAG-CTGTACG-AACACACAAGTTAATCACTTATC  946
```

EcTopoIII Sequence Alignment-4

```
Query  995   ATTGTCGCTATTTGCCAGAAGAACATTTTGCCGGACGCCACGCGGTGATGAATGCCATCA  1054
Sbjct  937   ATTGTCGCTATTTGCCAGAAGAACATTTTGCCGGACGCCACGCGGTGATGAATGCCATCA  878
Query  1055  GTGTTCATGCACCGGATCTGTTGCCGCAGCCAGTGGTAGATCCAGATATACGCAACCGCT  1114
Sbjct  877   GCGTTCATGCACCAGATCTGTTGCCGCAGCCAGTGGTAGATCCAGATATACGCAACCGCT  818
Query  1115  GTTGGGATGACAAAAAGGTCGATGCGCACCACGCCATCATTCCGACCGCACGGAGTTCTG  1174
Sbjct  817   GTTGGGATGACAAAAAGGTCGATGCGCACCACGCCATCATTCCGACCGCACGGAGTTCTG  758
Query  1175  CGATCAACCTGACGGAGAACGAAGCGAAGGTCTATAACCTGATTGCCCGTCAGTATCTGA  1234
Sbjct  757   CGATCAAC-TGACGGAGAACGAAGCGAAGGTCTATAACCTGATTGCCCGTCAGTATCTGA  699
Query  1235  TGCAATTCTGCCCGGATGCGGTGTTCCGCAAGTGTGTTATCGAACTGGACATTGCCAAAG  1294
Sbjct  698   TGCAGTTCTGCCCGGATGCGGTGTTCCGCAAGTGTGTTATCGAACTGGACATTGCCAAAG  639
Query  1295  GCAAATTTGTCGCTAAAGCGCGTTTTCTTGCTGAAGCAGGCTGGCGCACGCTGTTAGGCA  1354
Sbjct  638   GCAAATTTGTCGCTAAAGCGCGTTTTCTTGCTGAAGCAGGTTGGCGCGCGCTGTTAGGCA  579
Query  1355  GCAAAGAGCGCGATGAAGAAAACGACGGCACGCCACTGCCTGTGGTGGCGAAAGGCGATG  1414
Sbjct  578   GCAAAGAGCGCGATGAAGAAAACGACGGCACGCCATTGCCTGTGGTGGCGAAAGGCGATG  519
Query  1415  AGTTGCTGTGTGAAAAAGGTGAAGTGGTAGAGCGGCAAACCCAGCCGCCGCGCCATTTTA  1474
Sbjct  518   AGTTACTGTGTGAAAAAGGTGAAGTGGTAGAGCGGCAAACCCAGCCGCCGCGCCATTTTA  459
Query  1475  CCGATGCAACACTGCTTTCGGCGATGACCGGGATCGCGCGCTTTGTGCAGGATAAAGATC  1534
Sbjct  458   CCGATGCAACACTGCTTTCGGCGATGACCGGGATTGCGCGCTTTGTGCAGGACAAAGATC  399
Query  1535  TGAAAAAGATCCTCCGTGCGACCGATGGTCTGGGGACAGAGGCAACGCGTGCCGGGATTA  1594
Sbjct  398   TGAAAAAGATCCTTCGTGCGACCGATGGTCTGGGGACAGAAGCAACGCGTGCCGGGATTA  339
Query  1595  TTGAACTGTTGTTCAAGCGTGGTTTCCTGACCAAAAAAGGGCGCTATATCCACTCCACCG  1654
Sbjct  338   TTGAACTGTTGTTCAAGCGTGGTTTCCTCACCAAAAAAGGGCGCTATATCCACTCCACCG  279
Query  1655  ACGCCGGAAAAGCGCTATTCCATTCGCTGCCGGAGATGGCGACGCGACCGGACATGACCG  1714
Sbjct  278   ACGCCGGAAAAGCGTTATTCCATTCGCTGCCAGAAATGGCTACGCGACCGGACATGACCG  219
Query  1715  CGCACTGGGAATCGGTGCTGACGCAAATCAGCGAAAAGCAGTGTCGCTATCAGGACTTTA  1774
Sbjct  218   CGCACTGGGAATCGGTGCTGACGCAAATCAGCGAAAAGCAGTGTCGCTATCAGGACTTTA  159
Query  1775  TGCAGCCGCTGGTGGGGACGCTATATCAGCTTATTGATCAAGCCAAACGTACGCCGGTGC  1834
Sbjct  158   TGCAGCCGCTGGTGGGGACGCTATATCAGCTTATTGATCAAGCCAAACGTACTCCGGTGC  99
Query  1835  GGCAGTTTCGCGGCATTGTGGCTCCGGGCAGTGGTGGCAGTGCTGATAAGAAAAAGGCTG  1894
Sbjct  98    GGCAGTTTCGCGGCATTGTGGCTCCGGGCAGTGGTGGCAGTGCTGATAAGAAAAAGGCTG  39
Query  1895  CACCGCGTAAACGTAGTGCGaaaaaaaGTCCGCCAGC  1931
Sbjct  38    CACCGCGTAAACGTAGTGCGAAAAAAAGTCCGCCAGC  2
```

Supplementary Figure 9. Cloning of topoisomerase I and III. (a) Cloning of *S. aureus* topoisomerase IA. L-1kb DNA ladder, DD- Double digested (NdeI/BamHI) Vector pET28a(+)of positive clone of SaTopo IA, SD–Single digested (NdeI). (b) Cloning of *A. baumannii* topoisomerase IA. L-1kb DNA ladder, SD–Single digested (XhoI), DD- Double digested (XhoI/BamHI) digested vector pET28a(+)of positive clone of SaTopoIA. (c) Cloning of *E. coli* topoisomerase III. L-1kb DNA ladder, DD- Double digested (XhoI/BamHI) digested Vector pET28a(+) of positive clone of EcTopoIII, UC –Uncut plasmid of positive clone. (d) Sequence alignment of cloned of *S. aureus* topoisomerase I (SaTopo IA), *A. baumannii* topoisomerase IA (AbTopo IA) and*E.coli* topoisomerase III (EcTopo III).

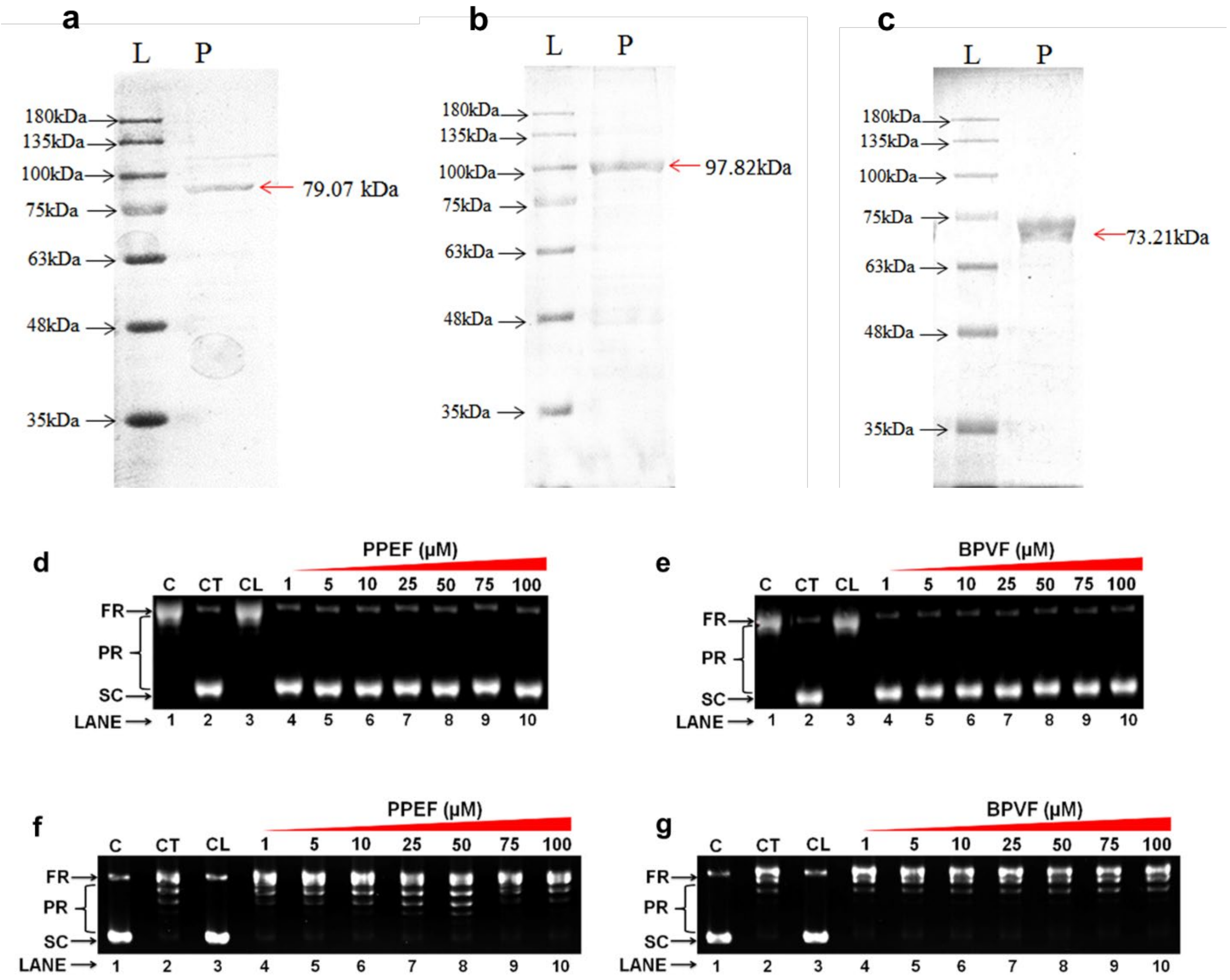

Supplementary Figure 10. Expression, relaxation inhibition assay of topoisomerase IA and gyrase inhibition. (a) Gel of purified SaTopoIA (79.07kDa), L-Ladder, P- Purified protein. (b) Gel of purified AbTopoIA (97.82kDa), L-Ladder, P- Purified. (c) Gel of purified EcTopoIII (73.21kDa), L-Ladder, P- Purified. (d, e) Analysis of supercoiling of relaxed pHOT I plasmid with SaGyrase in the presence of the compound, lane 1-pHOT I relaxed plasmid DNA (C); lane 2- supercoiling of relaxed pHOT1 plasmid DNA by SaGyrase (CG); lane 3-relaxed pHOT I plasmid DNA in the presence of compound (CL); lanes 4−10- supercoiling inhibition of relaxed pHOT I plasmid DNA by SaGyrase in the presence of 1, 5, 10, 25, 50, 75, and 100 μM of compound respectively. All panels show representative results for an experiment repeated at least three times. (f) Inhibition of relaxation Activity of HuTopoI IA: lane 1- pHOT1 plasmid DNA (C); lane 2- relaxation of plasmid DNA by topo I (CT); lanes 3−7- inhibition of relaxation of plasmid DNA by topo I in the presence of 1, 5, 10, 25, 50, 75 and 100μM of BPVF respectively (Data show representative results for an experiment repeated at least three times). (g) Inhibition of relaxation Activity of HuTop I: lane 1- pHOT1 plasmid DNA (C); lane 2- relaxation of plasmid DNA by topoI (CT); lanes 3−7- inhibition of relaxation of plasmid DNA by topo I in the presence of 1, 5, 10, 25, 50, 75 and 100 μM of BPVF. (Data show representative results for an

experiment repeated at least three times). (Data show representative results for an experiment repeated at least three times).

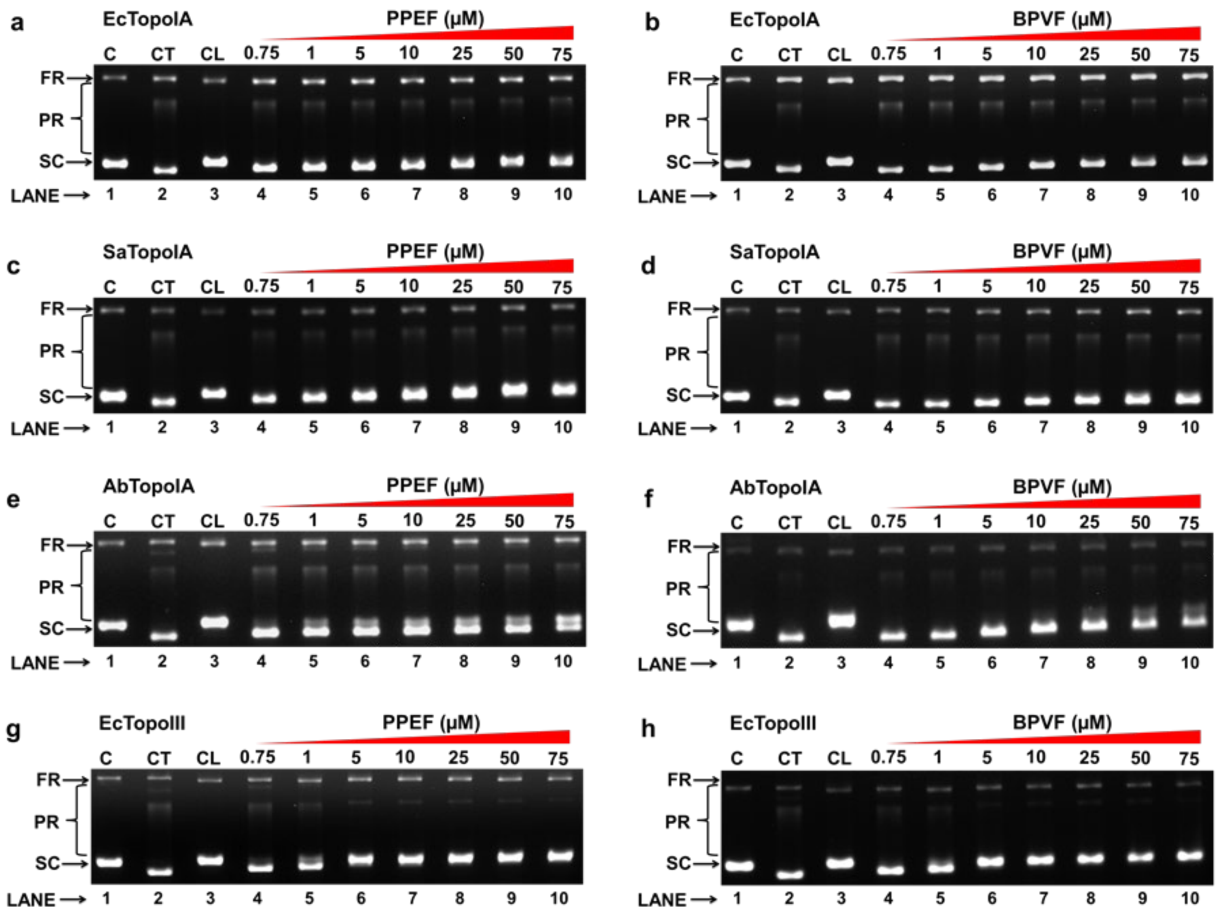

Supplementary Figure 11. Relaxation inhibition activity of PPEF and BPVF against ECTopoIA, SaTopoIA, AbTopoIA, and EcTopoIII enzyme in EtBr containing agarose gel. (a,b) Inhibition of relaxation activity of EcTopoIA against PPEF and BPVF respectively. Lane 1: pHOT-1 plasmid DNA; Lane 2: Relaxation of plasmid DNA by topoI; Lane 3: pHOT-1 plasmid DNA with 75 μM PPEF; Lanes 4−10: Inhibition of relaxation of plasmid DNA by topoI in the presence of 0.75, 1, 5, 10, 25, 50, and 75 μM PPEF/BPVF. (c,d) Inhibition of relaxation activity of SaTopoIA against PPEF and BPVF respectively. Lane 1: pHOT-1 plasmid DNA; Lane 2: Relaxation of plasmid DNA by topoI; Lane 3: pHOT-1 plasmid DNA with 75 μM PPEF; Lanes 4−10: Inhibition of relaxation of plasmid DNA by topoI in the presence of 0.75, 1, 5, 10, 25, 50, and 75 μM PPEF/BPVF. (e,f) Inhibition of relaxation activity of AbTopoIA against PPEF and BPVF respectively. Lane 1: pHOT-1 plasmid DNA; Lane 2: Relaxation of plasmid DNA by topoI; Lane 3: pHOT-1 plasmid DNA with 75 μM PPEF; Lanes 4−10: Inhibition of relaxation of plasmid DNA by topoI in the presence of 0.75, 1, 5, 10, 25, 50, and 75 μM PPEF/BPVF. (g,h) Inhibition of relaxation activity of

EcTopoIII against PPEF and BPVF respectively. Lane 1: pHOT-1 plasmid DNA; Lane 2: Relaxation of plasmid DNA by topoI; Lane 3: pHOT-1 plasmid DNA with 75 µM PPEF; Lanes 4−10: Inhibition of relaxation of plasmid DNA by topoI in the presence of 0.75, 1, 5, 10, 25, 50, and 75 µM PPEF/BPVF. All panels show representative results for an experiment repeated at least three times. FR-Fully Relaxed DNA; PR-Partially Relaxed DNA; S-Supercoiled DNA.

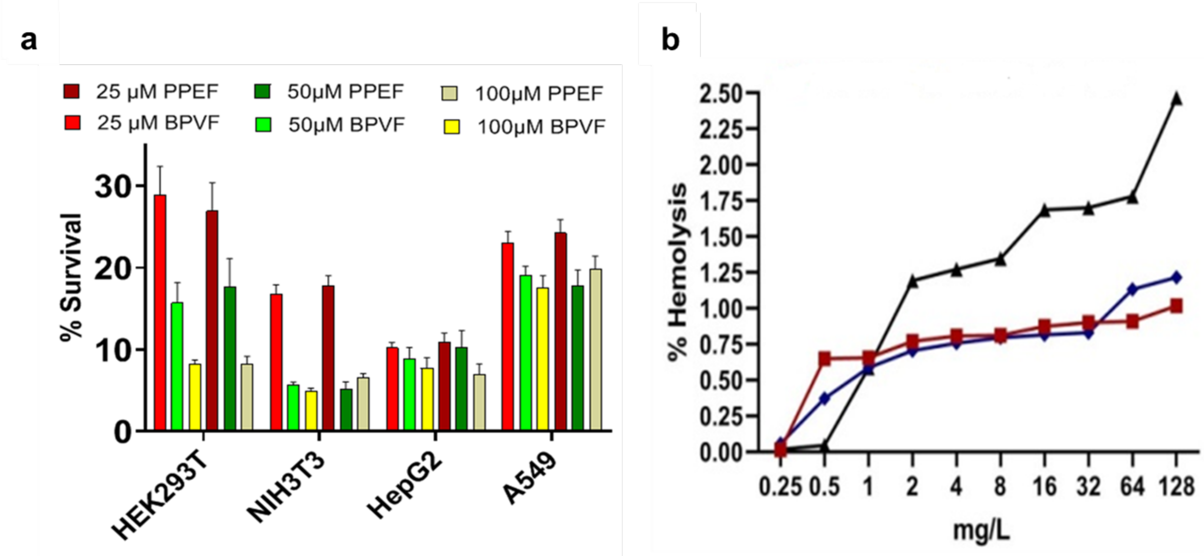

Supplementary Figure 12.Toxicity in human cell line, and haemolytic analysis. (a) The toxicity of ABTI19b and ABTI16b on HEK293T a human embryonic kidney 293 cells line, NIH3T3, a mouse embryonic fibroblast cell line, HepG2 a human liver cancer cell line and A549, a lung carcinoma cell line with different concentration. (b) The toxicity of PPEF (Blue) BPVF (Red) and ciprofloxacin (CIP) (Black) on human erythrocytes cell at concentration range is from 0.5 to 128 mg/L (250 µM).

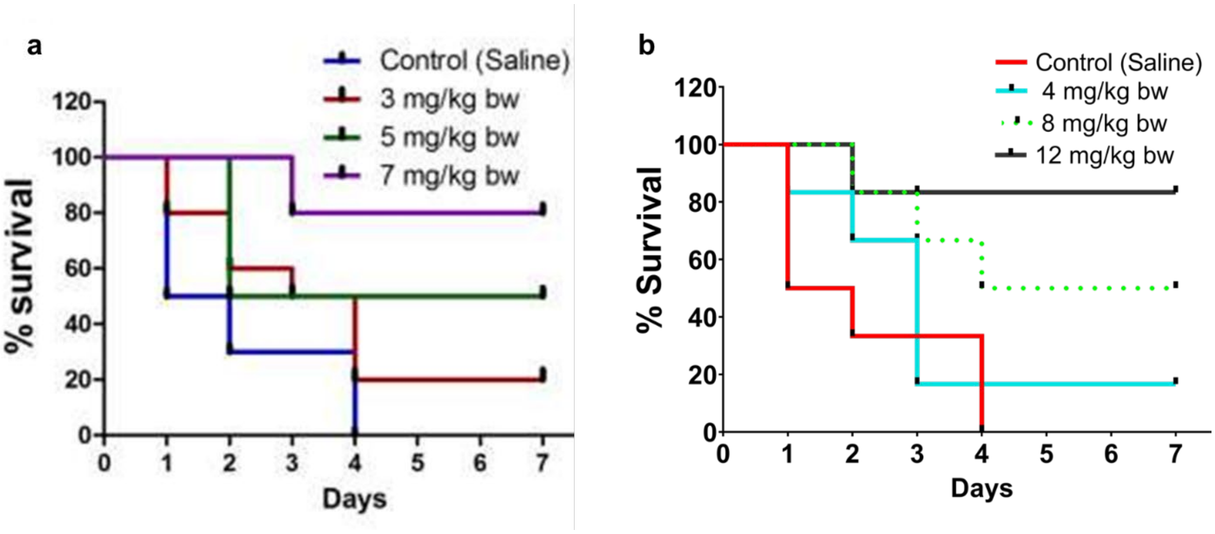

Supplementary Figure 13. Animal efficacy against *E.coli* K12 (ATCC25922) in murine sepsis and thigh infection models. (a) Efficacy of compound PPEF in mouse systemic infection model. Graphical representation of percentage survival versus drug dose (mg/kg body weight). (b) Efficacy of compound BPVF in mouse systemic infection model. Graphical representation of percentage survival versus drug dose (mg/kg body weight).

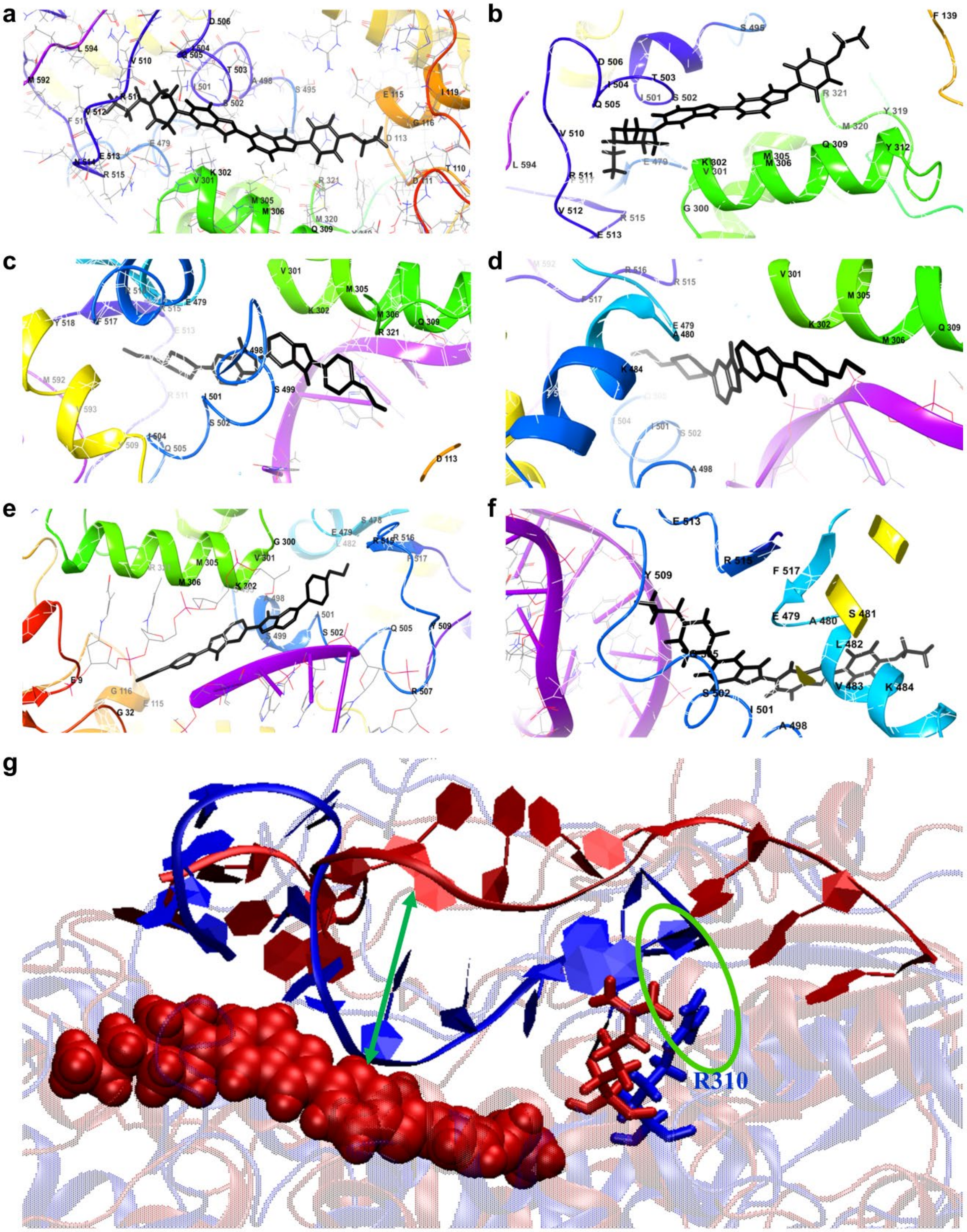

Supplementary Figure 14. 2-D interaction diagrams for PPEF, dsDNA/ssDNA and TopoIA. (a) and (b) Represent the closed state and partially close state of TopoIA in presence of PPEF. (c) and (d)Represent the closed state and partially close state of TopoIA in presence of PPEF and dsDNA. (e) and (f) Represent

the closed and partially close state of TopoIA in presence of PPEF and ssDNA. PPEF (Black) and DNA (Purple). (g) Conformation of ssDNA in presence (blue color) and absence (red color) of PPEF. The protein is shown in ribbon representation with partially transparent state. The backbone of ssDNA is shown as ribbon and the bases are shown as fill ring. The figure shows that the PPEF (red color, vdW representation) push the ssDNA away (green color arrow) from the protein cavity. Additionally, PPEF break the strongest interaction between ssDNA and amino acid R310 (highlighted in green color circle).

Supplementary Figure 15. Full length unprocessed cleavage and relaxation assay gels.

Full length uncropped gel of cleavage figure 2e (PPEF)

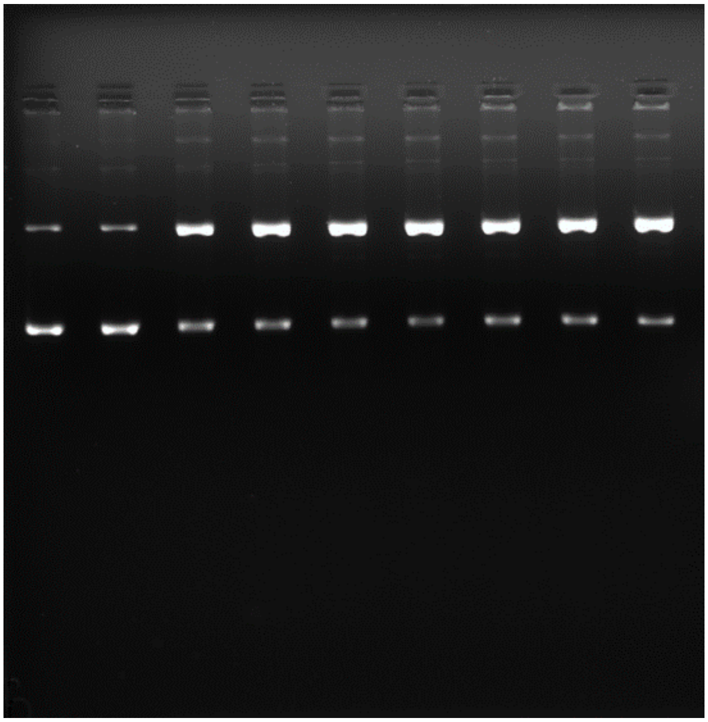

Full length uncropped gel of cleavage figure 2e (BPVF)

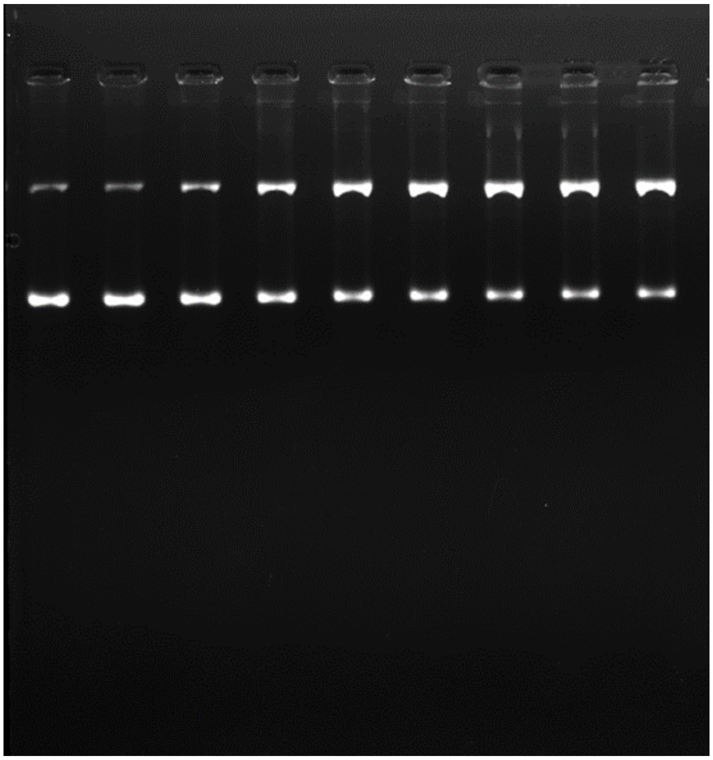

Full length uncropped gel of relaxation figure 3a (*E. coli* TopoIA, PPEF)

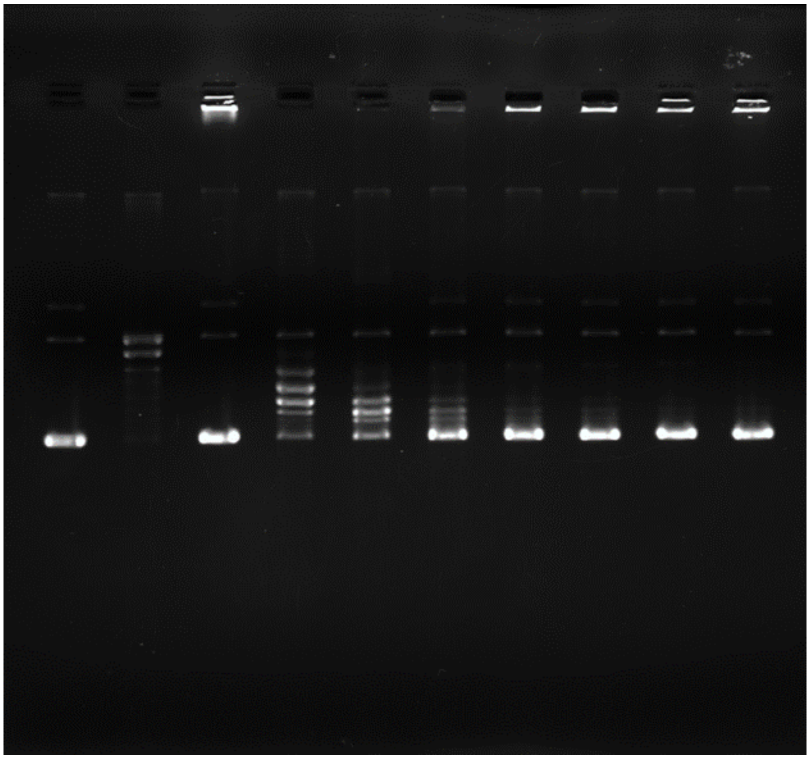

Full length uncropped gel of relaxation figure 3a (*S. aureus* TopoIA, PPEF)

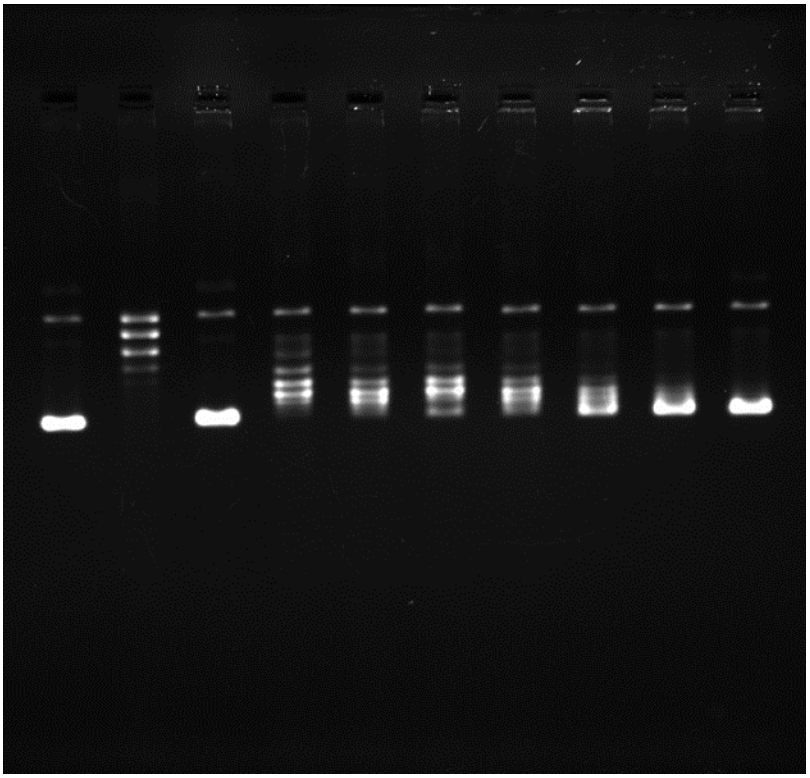

Full length uncropped gel of relaxation figure 3a (*A. baumannii* TopoIA, PPEF)

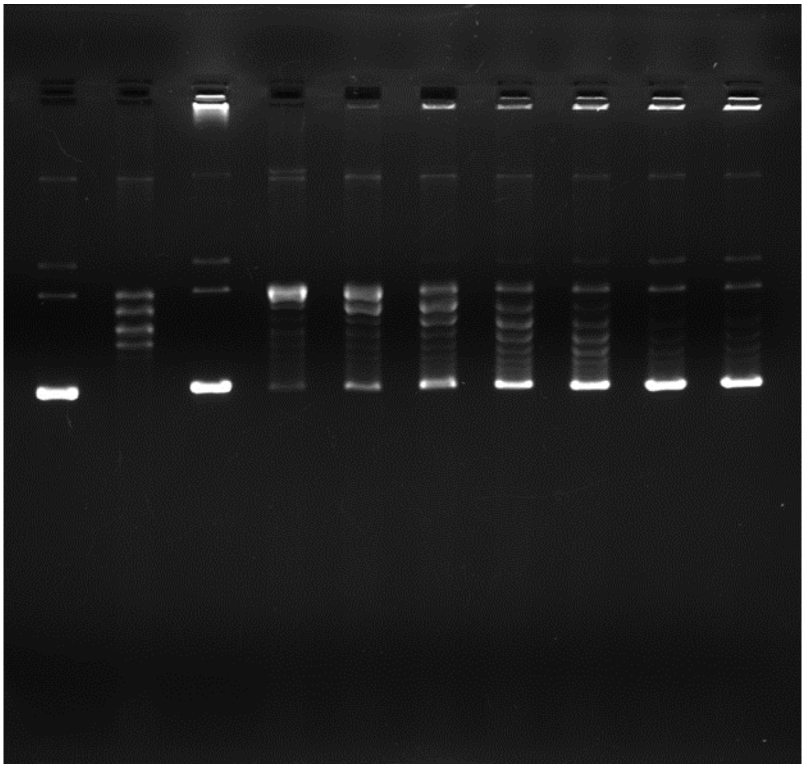

Full length uncropped gel of relaxation figure 3c (*E. coli* TopoIA, BPVF)

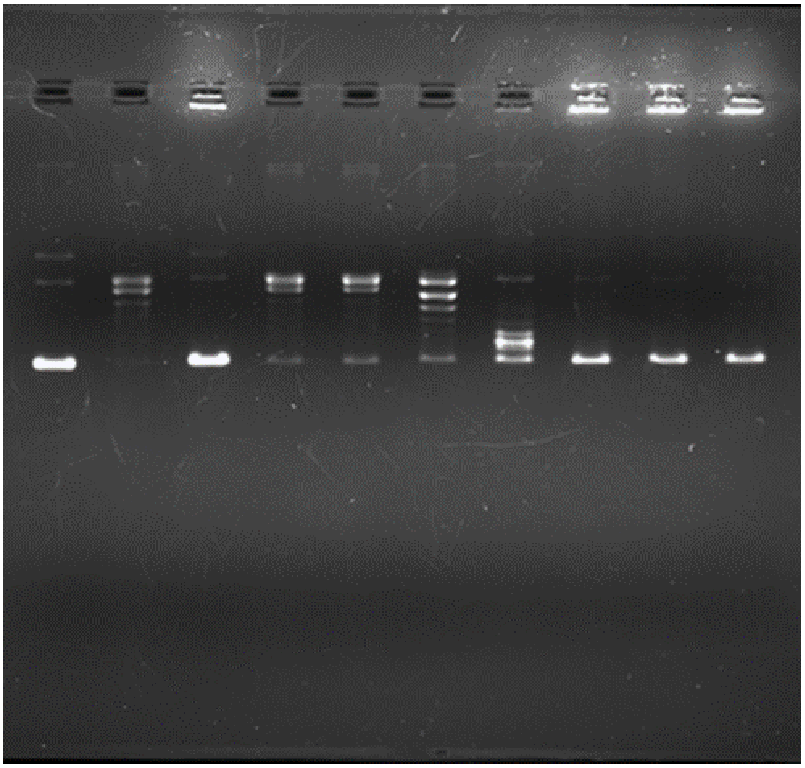

Full length uncropped gel of relaxation figure 3b (*S. aureus* TopoIA, BPVF)

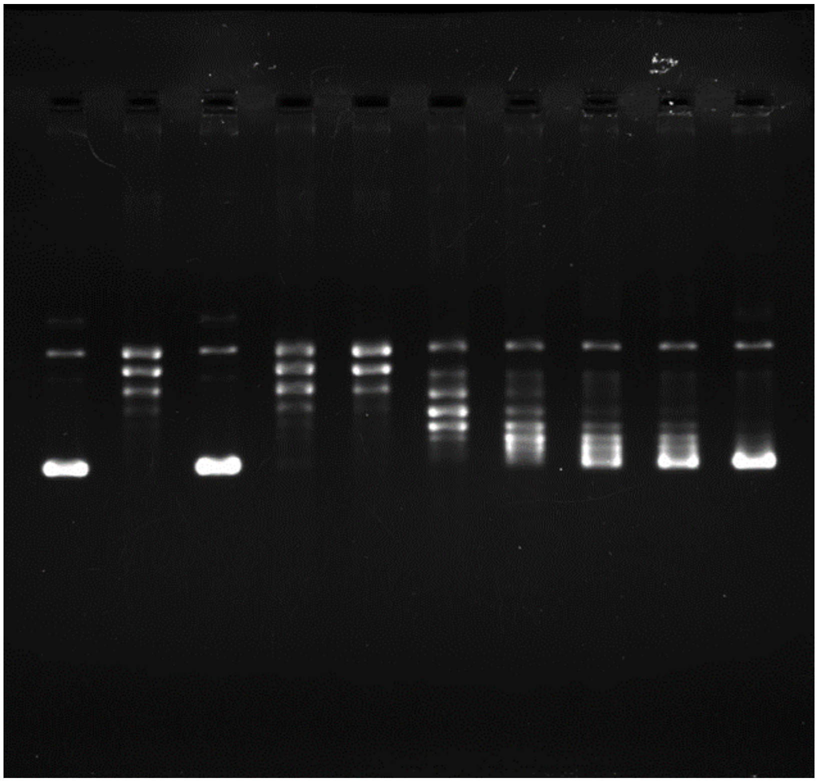

Full length uncropped gel of relaxation figure 3b (*A. baumannii* TopoIA, BPVF)

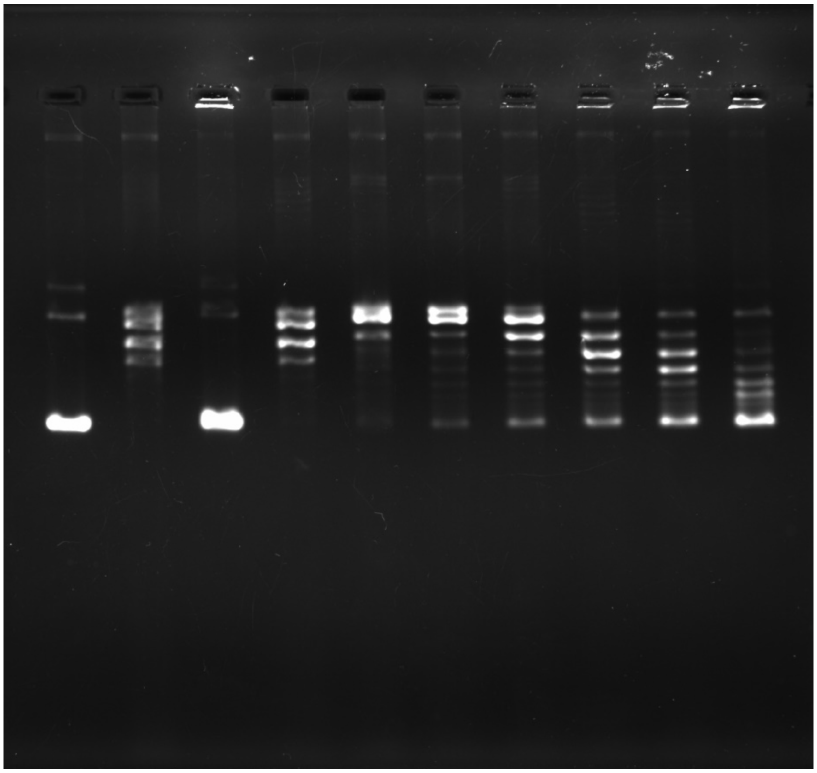

Full length uncropped gel of relaxation figure 3c (*E. coli* TopoIII, PPEF)

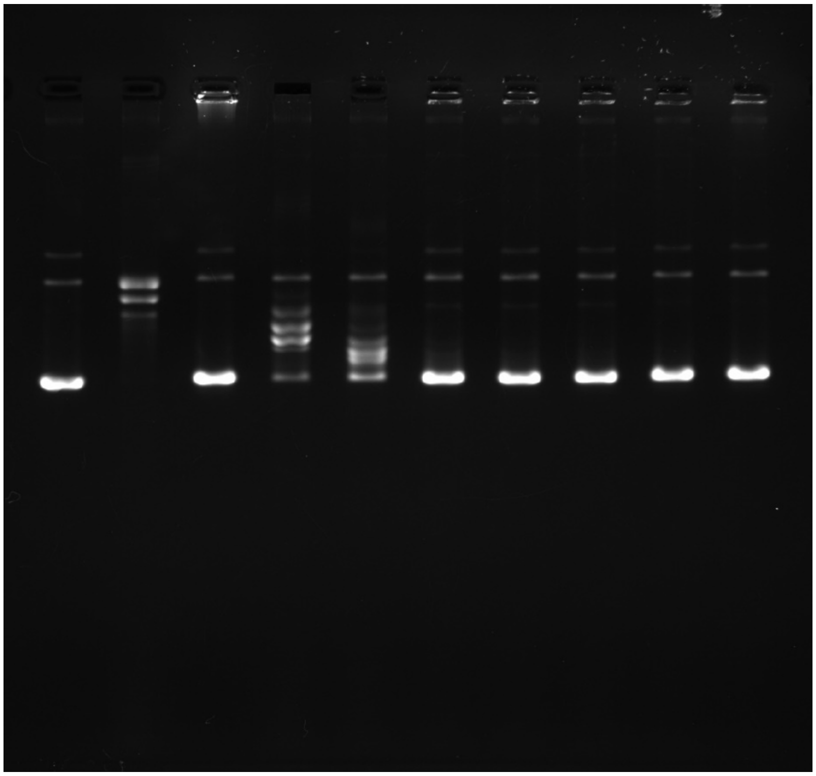

Full length uncropped gel of relaxation figure 3c (*E. coli* TopoIII, BPVF)

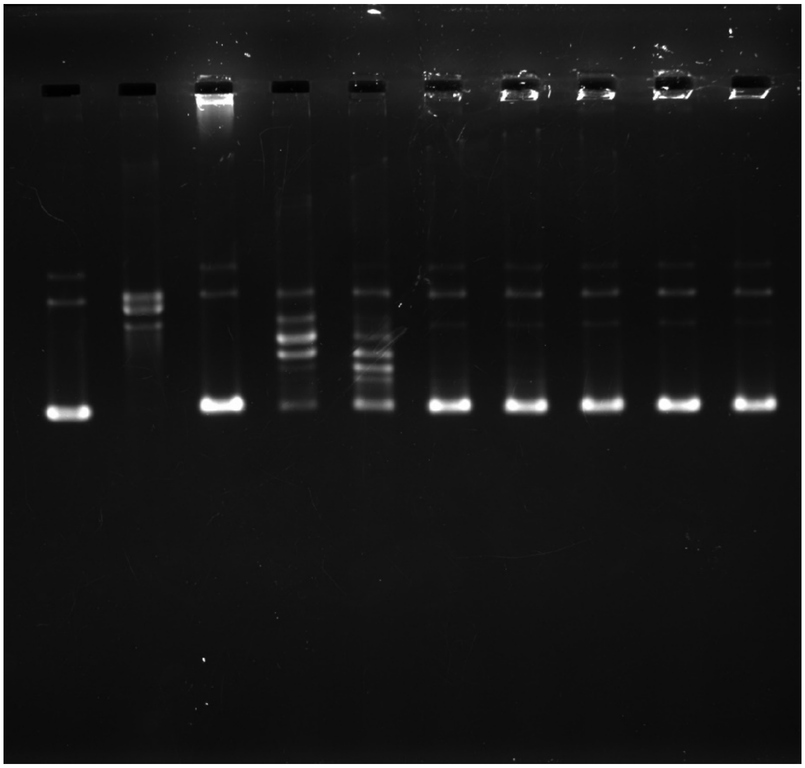

Full length uncropped gel of cleavage supplementary figure 2e (PPEF)

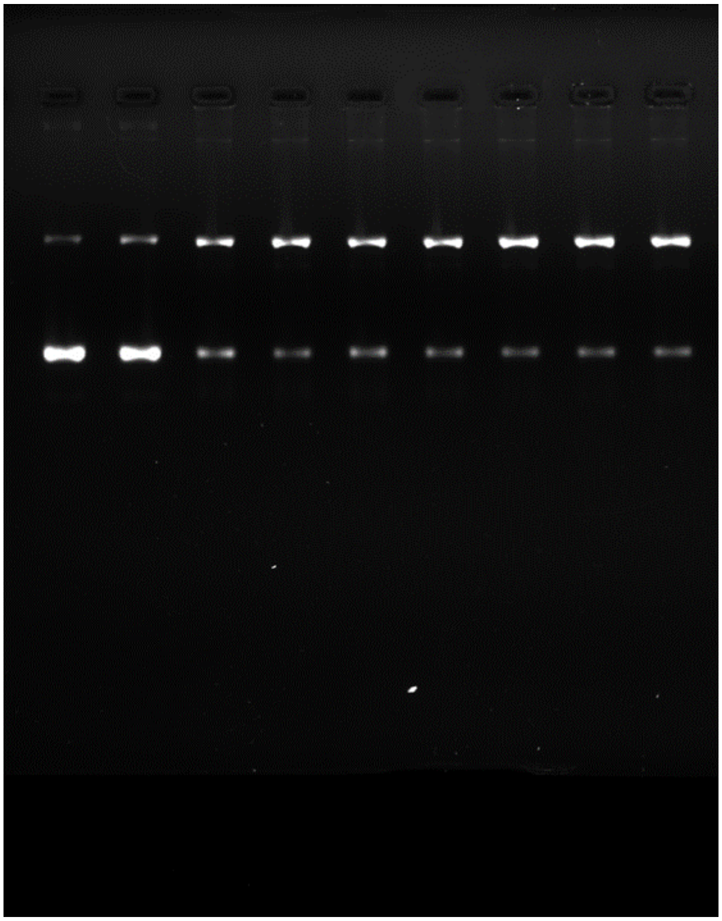

Supplementary Tables

Supplementary Table 1. System setup of MD simulations.

System	Box size (nm x nm x nm)	Total of atoms (system)	Number of water molecules	Number of ions (Mg^{2+} and Cl^{-})
TopoIA	12x12x12	9455	52217	143
TopoIA-PPEF	12x12x12	9523	52000	142
TopoIA-dsDNA	12x12x12	10278	52948	321
TopoIA-ssDNA	12x12x12	9871	53060	315
TopoIA-PPEF-dsDNA	12x12x12	10346	52908	321
TopoIA-PPEF-ssDNA	12x12x12	9939	53001	315

Supplementary Table 2. Susceptibility of BPVF and standard antibiotics against the Water-Borne *E. coli* and Urinary tract infection (UTI) causing strains.

Compound Name		DND14*	IP11*	IST*	PA*	KK45*	NG37*	MKND*	PA24*	KK5S*	IS40*	KK38*	KP31*	WB23*
**BPVF (µg/ml)**	**MIC**	1	2	2	2	2	2	4	4	1	2	4	1	4
	**MBC**	1	2	2	2	2	2	4	4	1	2	4	1	4
**AMP (µg/ml)**	**MIC**	16	16	32	16	>128	4	>128	32	4	32	>128	>128	>128
	**MBC**	16	16	32	16	ND	4	ND	32	4	32	ND	ND	ND
**KAN (µg/ml)**	**MIC**	4	4	8	2	>128	8	32	2	4	8	4	4	4
	**MBC**	4	4	8	2	ND	8	32	2	4	8	4	4	4
**TRIM (µg/ml)**	**MIC**	0.25	1	>128	0.25	>128	>128	>128	0.5	>128	>128	>128	>128	>128
	**MBC**	0.25	1	>128	0.25	ND	ND	ND	0.5	ND	ND	ND	ND	ND
**CHL (µg/ml)**	**MIC**	2	2	4	2	>128	4	>128	>128	4	2	2	1	2
	**MBC**	2	2	4	2	ND	4	ND	ND	4	2	2	1	2
**STR (µg/ml)**	**MIC**	4	4	8	8	>128	8	8	8	4	ND	ND	4	32
	**MBC**	4	4	8	8	ND	8	8	8	4	ND	ND	4	32
**TET (µg/ml)**	**MIC**	4	8	32	8	>128	8	>128	8	8	ND	ND	ND	ND
	**MBC**	4	8	32	8	ND	8	ND	8	8	ND	ND	ND	ND
**GEN (µg/ml)**	**MIC**	2	2	4	1	>128	2	8	1	1	2	2	1	1
	**MBC**	2	2	4	1	ND	2	8	1	1	2	2	1	1

Supplementary Table 3. Susceptibility of BPVF against the standard and mutant *E.coli* strains.

S. No	Strain details	Name	MIC (µg/ml)	MBC (µg/ml)
1.	*E. coli* (CGSC5073)	K12	16	16
2.	CGSC8229	*ΔtopA*	4	4
3.	CGSC9474	*ΔtopB*	16	16
4.	CGSC11843	*ΔacrA*	8	8
5.	CGSC 10098	*ΔemrA*	16	16
6.	CGSC11430	*ΔtolC*	1	1
7.	*E. coli* MG1655	MG1655	16	16
8.	MG1655 Δ*yciM*::Kan/pMN103	*ΔyciM*	4	4
9.	MG1655 *lpxC1272 leuB*::Tn*10*	*ΔlpxC*	1	1
10.	MG1655 *lpxD14* (*skp*::Tn*10*dTet	*ΔlpxD*	1	1

Supplementary Table 4. Susceptibility of PPEF, BPVF and ciprofloxacin to different *E. coli* gyrase mutants clinical isolates mutation.

E.coli Isolates	Ciprofloxacin MIC	PPEF MIC	BPVF MIC	*gyrA*	*parC*	*parE*
EC118	>16	3.3	32	S83L	---	---
EC49HSV	>64	2.1	32	S83L,D87N	---	S458A
EC555	>64	4.4	32	S83L, D87N	S80I, E84V	S485A
EC284LF	>64	4.1	64	S83L, D87N	S80I	E460D

Supplementary Table 5. ED_{50},LD_{50}, and TI of PPEF and BPVF at oral and IV doses in BALB/c mice.

Compound name	Route of Administration	Tested Conc. (mg/kg.bw)	LD_{50} (mg/kg.bw)	ED_{50} (mg/kg.bw)	TI (mg/kg.bw)
**PPEF**	Oral	300,500,1000,1500, 1800, 2000	>2000	ND	ND
**BPVF**	Oral	300,500,1000,1500, 1800, 2000	>2000	ND	ND
**PPEF**	Intravenous	10,25,50,80,100,125	>125	10	12.5
**BPVF**	Intravenous	10,25,50,80,100,125	>125	20	6.25

Supplementary Movies

Supplementary Movie 1. The simulation movie of EcTopoIA

Supplementary Movie 2. The simulation movie of EcTopoIA-ssDNA complex for 100 ns

Supplementary Movie 3. The simulation movie of EcTopoIA-PPEF complex for 100 ns.

Supplementary Movie 4. The simulation movie of EcTopoIA-ssDNA-PPEF complex (ternary complex) for 100 ns.

Supplementary Movie 5. The simulation movie of EcTopoIA-dsDNA complex for 100 ns.

Supplementary Movie 6. The simulation movie of EcTopoIA-dsDNA-PPEF complex (ternary complex) for 100 ns.

Materials Details: List of the software and the reagent used in the study with their Source (Company name), Catalogue number, and Unique RRID code.

S.No.	Reagent	Source	Identifier
		**Software**	
1.	Schrodinger	Schrodinger	RRID: SCR_014879
2.	AmberTools20	AmberTools20	RRID:SCR_018497
3.	MODELLER	MODELLER	RRID:SCR_008395
4.	Image J	NIH	RRID: SCR_003070
5.	Graphpad 7.0	Graphpad prism	RRID: SCR_002798
6.	Phoenix WinNonlin6.3	Phoenix WinNonlin6.3	---
7.	FastQC	FastQC	RRID:SCR_011106
8.	Trim Galore	Trim Galore	RRID:SCR_011847
9.	bowtie2 – 2.2.5	bowtie2 – 2.2.5	---
10.	Samtools version1.8	Samtools version1.8	RRID:SCR_002105
11.	Bcftools	Bcftools	---
12.	Vcftools	Vcftools	VCFtools, RRID:SCR_001235
13.	SnpEff	SnpEff	SnpEff, RRID:SCR_005191
		**Chemicals and reagent**	
14.	Tetracycline	HIMEDIA	Cat#EM056

	Ezy MIC strip		
15.	Cefoxitin Ezy MIC strip	HIMEDIA	Cat#EM101
16.	Clindamycin Ezy MIC strip	HIMEDIA	Cat#EM019
17.	Gentamicin Ezy MIC strip	HIMEDIA	Cat#EM025
18.	Co-Trimoxazole Ezy MIC strip	HIMEDIA	Cat#EM083
19.	Cefotaxime Ezy MIC strip	HIMEDIA	Cat#EM064
20.	Ampicillin Ezy MIC strip	HIMEDIA	Cat#EM068
21.	Chloramphenicol Ezy MIC strip	HIMEDIA	Cat#EM016
22.	Colistin Ezy MIC strip	HIMEDIA	Cat#EM02
23.	Ofloxacin Ezy MIC strip	HIMEDIA	Cat#EM039
24.	Ciprofloxacin Ezy MIC strip	HIMEDIA	Cat#EM082
25.	Kanamycin Ezy MIC strip	HIMEDIA	Cat#EM026
26.	Luria Bertani Broth, Miller	HIMEDIA	Cat#M1245
27.	Luria Bertani Agar, Miller	HIMEDIA	---
28.	MHB	HIMEDIA	---
29.	MHA	HIMEDIA	---
30.	Agarose	Lonza	Cat#50004
31.	Ciprofloxacin hydrochloride	Sigma-Aldrich	CAS Number: 93107-08-5
32.	Levofloxacin HCl	Real Gene	
33.	Kplus DNA Ladder RTU (Ready-to-Use)	GeneDireX, Inc.	---
34.	Prestained protein ladder	Blue ray GeneDireX	Cat# PM006-0500
35.	MTT	Sigma	Cat# M2128-1G
36.	Ethidium Bromide	Sigma	Cat# E8751
		**Plasmids**	
37.	pET28a+	Addgene	RRID:Addgene_60012
38.	pHOT-1	TopoGen	Cat#TG2030-1
		**Cell lines**	
39.	NIH3T3 mouse fibroblast cell line	NCCS, Pune, India.	---
40.	human embryonic kidney cell line HEK-293	NCCS, Pune, India.	---
		**Bacterial Cell**	
41.	*S. aureus* (MRSA)	ATCC	ATCC43300
42.	*Escherichia coli*	ATCC	ATCC25922
43.	*Enterococcus*	NCCS	MCC 2105
		**Animal**	
44.	SWISS mice	JNU, Animal House	---

45.	BALB/C	JNU, Animal House	---
