# Supplementary material for: Unraveling topoisomerase IA gate dynamics in presence of PPEF and its preclinical evaluation against multidrug-resistant pathogens
Source: Commun Biol. 2023 Feb 18;6:195. doi: 10.1038/s42003-023-04412-1 (PMC9938908; doi:10.1038/s42003-023-04412-1)
Supplement: Supplementary file 2 — Description of additional supplementary files [file 42003_2023_4412_MOESM2_ESM.docx]

**Description of Additional Supplementary Files**

File name: Supplementary Data 1

Description: Free energy data points of topoisomerase open closed dynamics in presence and absence of PPEF and nucleic acid.

File name: Supplementary Data 2

Description: MIC and MBC of PPEF against MDR pathogens. PPEF and BPVF effect on SaTopoIA mediated cleavage of pHOT1 plasmid DNA.

File name: Supplementary Data 3

Description: Inhibition of relaxation activity of EcTopoIA, SaTopoIA, AbTopoIA in the presence of PPEF and BPVF

File name: Supplementary Data 4

Description: The length of E. coli treated cells at 1.5, 3, and 4.5 hr in presence of PPEF and BPVF. Time-killing assay of *S. aureus* and *E. coli* in presence and absence of PPEF and BPVF. Resistance acquisition during serial passaging in the presence of sub-MIC levels of antimicrobials. Frequency of resistance with and without PPEF, BPVF, and levofloxacin.

File name: Supplementary Data 5

Description: The accumulation and efflux assay of PPEF or BPVF in E. coli K12 (MG1655), ΔyciM (MG1655), lpxC (MG1655), and lpxD (MG1655) strains. Biofilm inhibition assay of S. aureus (ATCC 43300) in the presence of different concentrations of PPEF, BPVF, and CIP.

File name: Supplementary Data 6

Description: The efficacy of PPEF and BPVF against Gram-positive bacteria (MRSA) in murine sepsis and neutropenic thigh infection model.

File name: Supplementary Data 7

Description: Equilibration and metadynamic conformation of TopoIA, TopoIA-dsDNA, TopoIA-dsDNA-PPEF, TopoIA-PPEF, TopoIA-ssDNA, TopoIA-ssDNA-PPEF and the PPEF topology.

Supplementary Movie 1. The simulation movie of EcTopoIA

Supplementary Movie 2. The simulation movie of EcTopoIA-ssDNA complex for 100 ns

Supplementary Movie 3. The simulation movie of EcTopoIA-PPEF complex for 100 ns.

Supplementary Movie 4. The simulation movie of EcTopoIA-ssDNA-PPEF complex (ternary complex) for 100 ns.

Supplementary Movie 5. The simulation movie of EcTopoIA-dsDNA complex for 100 ns.

Supplementary Movie 6. The simulation movie of EcTopoIA-dsDNA-PPEF complex (ternary complex) for 100 ns.
